# Supplementary material for: Design, Synthesis, In Silico Studies and In Vitro Evaluation of New Indole- and/or Donepezil-like Hybrids as Multitarget-Directed Agents for Alzheimer’s Disease
Source: Pharmaceuticals (Basel). 2023 Aug 22;16(9):1194. doi: 10.3390/ph16091194 (PMC10534827; doi:10.3390/ph16091194)
Supplement: Supplementary file 1 [file pharmaceuticals-16-01194-s001.zip › pharmaceuticals-2549678-supplementary.pdf]

Supporting information

Design, Synthesis, In Silico Studies and In Vitro Evaluation of New Indole- and/or Donepezil-like Hybrids as Multitarget-Directed Agents for Alzheimer's Disease

Corresponding author: Violina T. Angelova; E-mail: v.stoyanova@pharmfac.mu-sofia.bg

Key NOESY correlations of hydrazide-hydrazones **3c** and **3o**..........2S

Molecular docking of compound **5a** in the active site of AChE (PDB ID 4EY7) 2S

Visualization of the pocket of MT1 and MT2 receptors as well as detailed data of the docking results..........3S

^{1}H NMR, ^{13}C NMR and HRMS spectra **3a-r**.......... 10S

^{1}H NMR, ^{13}C NMR and HRMS spectra for **5a-l**.......... 60S

References.......... 81S

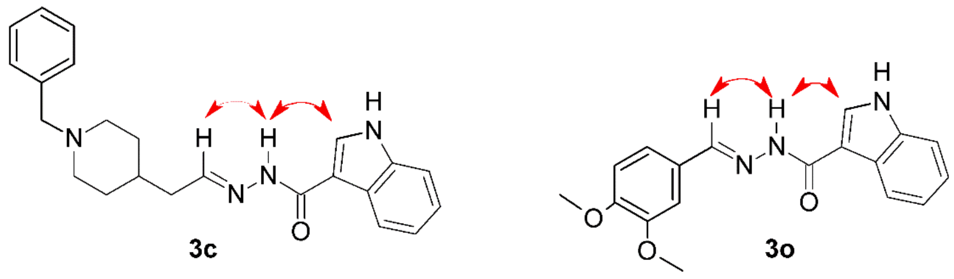

Figure S1. Key NOESY correlations of hydrazide-hydrazones **3c** and **3o**

Molecular docking of compound 5a in the active site of AChE (PDB ID 4EY7)

a)

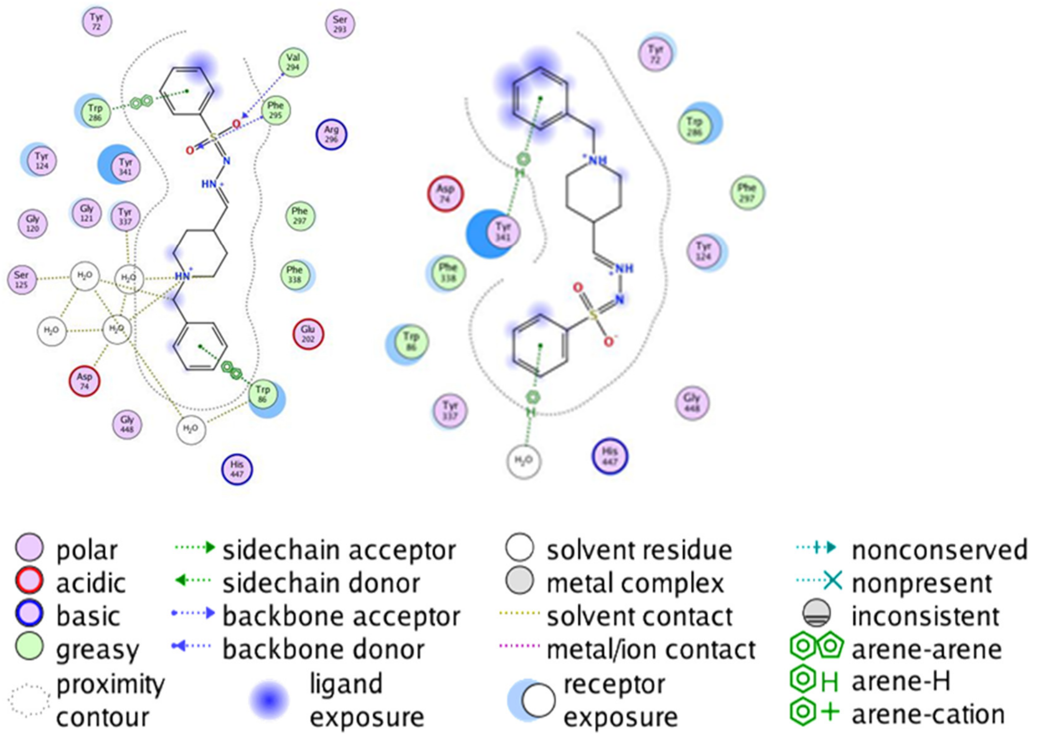

b)

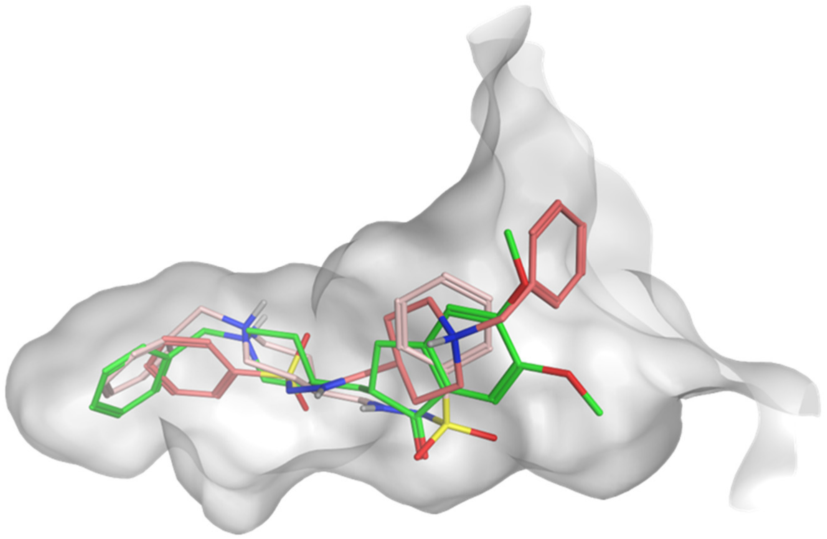

Figure S2. Docking results of compound **5a**: a) left – PLIs of the highest ranked pose; right – PLI of the second highest ranked pose; b) the best pose of donepezil (green), the highest ranked pose (light pink) and the second highest ranked pose (dark pink) in the active site of AChE (PDB ID 4EY7)

Visualization of the pocket of MT1 and MT2 receptors as well as detailed data of the docking results

Table S1. RMSD of CA of the active site residues between aligned chains of selected PDB structures for MT1 modeling

Chains	1	2	3	4	5	6	7
1:6ME2.A		0.18	0.21	0.22	1.1	1.04	0.84
2:6ME3.A	0.18		0.22	0.21	1.13	1	0.84
3:6ME4.A	0.21	0.22		0.23	1.12	1.01	0.85
4:6ME5.A	0.22	0.21	0.23		1.12	1.04	0.88
5:7DB6.D	1.1	1.13	1.12	1.12		1.4	1.23
6:7VGY.A	1.04	1	1.01	1.04	1.4		0.72
7:7VGZ.B	0.84	0.84	0.85	0.88	1.23	0.72	

Table S2. Similarity of PDB sequences of selected PDB structures for modeling MT1 receptor according to BLUSSUM62

Chains	1	2	3	4	5	6	7
1:6ME2.A		100	100	100	96.4	96.4	96.4
2:6ME3.A	100		100	100	96.4	96.4	96.4
3:6ME4.A	100	100		100	96.4	96.4	96.4
4:6ME5.A	100	100	100		96.4	96.4	96.4
5:7DB6.D	96.4	96.4	96.4	96.4		100	100
6:7VGY.A	96.4	96.4	96.4	96.4	100		100
7:7VGZ.B	96.4	96.4	96.4	96.4	100	100	

Table S3. RMSD of CA of the active site residues between aligned chains of selected PDB structures for MT2 modeling

Chains	1	2	3	4	5	6	7	8	9	10
1:6ME6.A		0.12	0.3	0.31	0.23	0.25	0.28	0.29	0.53	0.9
2:6ME6.B	0.12		0.35	0.31	0.23	0.22	0.3	0.28	0.52	0.95

3:6ME7.A	0.3	0.35		0.14	0.36	0.39	0.41	0.42	0.61	0.91
4:6ME7.B	0.31	0.31	0.14		0.36	0.36	0.42	0.41	0.61	0.96
5:6ME8.A	0.23	0.23	0.36	0.36		0.08	0.17	0.18	0.54	0.93
6:6ME8.B	0.25	0.22	0.39	0.36	0.08		0.2	0.18	0.55	0.96
7:6ME9.A	0.28	0.3	0.41	0.42	0.17	0.2		0.09	0.58	0.94
8:6ME9.B	0.29	0.28	0.42	0.41	0.18	0.18	0.09		0.58	0.96
9:6PS8.A	0.53	0.52	0.61	0.61	0.54	0.55	0.58	0.58		0.95
10:7VH0.A	0.9	0.95	0.91	0.96	0.93	0.96	0.94	0.96	0.95	

Table S4. Similarity of PDB sequences of selected PDB structures for modeling MT2 receptor according to BLUSSUM62

Chains	1	2	3	4	5	6	7	8	9	10
1:6ME6.A		98.6	99	98.3	99	98.6	98.6	97.9	81.8	96.4
2:6ME6.B	99		98.6	98.3	98.6	98.6	98.3	97.9	81.8	96.4
3:6ME7.A	98.6	97.9		98.6	98.3	97.9	97.9	97.2	81.1	96
4:6ME7.B	98.3	97.9	99		97.9	97.9	97.6	97.2	81.1	96
5:6ME8.A	99.3	98.6	99	98.3		98.6	98.6	97.9	81.8	96.4
6:6ME8.B	99	98.6	98.6	98.3	98.6		98.3	97.9	81.8	96.4
7:6ME9.A	99.3	98.6	99	98.3	99	98.6		98.6	81.8	96.4
8:6ME9.B	99	98.6	98.6	98.3	98.6	98.6	99		81.8	96.4
9:6PS8.A	81.5	81.2	81.1	80.8	81.2	81.2	81	80.7		81.3
10:7VH0.A	93.4	93.1	93.4	93	93.1	93.1	92.7	92.4	79	

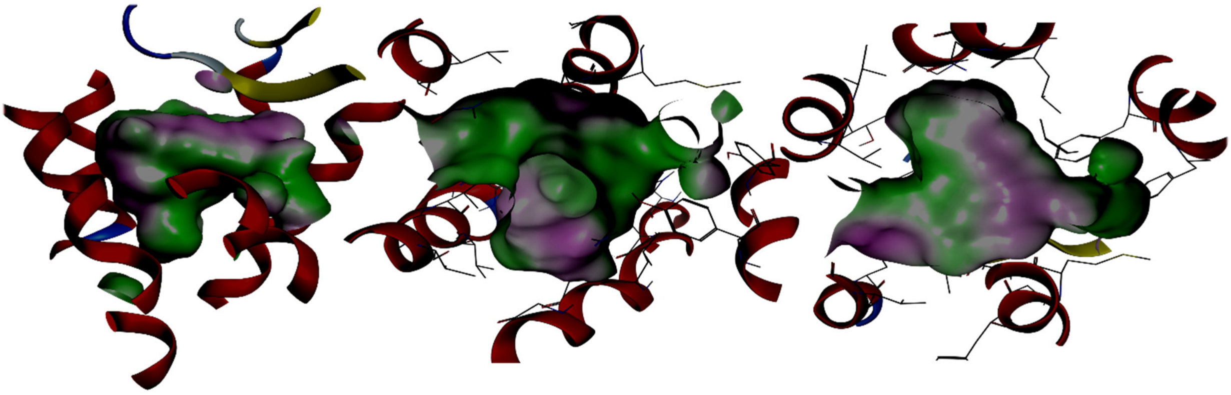

Figure S3. Side, up and down view, respectively, of the pocket that forms the active site in our model of the MT1 receptor (lipophilic parts of the pocket are in green, while hydrophilic are represented in pink)

Table S5. The best results from induced fit docking of ligands on MT1 receptor according to GBVI/WSA scoring function (kcal/mol)

Name	Formula	Score
**3a**	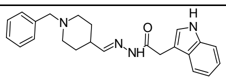	-27.1
**3b**	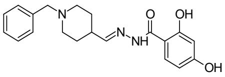	-23.0
**3c**	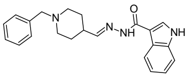	-26.7
**3d**	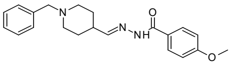	-23.0
**3i**	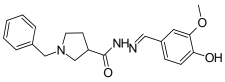	-25.8
**3m**	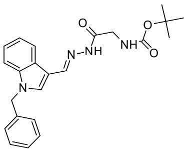	-22.4
**3n**	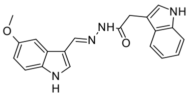	-20.1
**5a**	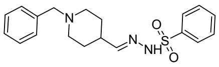	-26.0

**5d**	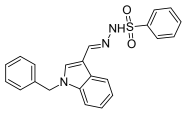	-20.8
**5g**	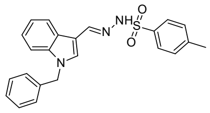	-21.8
**5j**	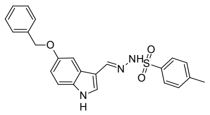	-22.9

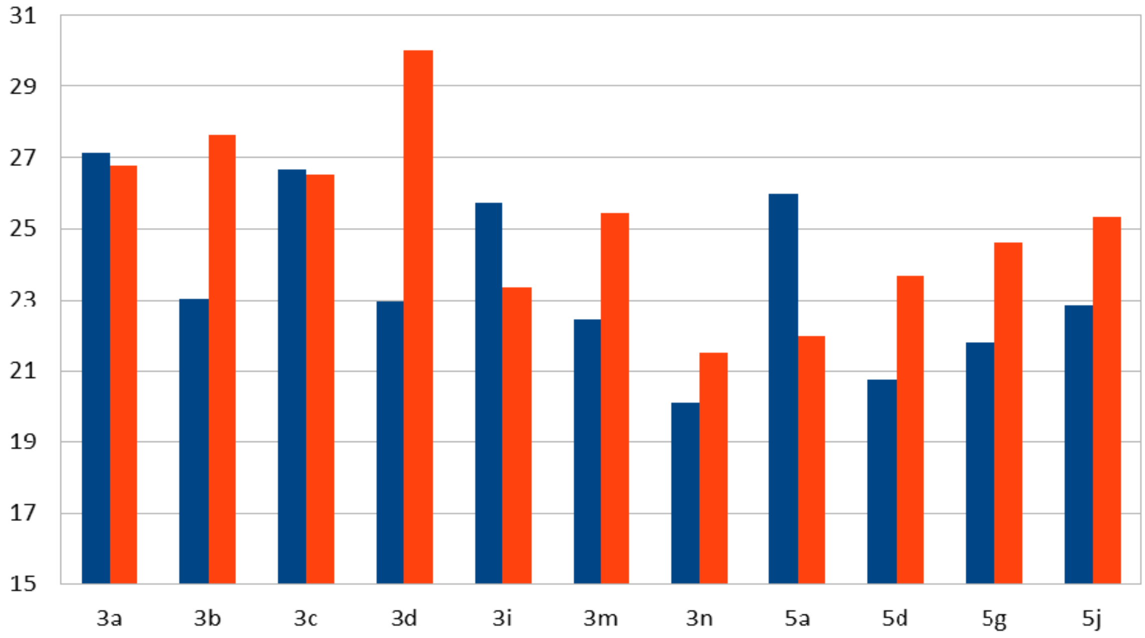

Figure S4. The best interaction energies of our ligands with the MT1 (blue) and MT2 (red) receptors according to GBVI/WSA scoring function (kcal/mol)

In order to elucidate functional groups that are essential for receptor-ligand interactions in our case a Protein Ligand Interaction Fingerprints (PLIF) analysis was performed. Being method for summarizing the interactions between ligands and receptor using a fingerprint scheme it reveals which binding site residues tends to interact with which parts of the ligands.

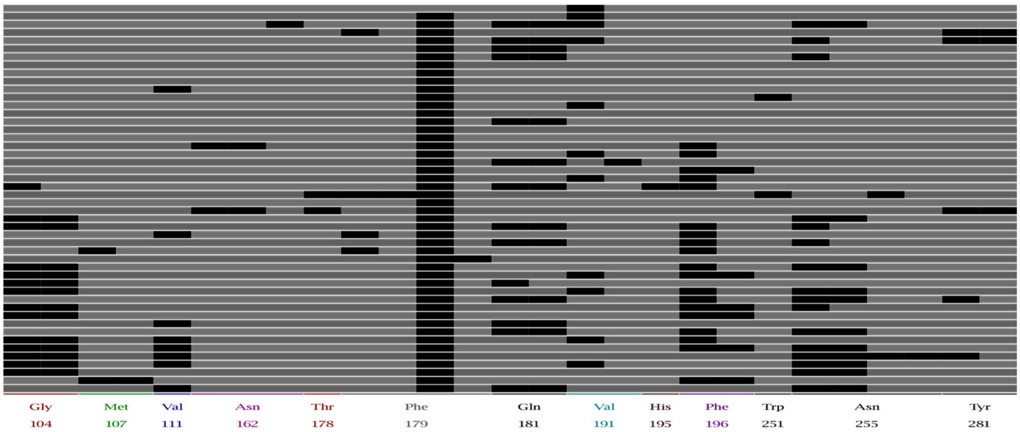

Figure S5. PLIF Barcode map of interaction between the best 50 ligands poses and amino acids of the active site cavity of the MT1 receptor.

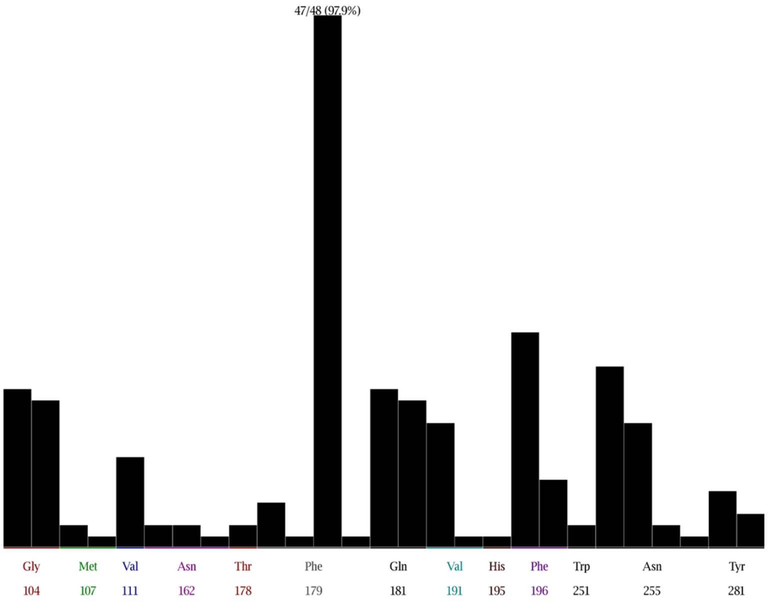

Figure S6. Population histogram of the interactions of the first 50 ligand poses with residues inside active site of the receptor according to PLIF analysis (Phe179)

MT2 receptor docking results

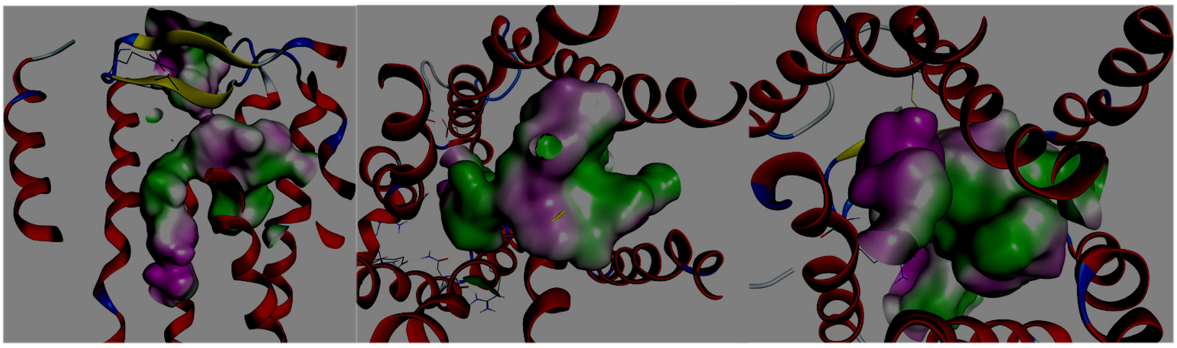

Figure S7. Side, up and down view, respectively, of the pocket that forms the active site in our model of the MT2 receptor (lipophilic parts of the pocket are in green, while hydrophilic are represented in pink)

Table S6. The best results from induced fit docking of ligands on MT2 receptor according to GBVI/WSA scoring function (kcal/mol)

Name	Formula	Score
**3a**	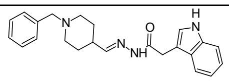	-26.8
**3b**	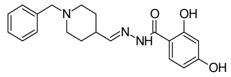	-27.6
**3c**	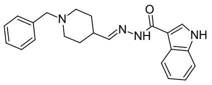	-26.5
**3d**	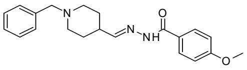	-30.0
**3i**	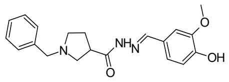	-23.4
**3m**	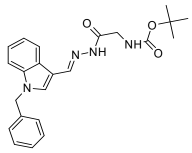	-25.4
**3n**	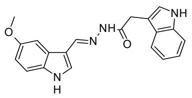	-21.5

**5a**	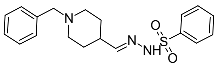	-22.0
**5d**	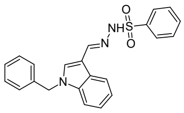	-23.7
**5g**	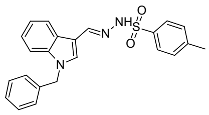	-24.6
**5j**	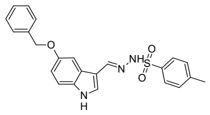	-25.4

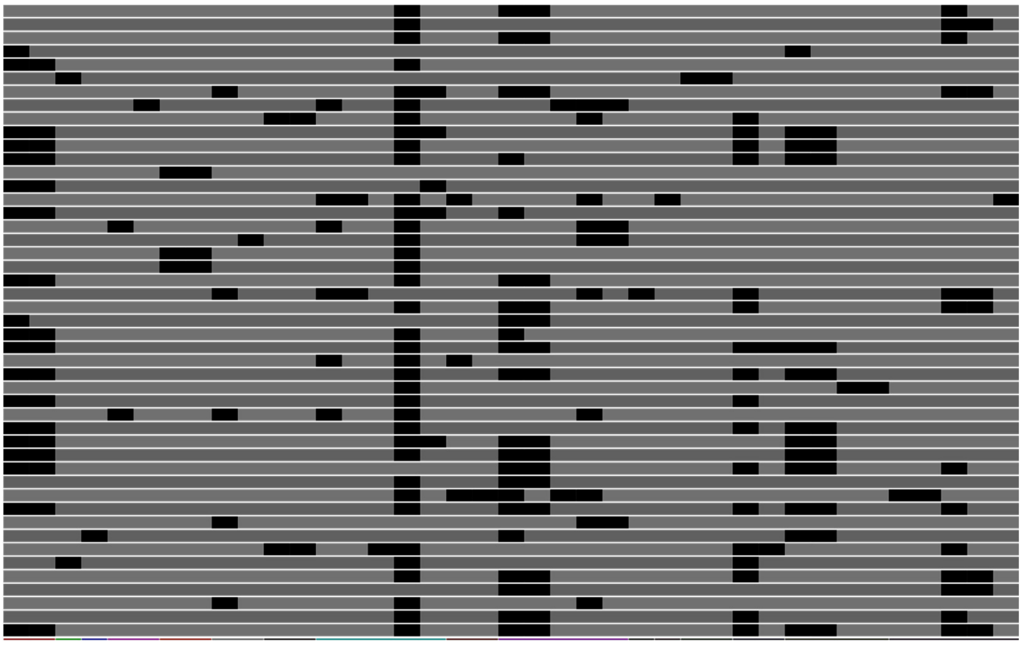

Figure S8. Barcode map of interaction between best 50 ligands poses and amino acids of the active site cavity of the MT2 receptor

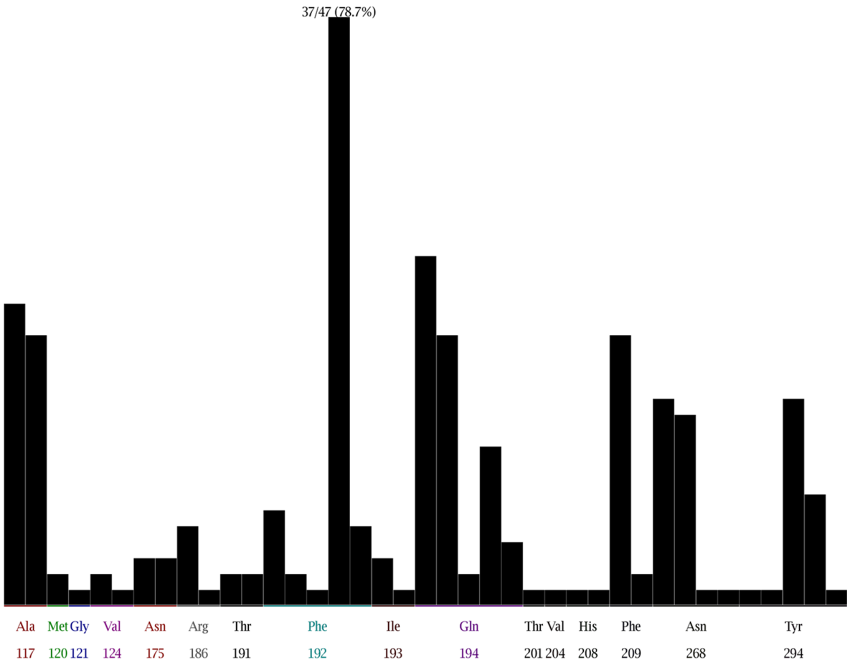

Figure S9. Population histogram of the interactions of the first 50 ligand poses with residues inside the active site of the receptor according to PLIF analysis

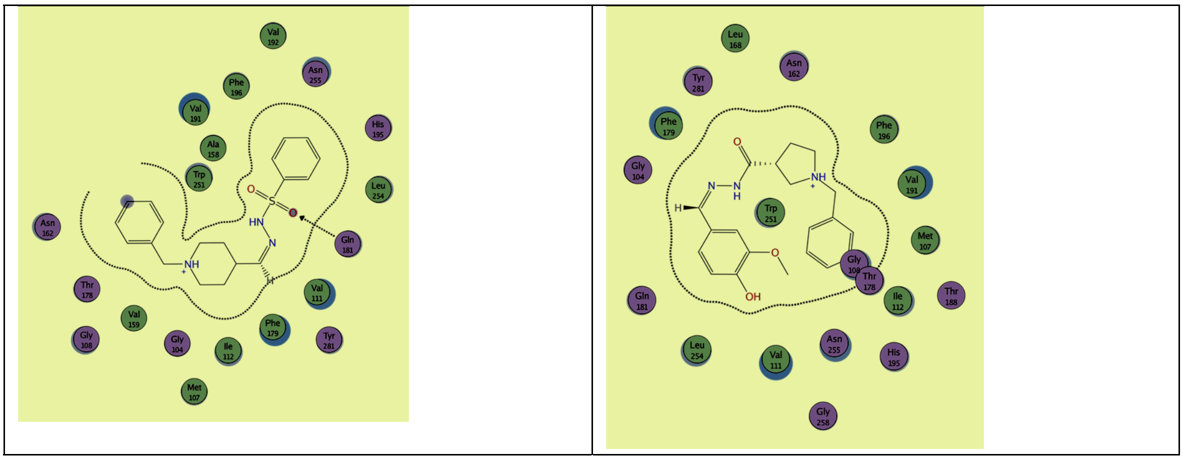

Figure S10. Interaction maps of the best poses of the third (**5a** left) and forth (**3i** right) of the best ligands for MT1 receptor

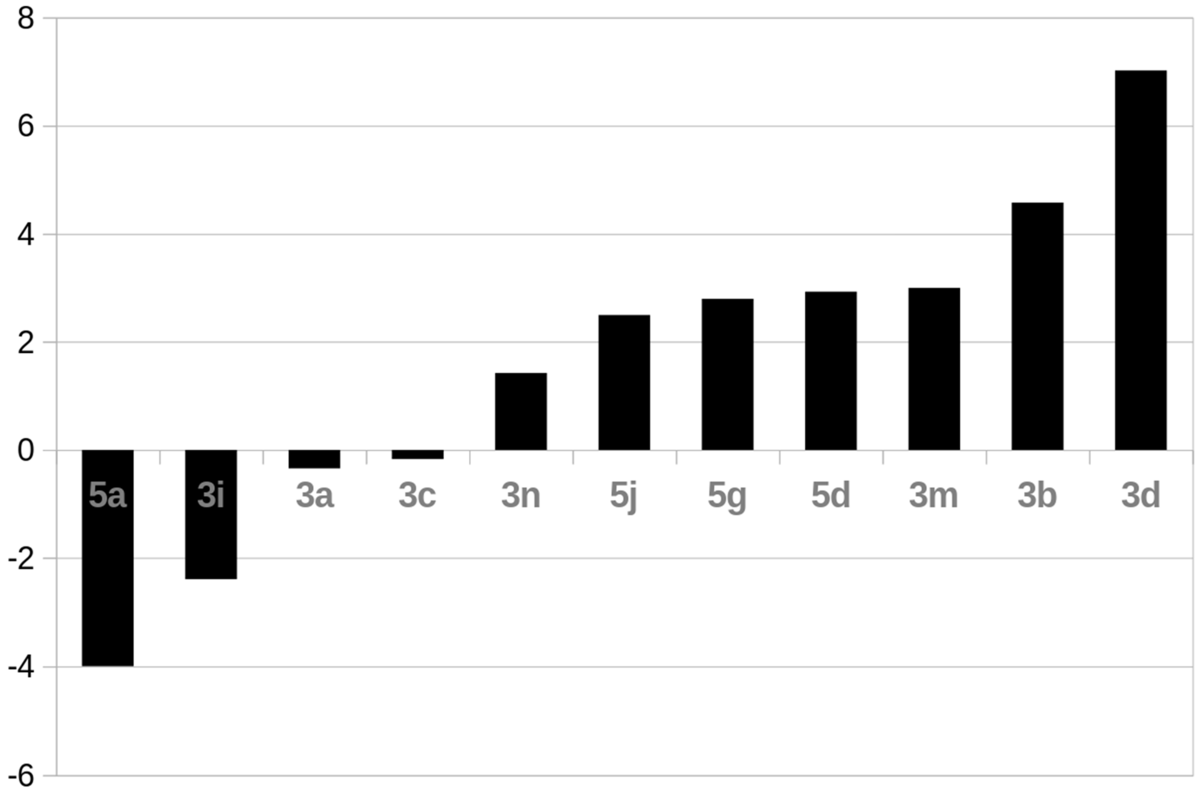

Figure S11. Difference between MT2 and MT1 receptor binding energies of our ligands (kcal/mol). Ligands with negative values prefer to bind to MT1, while ligands with positive values prefer to bind to MT2.

^{1}H NMR, ^{13}C NMR and HRMS spectra

1. ***N'-[(E)-(1-benzylpiperidin-4-yl)methylidene]-2-(1H-indol-3-yl)acetohydrazide,* 3a**

Yield: 68%; m.p. 167-168◦C. 1H NMR (400 MHz, DMSO-d6): 1:0.83 mixture of conformers; signals for major synperiplanar conformer around the amide bond: δ = 1.42-1.53 (m, 2H, CH2), 1.67-1.78 (m, 2H, CH2), 1.94-2.04 (m, 2H, CH2), 2.17-2.24 (m, 1H, H-4'), 2.77-2.82 (m, 2H, CH2), 3.46 (s, 2H, CH2), 3.90 (s, 2H, CH2), 6.98 (ddd, J=1.00, 7.0, 8.0 Hz, 1H, H-5), 7.05 (ddd, J=1.0, 7.0, 5.0 Hz, 1H, H-6), 7.17 (d, J=2.3 Hz, 1H, H-2), 7.22-7.33 (m, 10H, H-2'', H-3'', H-4'', H-5'' and H-6''), 7.35 (d, J=7.0 Hz, 1H, H-7), 7.44 (d, J=5.3 Hz, 1H, CH), 7.53 (d, J=8.2 Hz, 1H, H-4), 10.84 (s, 1H, NH), 10.85 (bs, 1H, NH); resolved signals for minor antiperiplanar conformer around the amide bond: 3.45 (s, 2H, CH2), 3.53 (s, 2H, CH2), 6.96 (ddd, J=1.0, 7.0, 8.0 Hz, 1H, H-5), 7.07 (ddd, J=1.0, 7.0, 5.0 Hz, 1H, H-6), 7.20 (d, J=2.3 Hz, 1H, H-2), 10.89 (bs, 1H, NH), 11.06 (s, 1H, NH); 13C NMR (100 MHz, DMSO-d6): signals for major synperiplanar conformer around the amide bond: δ = 28.85 (CH2), 29.06 (CH2), 38.10 (C-4'), 52.45 (CH2), 62.39 (CH2), 108.22 (C-3), 111.24 (C-7), 118.19 (C-5), 118.65 (C-4), 120.84 (C-6), 120.95 (C-5), 123.83 (C-2),

126.80 (C-4''), 127.14 (C-4a), 127.39 (C-4a), 128.11 (C-3'' and C-5''), 128.73 (C-2'' and C-6''), 135.95 (C-7a), 138.51 (C-1''), 149.21 (CH), 166.66 (C=0); resolved signals for minor antiperiplanar conformer around the amide bond: 152.96 (CH), 172.20 (C=O). HRMS (ESI) *m/z*: calcd: [M+H]+ 375.217938. Found: [M+H]+ 375.2178

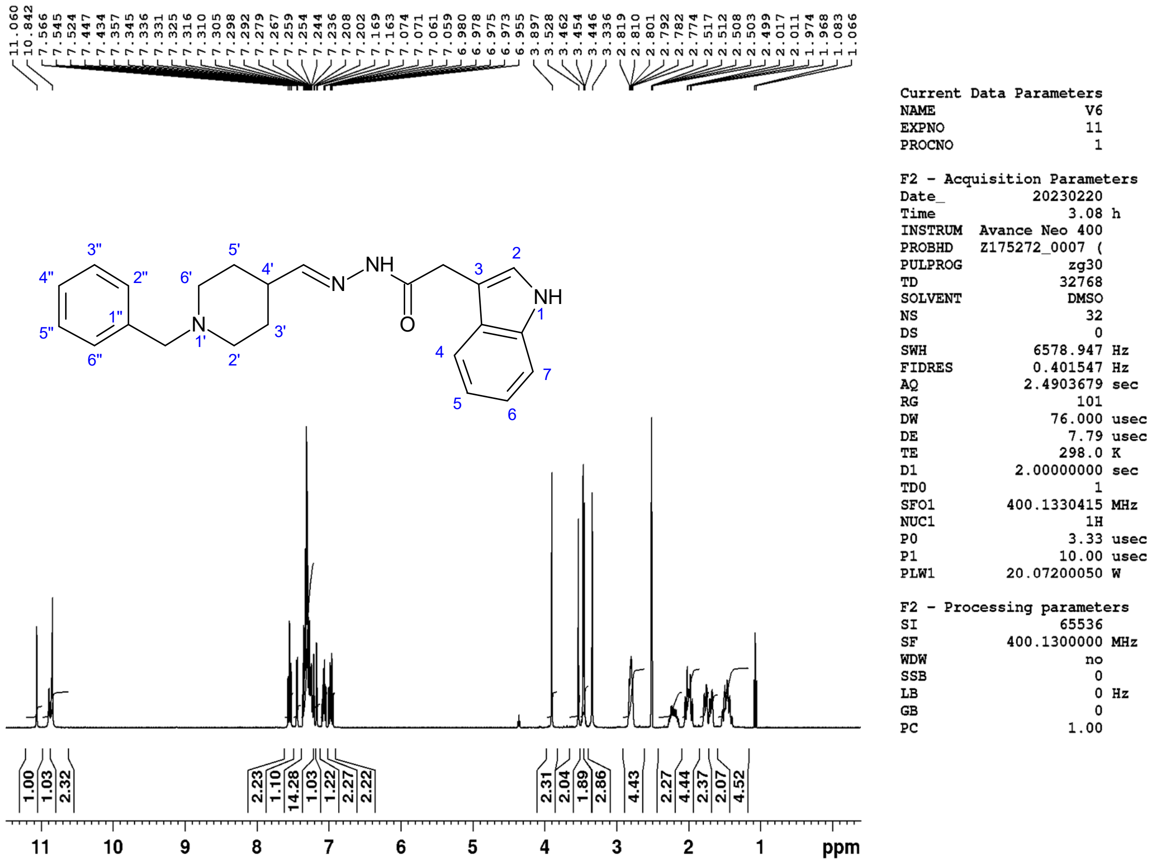

Figure S12. ^{1}H NMR spectrum of compound **3a** in DMSO-d_6

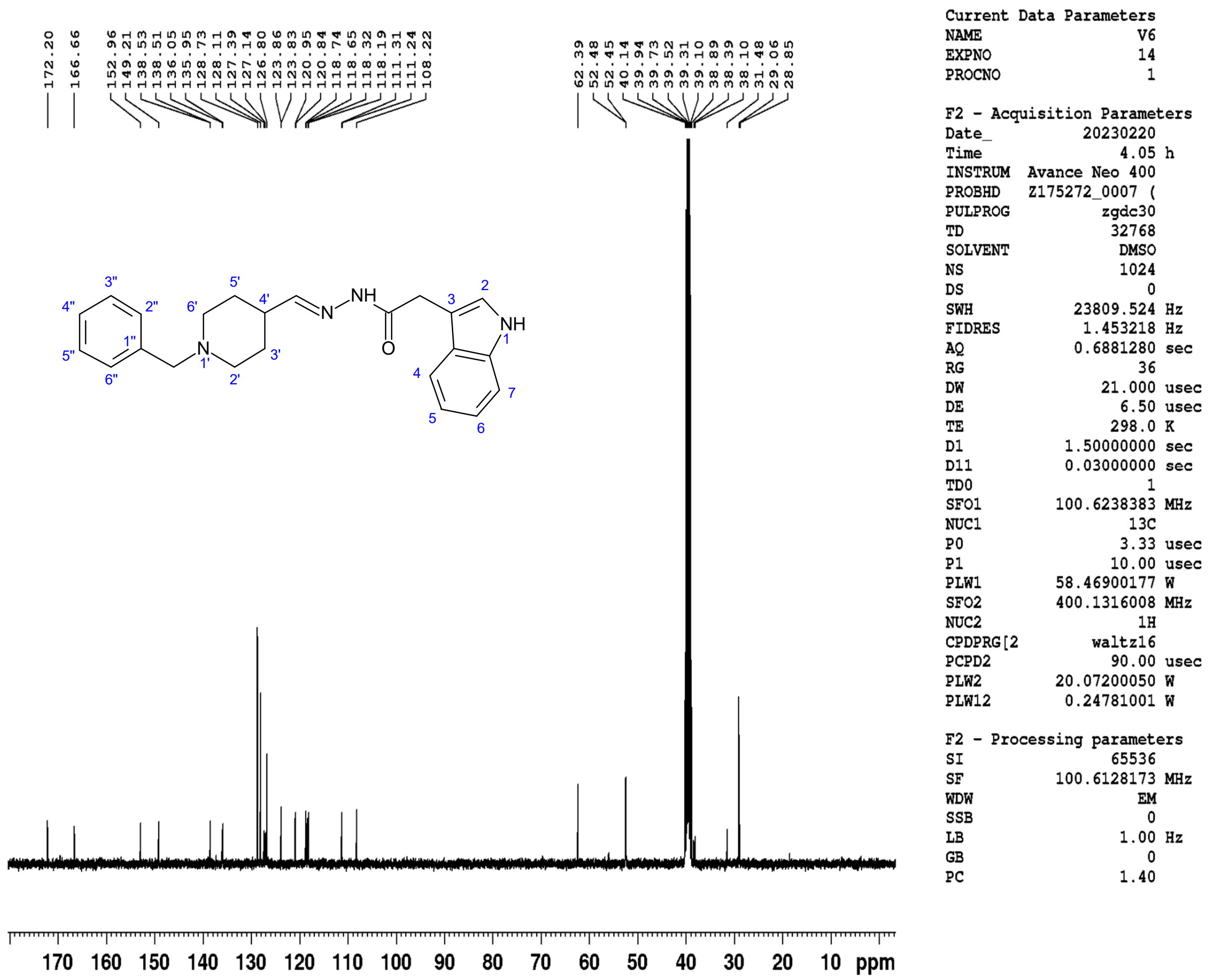

Figure S13. ^{13}C NMR spectrum of compound **3a** in DMSO-d_6

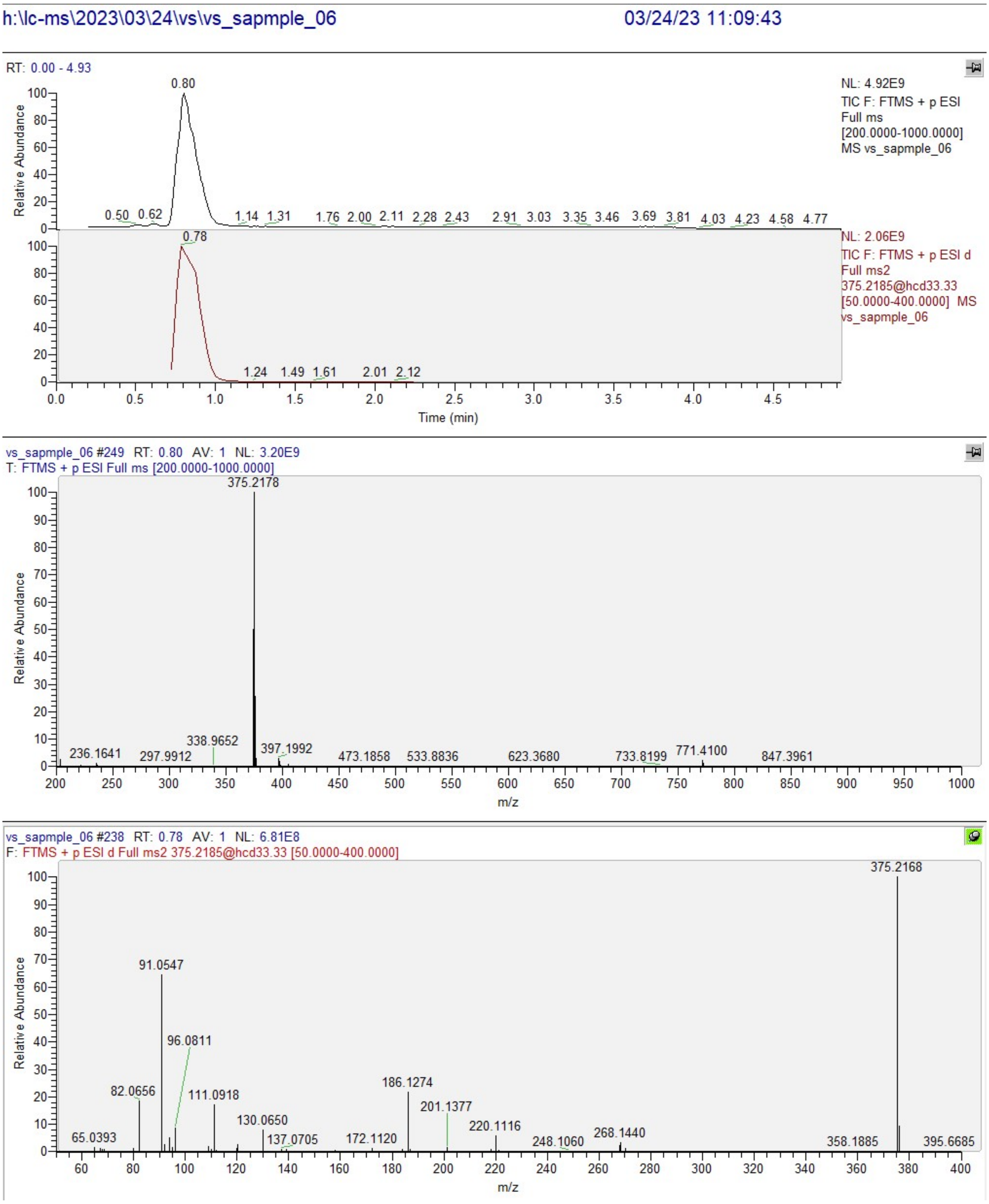

Figure S14. HRMS of compound **3a**

2. *N'-[(E)-(1-benzylpiperidin-4-yl)methylidene]-2,4-dihydroxybenzohydrazide,* 3b

Yield: 59%; m.p. 201-203◦C.^{1}H NMR (400 MHz, DMSO-d_6): δ = 1.46 (dd, J=3.1, 11.7, 1H) and 1.52 (dd, J=3.1, 11.7 Hz, 1H, CH_2), 1.74 (dd, J=2.7, 13.5 Hz, 2H, CH_2), 2.00-2.05 (m, 2H, CH_2), 2.22-2.34 (m, 1H, H-4'), 2.82 (d, J=11.4 Hz, 2H, CH_2), 3.48 (s, 2H, CH_2), 6.27 (d, J=2.3 Hz, 1H, H-3), 6.32 (dd, J=2.3, 8.7 Hz, 1H, H-5), 7.23-7.35 (m, 5H, Ar), 7.67 (d, J=5.0 Hz, 1H, CH), 7.72 (d, J=8.7 Hz, 1H, H-6), 10.17 (bs, 1H, OH), 11.32 (bs, 1H, NH), 12.44 (bs, 1H, OH).
^{13}C NMR (100 MHz, DMSO-d_6): δ = 29.00 (C-3' and C-5'), 38.53 (C-4'), 52.42 (C-2' and C-6'), 62.36 (CH_2), 102.81 (C-3), 105.87 (C-1), 107.19 (C-5), 126.86 (C-4''), 128.13 (C-3'' and C-5''), 128.78 (C-2'' and C-6''), 129.35 (C-6), 138.41 (C-1''), 155.26 (CH), 162.49 and 162.53 (C-2 and C-4), 165.44 (C=O). HRMS (ESI) *m/z*: calcd: [M+H]+ 354.181218. Found: [M+H]+ 354,1811

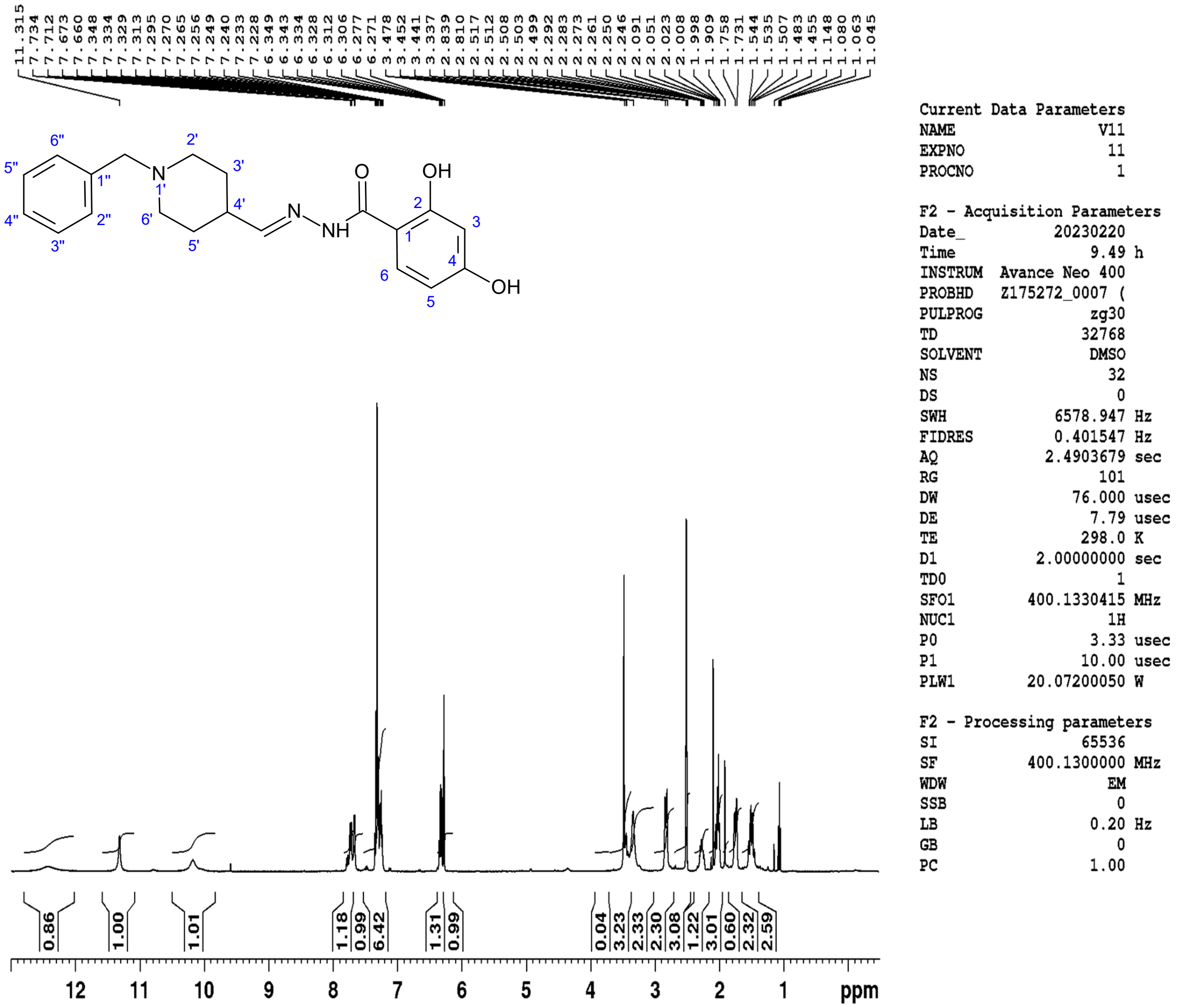

Figure S15. ^{1}H NMR spectrum of compound **3b** in DMSO-d_6

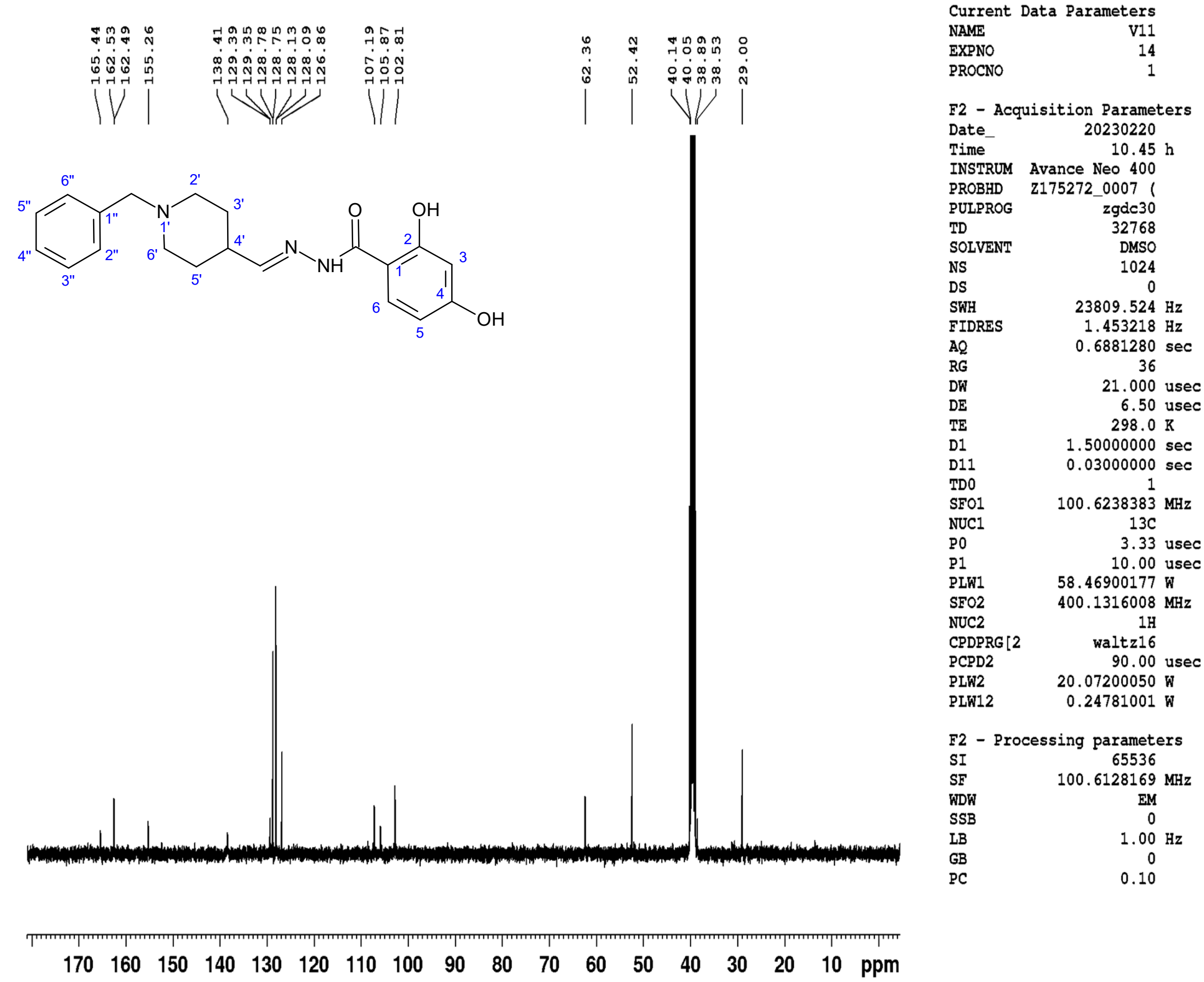

Figure S16. ^{13}C NMR spectrum of compound **3b** in DMSO-d_6

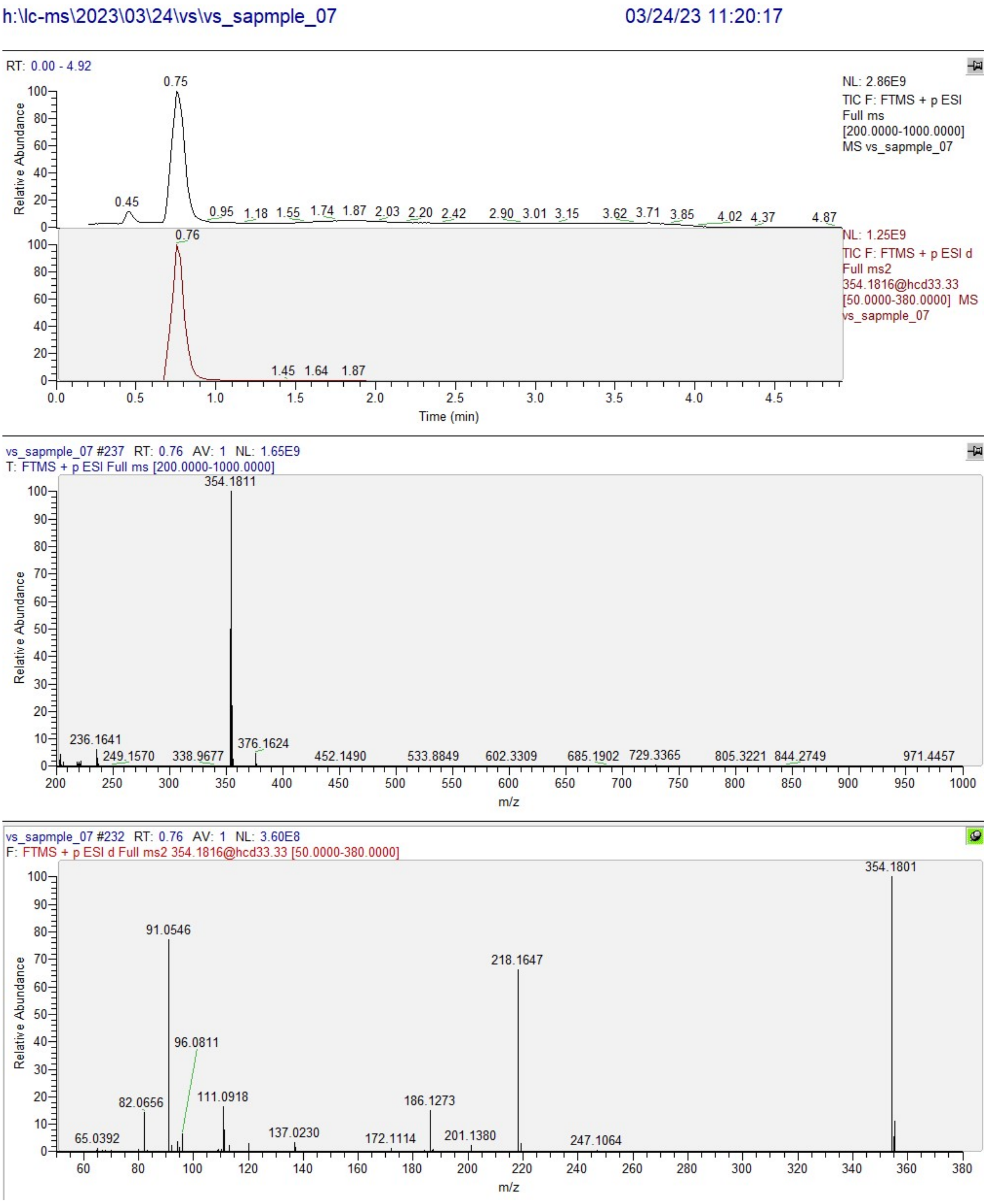

Figure S17. HRMS of compound **3b**

3. *N'-[(E)-(1-benzylpiperidin-4-yl)methylidene]-1H-indole-3-carbohydrazide,* 3c

Yield: 72%; m.p. 249-250◦C. ^{1}H NMR (400 MHz, DMSO-d$_6$, 363K): δ = 1.51 (dd, J=3.7, 11.3 Hz, 1H, CH_2), 1.57 (dd, J=3.7, 11.1 Hz, 1H, CH_2), 1.79 (dd, J=3.4, 12.9 Hz, 2H, CH_2), 2.09 (dt, J=2.6, 11.3 Hz, 2H, CH_2), 2.23-2.32 (m, 1H, H-4'), 2.82 (td, J=3.5, 11.7 Hz, 1H, CH_2), 3.49 (s, 2H, CH_2), 7.12 (dt, J=1.2, 10.9 Hz, 1H, H-5), 7.16 (dt, J=1.4, 7.5 Hz, 1H, H-6), 7.21-7.26 (m, 1H, H-4''), 7.29-7.32 (m, 4H, C-3'', C-5'', C-2'' and C-6''), 7.44 (d, J=7.9 Hz, 1H, H-7), 7.55 (d, J=4.8 Hz, 1H, CH), 8.12 (bs, 1H, H-2), 8.17 (d, J=7.3 Hz, 1H, H-4), 10.53 (bs, 1H, NH), 11.36 (bs, 1H, NH).

^{13}C NMR (100 MHz, DMSO-d$_6$, 363K): δ = 29.01 (C-3' and C-5'), 37.97 (C-4'), 52.19 (C-2' and C-6'), 62.11 (CH_2), 108.42 (C-3), 111.44 (C-7), 120.21 (C-5), 120.95 (C-4), 121.73 (C-6), 126.43 (C-4''), 126.63 (C-4a), 127.74 (C-3'' and C-3''), 128.44 (C-2'' and C-6''), 129.27 (C-2), 135.69 (C-7a), 138.34 (C-1''), 150.89 (CH), 162.35 (C=O). HRMS (ESI) m/z: calcd: [M+H]+ 361.202288. Found: [M+H]+ 361,2021

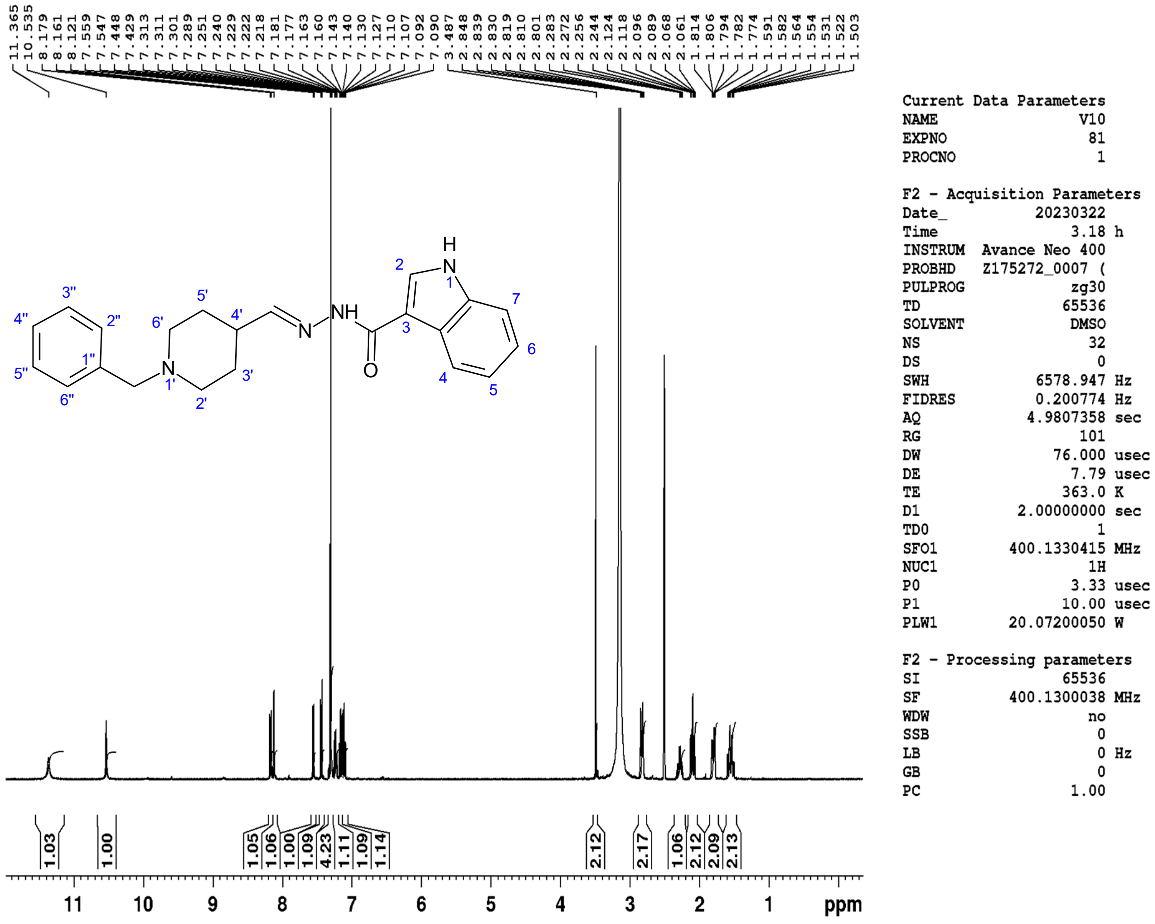

Figure S18. ^{1}H NMR spectrum of compound **3c** in DMSO-*d$_6$*

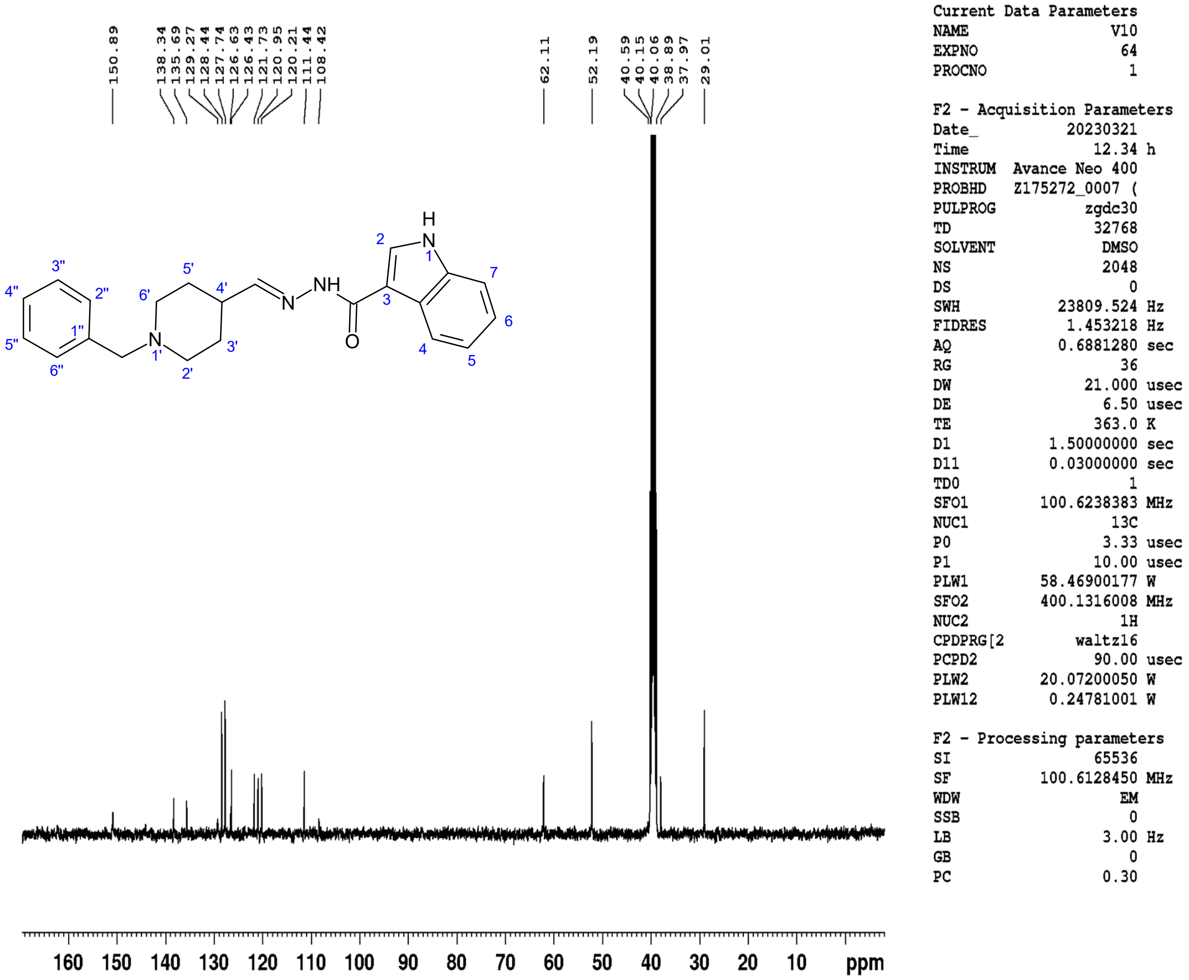

Figure S19. ^{13}C NMR spectrum of compound **3c** in DMSO-d_6

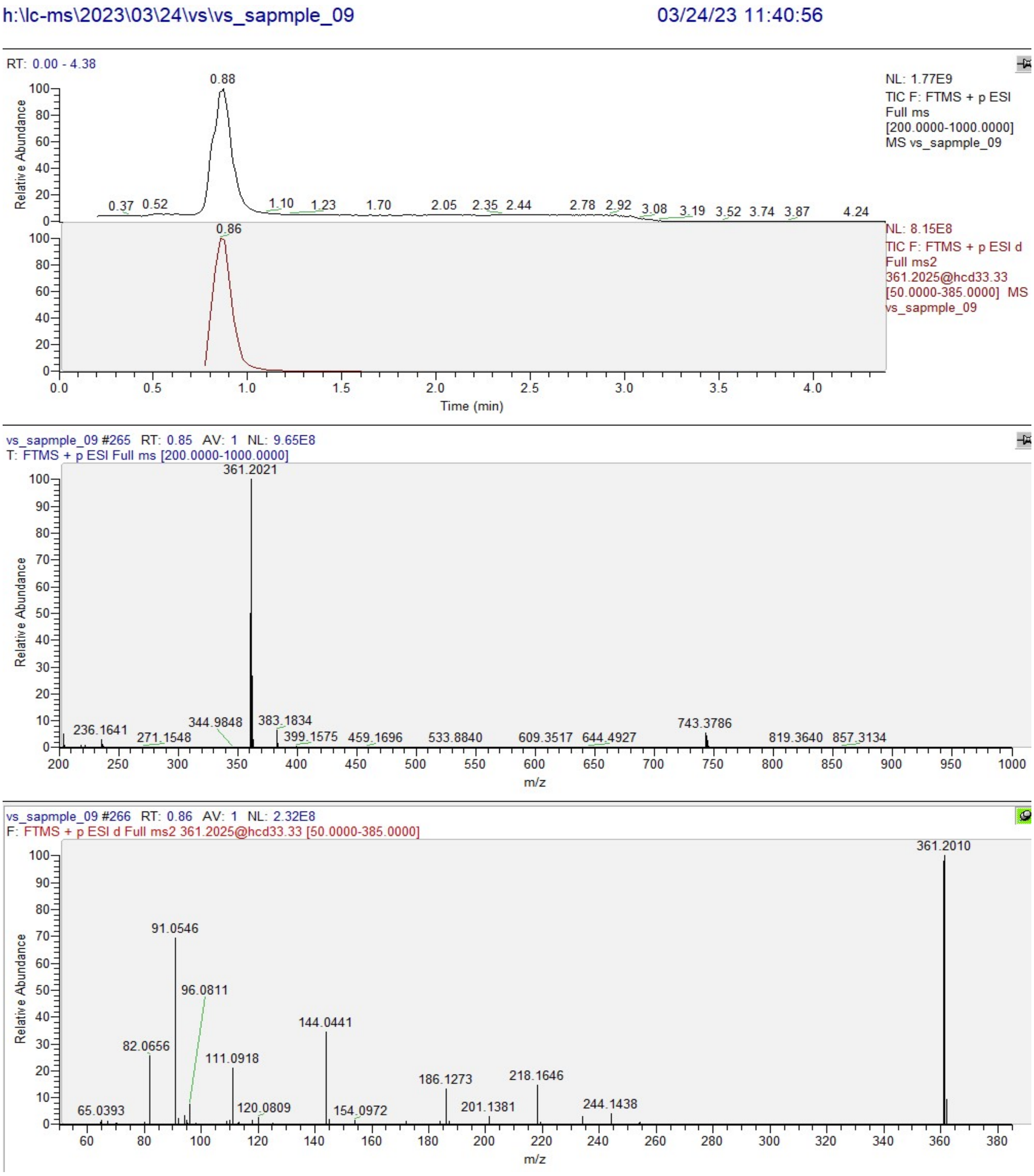

Figure S20. HRMS of compound **3c**

4. N'-[(E)-(1-benzylpiperidin-4-yl)methylidene]-4-methoxybenzohydrazide, 3d

Yield: 82%; m.p. 138-140◦C.^{1}H NMR (600 MHz, DMSO-d6): δ = 1.49 (q, J=10.9 Hz, 2H) and 1.74 (d, J=11.6 Hz, 2H, H-2' and H6'), 2.01 (t, J=11.0 Hz, 2H) and 2.82 (d, J=11.2 Hz, 2H, H-3' and H-5'), 2.25 (d, J=4.7 Hz, 1H, H-1'), 3.47 (s, 2H, CH_2), 3.82 (s, 3H, OCH_3), 7.02 (d, J=8.6 Hz, 2H, H-3 and H-5), 7.25 (tt, J=2.0, 6.7 Hz, 1H, H-4''), 7.30-7.34 (m, 4H, H-2'', H-3'', H-5'' and H-6''), 7.67 (d, J=5.1 Hz, 1H, CH), 7.84 (d, J=8.8 Hz, 2H, H-2 and H-6), 11.28 (s, 1H, NH).

^{13}C NMR (151 MHz, DMSO-d6): δ = 29.14 (C-2' and C-6'), 38.57 (C-1'), 52.48 (C-3' and C-5'), 55.37 (OCH_3), 62.42 (CH_2), 113.60 (C-3 and C-5), 125.58 (C-1), 126.82 (C-4''), 128.12 (C-3'' and C-5''), 128.76 (C-2'' and C-6''), 129.34 (C-2 and C-6), 138.51 (C-1''), 154.21 (CH), 161.80 (C-4), 162.23 (C=O). HRMS (ESI) m/z: calcd: [M+H]+ 352.201953. Found: [M+H]+ 352.20115

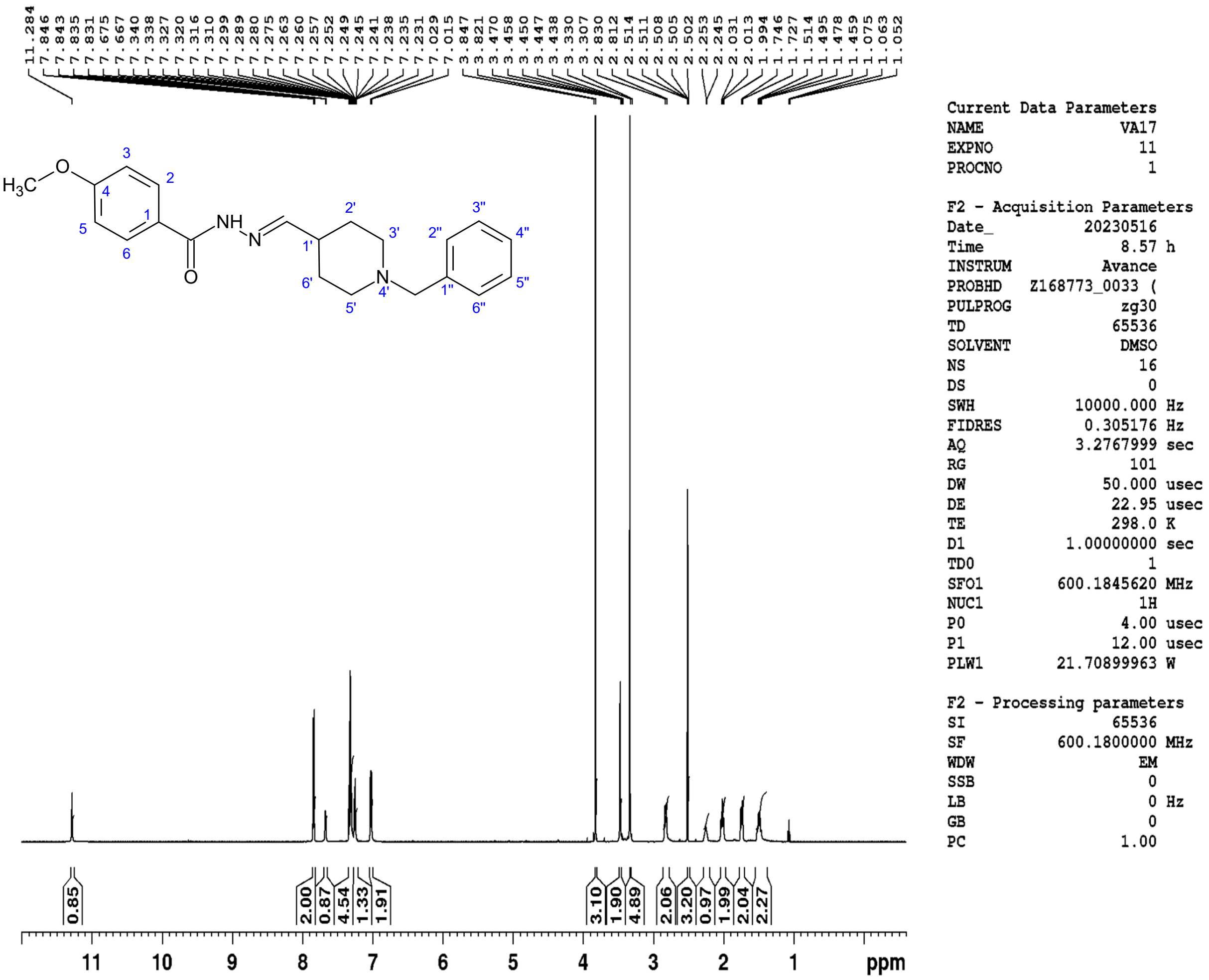

Figure S21. ^{1}H NMR spectrum of compound **3d** in DMSO-d_6

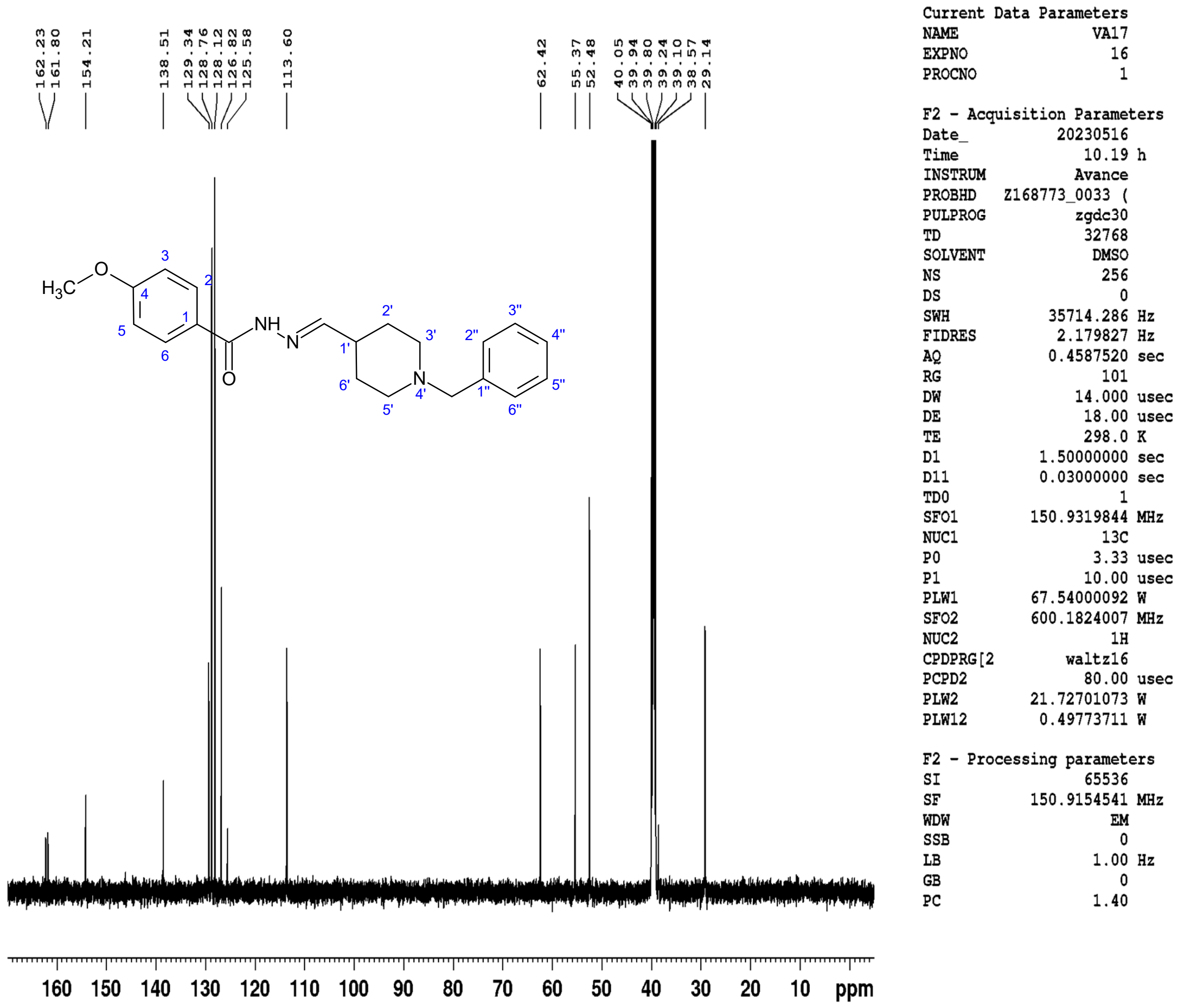

Figure S22. ^{13}C NMR spectrum of compound **3d** in DMSO-d_6

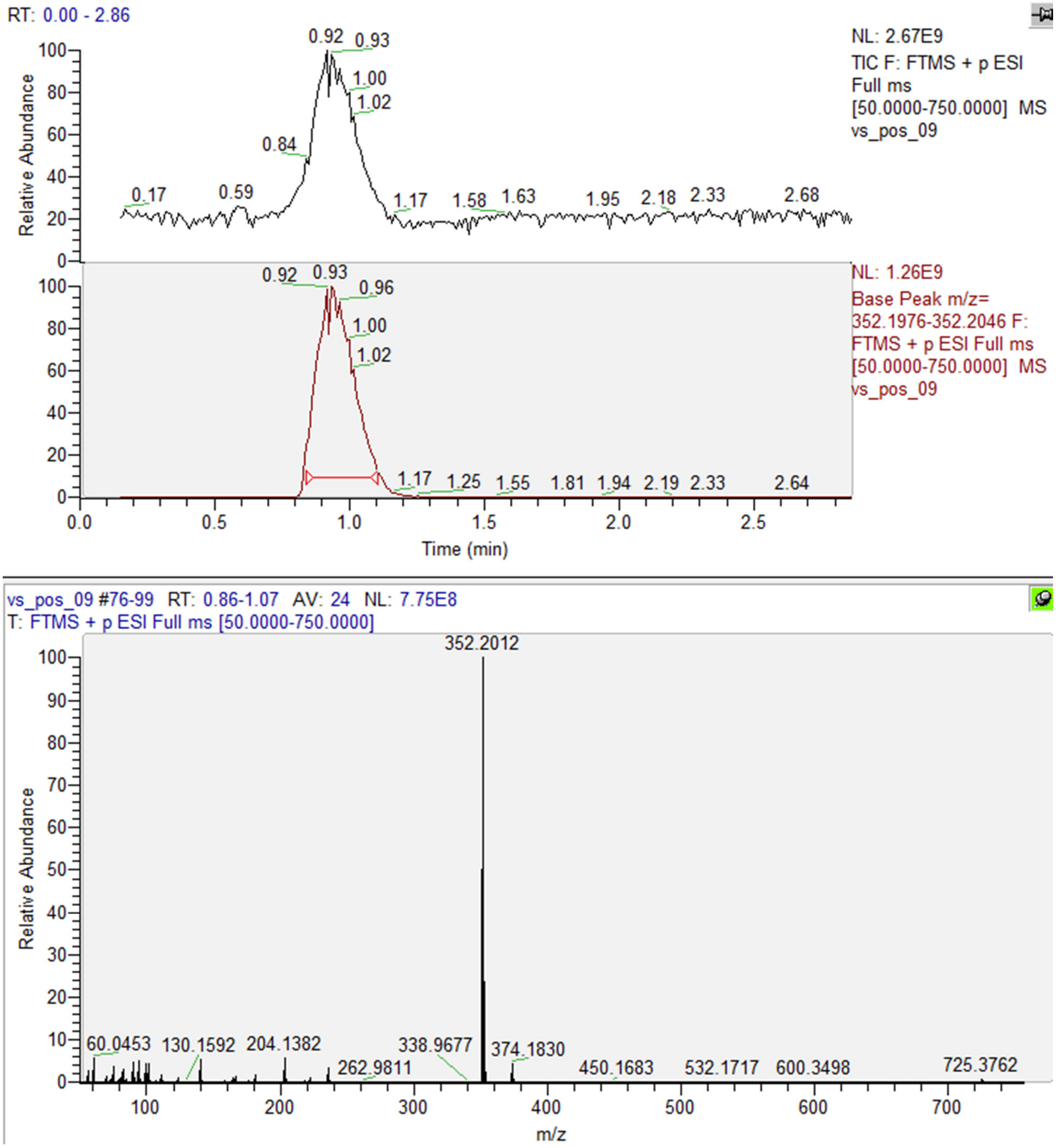

Figure S23. HRMS of compound **3d**

5. 1-benzyl-N'-[(E)-(4-hydroxyphenyl)methylidene]pyrrolidine-3-carbohydrazide, **3e**

Yield: 84%; m.p. 240-241◦C.^{1}H NMR (600 MHz, DMSO-d$_6$): 1:0.77 mixture of conformers; signals for major *synperiplanar* conformer around the amide bond: δ = 1.94-2.03 (m, 2H, H-4'), 2.40-2.42 (m, 2H, H-5'), 2.62-2.69 (m, 1H, 1/2H-2'), 2.88-2.91 (m, 1H, 1/2H-2'), 3.55-3.62 (m, 2H, CH$_2$), 3.61-3.66 (m, 1H, H-3'), 6.79 (d, J=8.6 Hz, 2H, H-3 and H-5), 7.22-7.26 (m, 1H, H-4''), 7.30-7.33 (m, 4H, H-2'', H-3'', H-5'' and H-6''), 7.44 (d, J=8.7 Hz, 2H, H-2 and H-6), 7.85 (s, 1H, CH), 9.84 (bs, 1H, OH), 11.05 (s, 1H, NH); resolved signals for minor *antiperiplanar* conformer around the amide bond: 8.04 (s, 1H, CH), 9.87 (bs, 1H, OH), 11.10 (s, 1H, NH).

^{13}C NMR (151 MHz, DMSO-d$_6$): signals for major *synperiplanar* conformer around the amide bond: δ = 26.95 (C-4'), 41.47 (C-3'), 53.58 (C-5'), 56.43 (C-2'), 59.30 (CH$_2$), 115.65 (C-3 and C-5), 125.37 (C-1), 126.75 (C-4''), 128.11 (C-2'' and C-6''), 128.28 (C-2 and C-6), 128.65 (C-3'' and C-5''), 139.16 (C-1''), 142.74 (CH), 158.99 (C-4), 175.01 (C=O); resolved signals for minor *antiperiplanar* conformer around the amide bond: 146.34 (CH), 159.22 (C-4), 169.78 (C=O). HRMS (ESI) m/z: calcd: [M+H]+ 324.170653. Found: [M+H]+ 324.1705

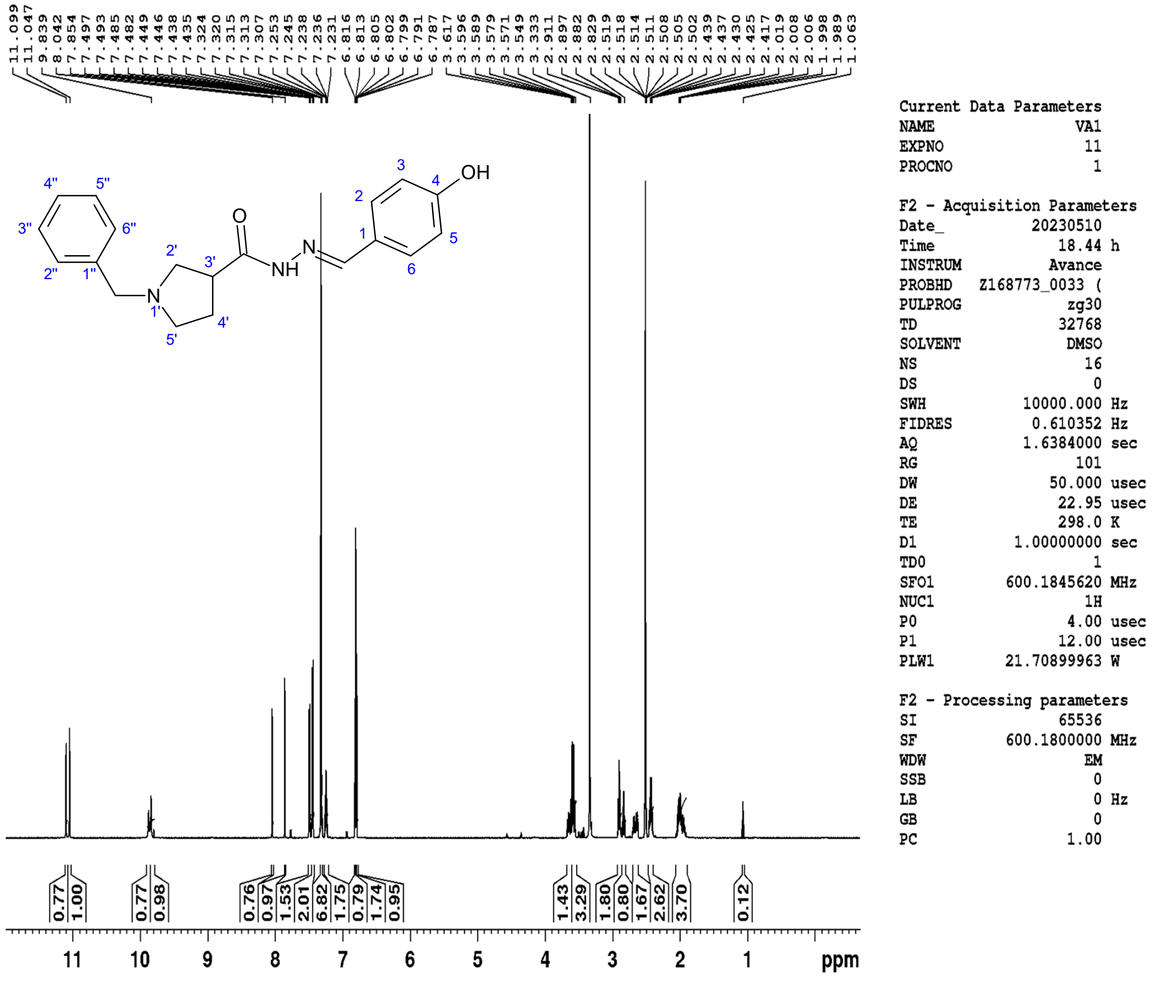

Figure S24. ^{1}H NMR spectrum of compound **3e** in DMSO-d_6

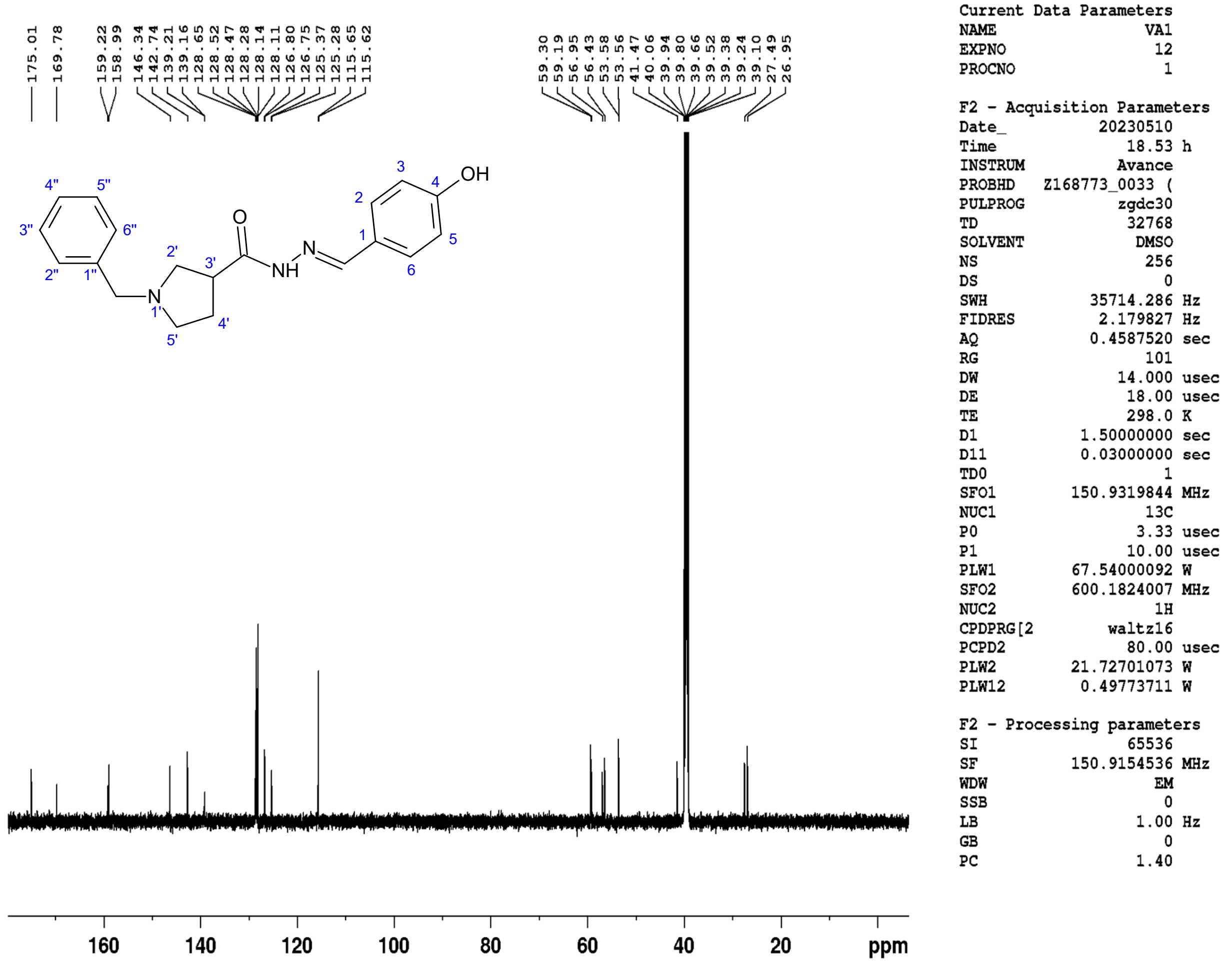

Figure S25. ^{13}C NMR spectrum of compound **3e** in DMSO-d_6

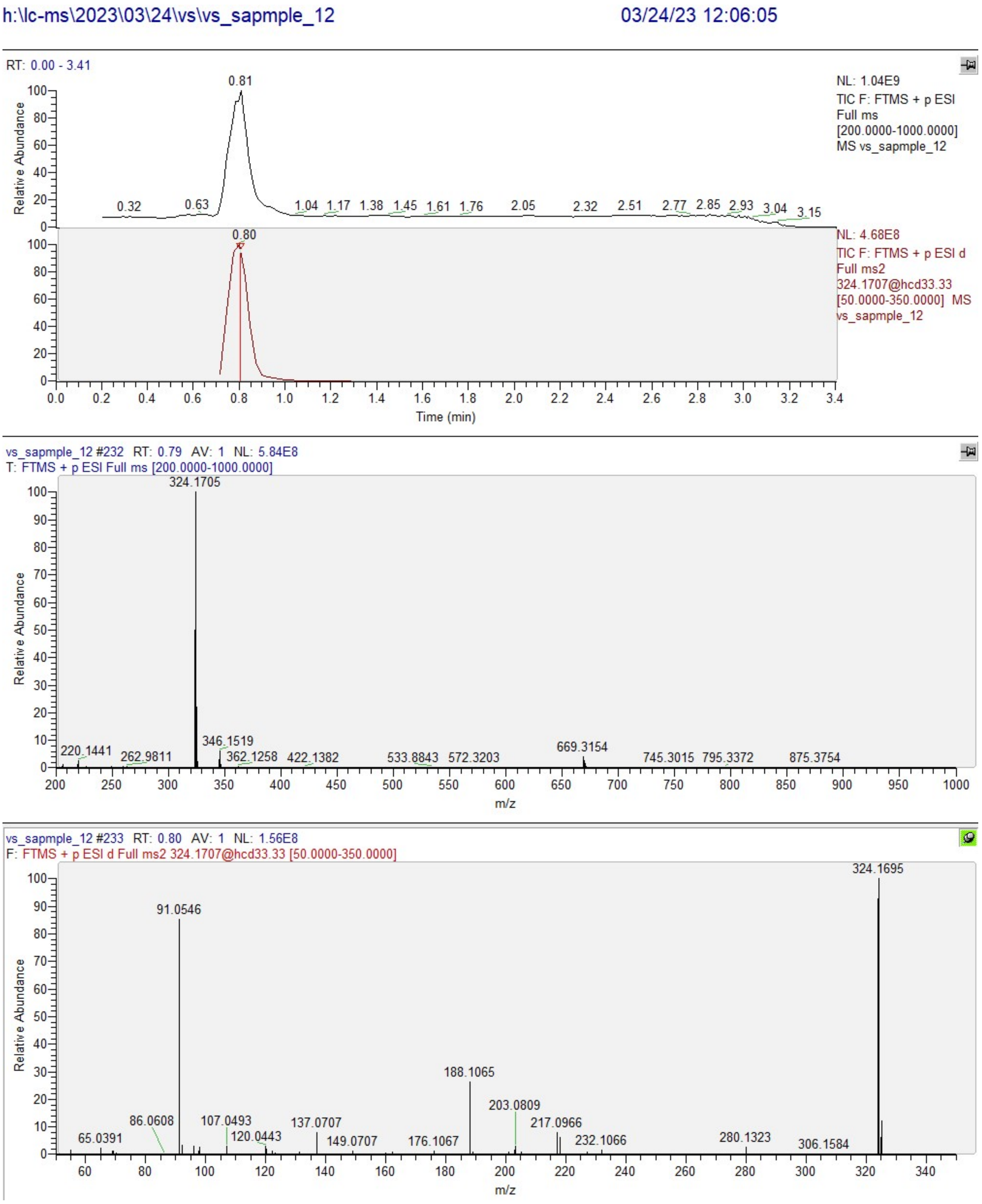

Figure S26. HRMS of compound **3e**

6. 1-benzyl-N'-[(E)-(2,4-dihydroxyphenyl)methylidene]pyrrolidine-3-carbohydrazide, 3f

Yield: 81%; m.p. 224-225◦C.^{1}H NMR (600 MHz, DMSO-d_6): δ = 1:0.39 mixture of conformers; signals for major *synperiplanar* conformer around the amide bond: δ = 1.92-2.04 (m, 2H, H-4'), 2.41-2.48 (m, 2H, H-5'), 2.66-2.69 (m, 1H, 1/2H-2'), 2.82-2.94 (m, 1H, 1/2H-2'), 3.52-3.57 (m, 1H, H-3'), 3.57-3.61 (m, 2H, CH_2), 6.29 (d, J=2.3 Hz, 1H, H-3), 6.33 (dd, J=8.4, 2.3 Hz, 1H, H-5), 7.25 (d, J=8.5 Hz, 1H, H-6), 7.29-7.33 (m, 5H, Ar), 8.21 (s, 1H, CH), 9.91 (bs, 1H, OH), 11.32 (s, 1H, OH), 11.36 (s, 1H, NH); resolved signals for minor *antiperiplanar* conformer around the amide bond: 8.11 (s, 1H, CH), 9.79 (bs, 1H, OH), 10.11 (bs, 1H, OH), 11.04 (s, 1H, NH).

^{13}C NMR (151 MHz, DMSO-d_6): signals for major *synperiplanar* conformer around the amide bond: δ = 27.47 (C-4'), 41.23 (C-3'), 53.52 (C-5'), 56.83 (C-2'), 59.17 (CH_2), 102.58 (C-3), 107.57 (C-5), 110.41 (C-1), 126.81 (C-4''), 128.14 (C-2'' and C-6''), 128.51 (C-3'' and C-5''), 131.16 (C-6), 139.17 (C-1''), 147.58 (CH), 159.27 (C-2), 160.52 (C-4), 169.58 (C=O); resolved signals for minor *antiperiplanar* conformer around the amide bond: 141.95 (CH), 157.97 (C-2), 160.22 (C-4), 174.44 (C=O).HRMS (ESI) m/z: calcd: [M+H]+ 340.165568. Found: [M+H]+ 340.1655

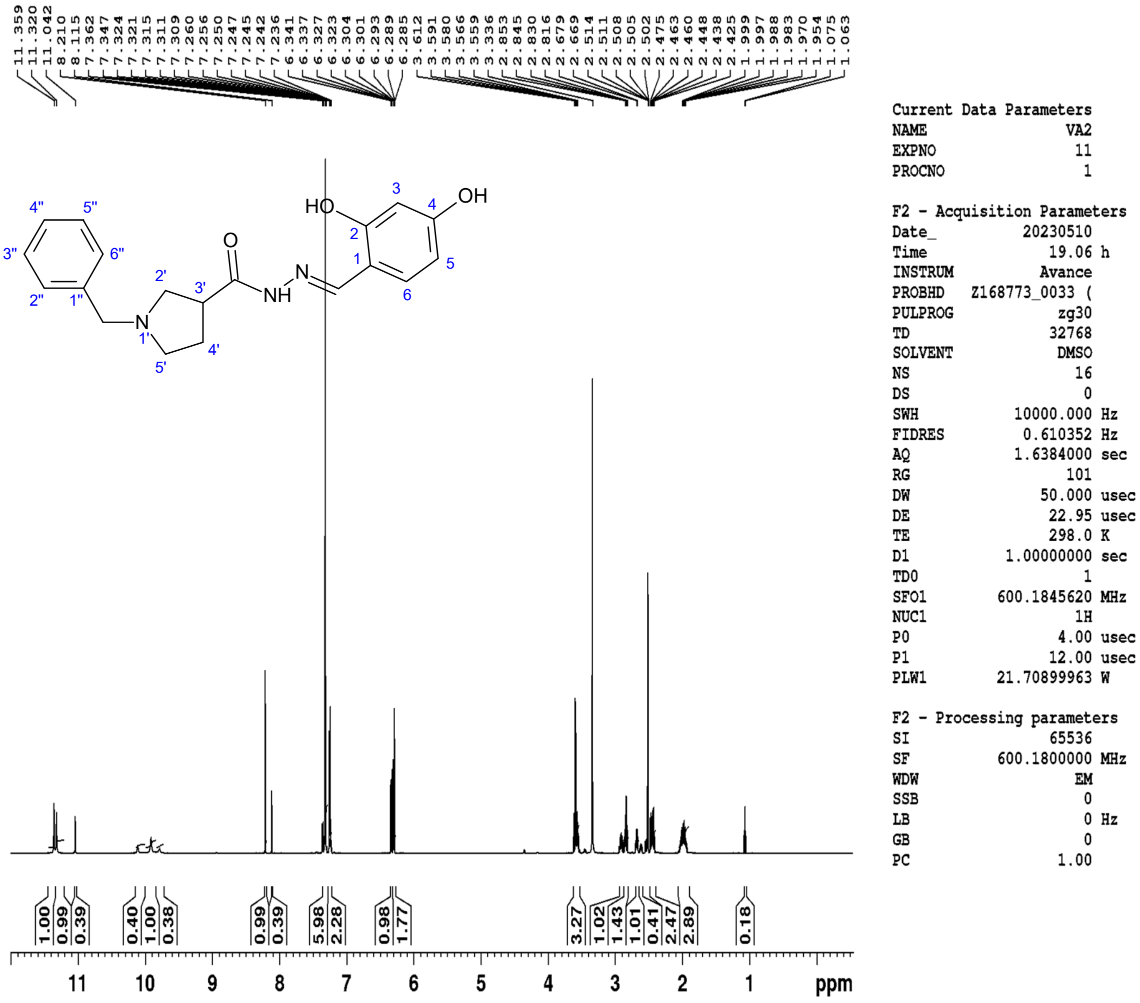

Figure S27. ^{1}H NMR spectrum of compound **3f** in DMSO-d_6

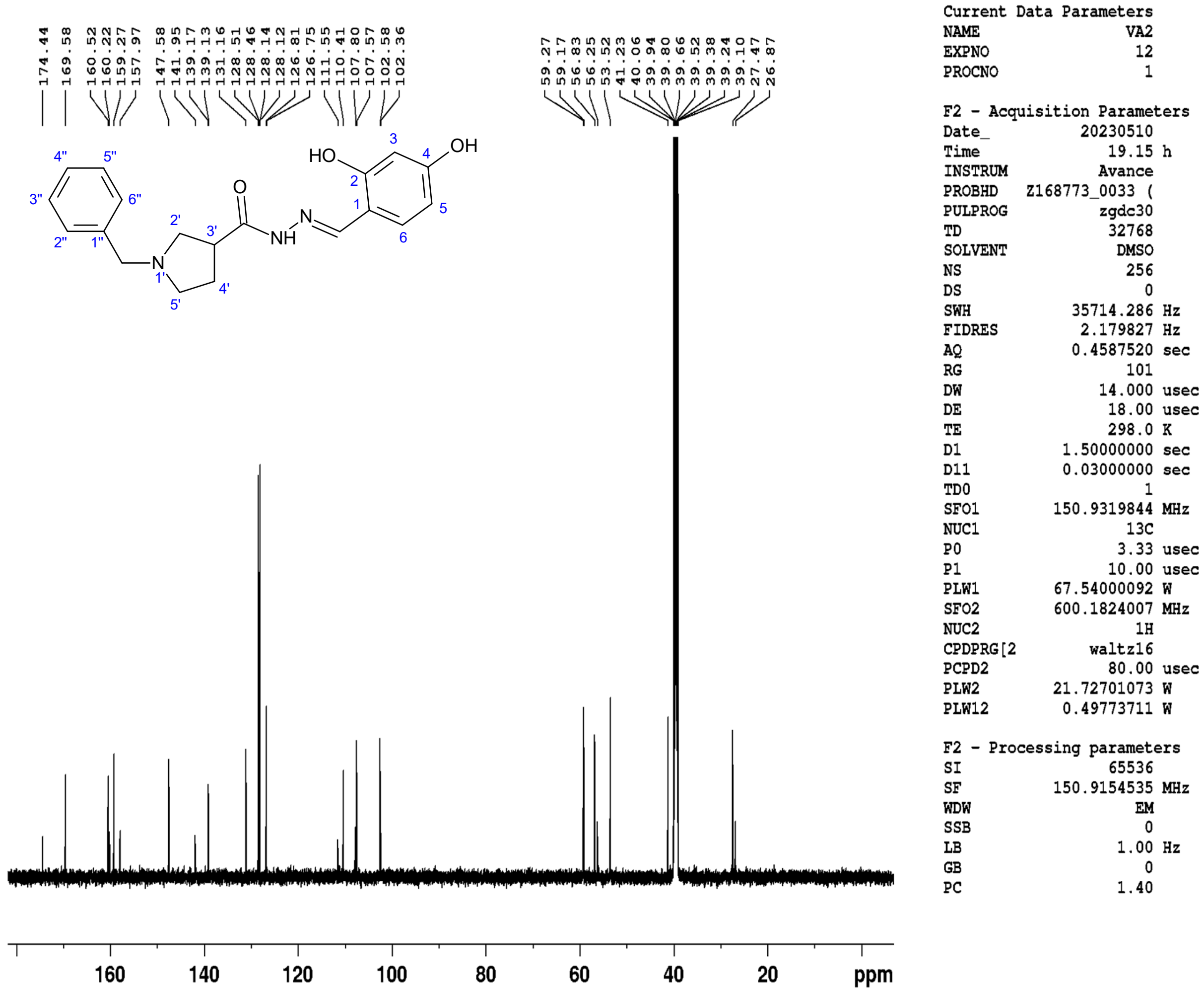

Figure S28. ^{13}C NMR spectrum of compound **3f** in DMSO-d_6

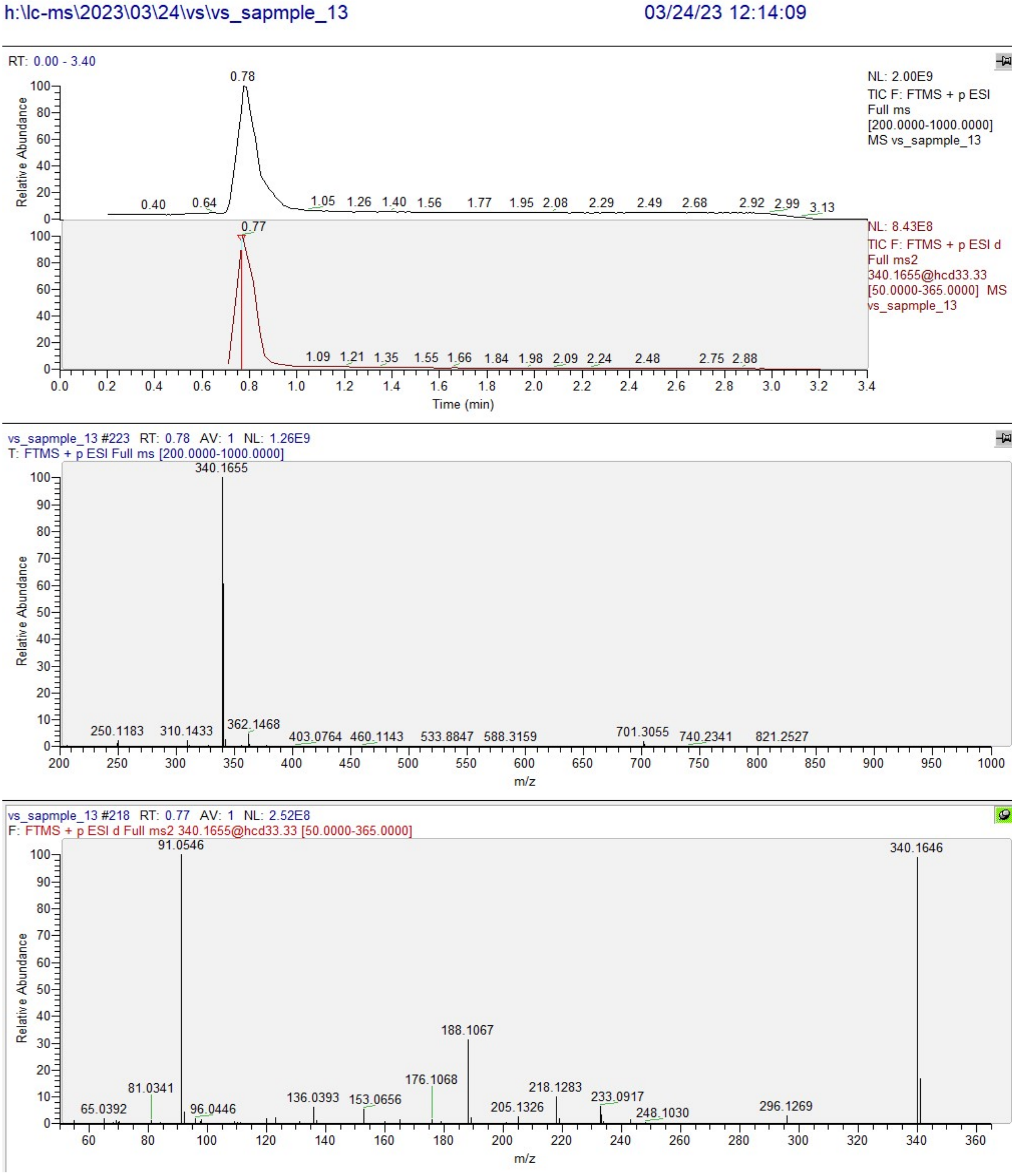

Figure S29. HRMS of compound **3f**

7. 1-benzyl-N'-[(E)-(3,4-dihydroxyphenyl)methylidene]pyrrolidine-3-carbohydrazide, **3g**

Yield: 72%; m.p. 199-201◦C.^{1}H NMR (600 MHz, DMSO-d_6): 1:0.65 mixture of conformers; signals for major *synperiplanar* conformer around the amide bond: δ = 2.57 (m, J=8.3 Hz, 2H, H-4'), 2.63 (d, J=8.45 Hz, 2H, H-5'), 3.32-3.37 (m, 1H, H-2'), 3.57 (t, J=9.5 Hz, 1H, H-2'), 3.91 (dq, J=6.2, 8.6 Hz, 1H, H-3'), 4.35 (d, J=15.0 Hz, 1H) and 4.46 (d, J=15.0 Hz, 1H, CH_2), 6.75 (dd, J=0.6, 7.7 Hz, 1H, H-5), 6.89 (dd, J=1.2, 8.3 Hz, 1H, H-6), 7.09 (s, 1H, H-2), 7.23-7.25 (m, 2H, H-2" and H-6"), 7.27-7.30 (m, 1H, H-4"), 7.34-7.37 (m, 2H, H-3" and H-5"), 7.80 (s, 1H, CH), 9.15 (bs, 1H, OH), 9.39 (bs, 1H, OH), 11.23 (s, 1H, NH); resolved signals for minor *antiperiplanar* conformer around the amide bond: 7.96 (s, 1H, CH), 9.24 (bs, 1H, OH), 9.37 (bs, 1H, OH), 11.26 (s, 1H, NH).

^{13}C NMR (151 MHz, DMSO-d_6): signals for major *synperiplanar* conformer around the amide bond: δ = 33.54 (C-3'), 35.41 (C-4'), 45.79 (CH_2), 48.98 (C-5'), 49.37 (C-2'), 113.21 (C-2), 116.05 (C-5), 120.37 (C-6), 126.02 (C-1), 127.73 (C-4"), 128.05 (C-2" and C-6"), 129.06 (C-3" and C-5"), 137.24 (C-1"), 144.49 (CH), 146.11 (C-3), 148.17 (C-4), 172.83 (C=O); resolved signals for minor *antiperiplanar* conformer around the amide bond: 147.81 (CH), 146.15 (C-3), 148.43 (C-4), 173.81 (C=O). HRMS (ESI) m/z: calcd: [M+H]+ 340.165568. Found: [M+H]+ 340.1646

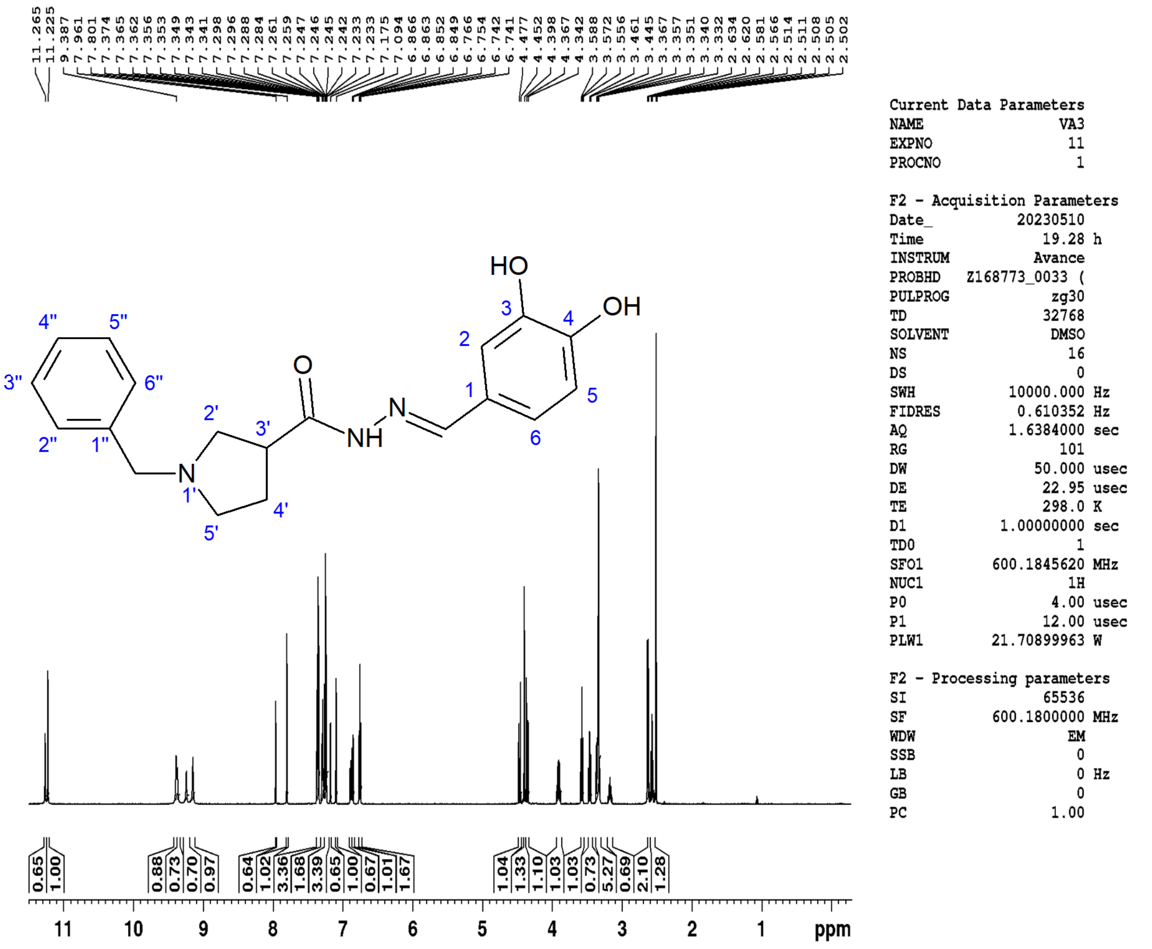

Figure S30. ^{1}H NMR spectrum of compound **3g** in DMSO-d_6

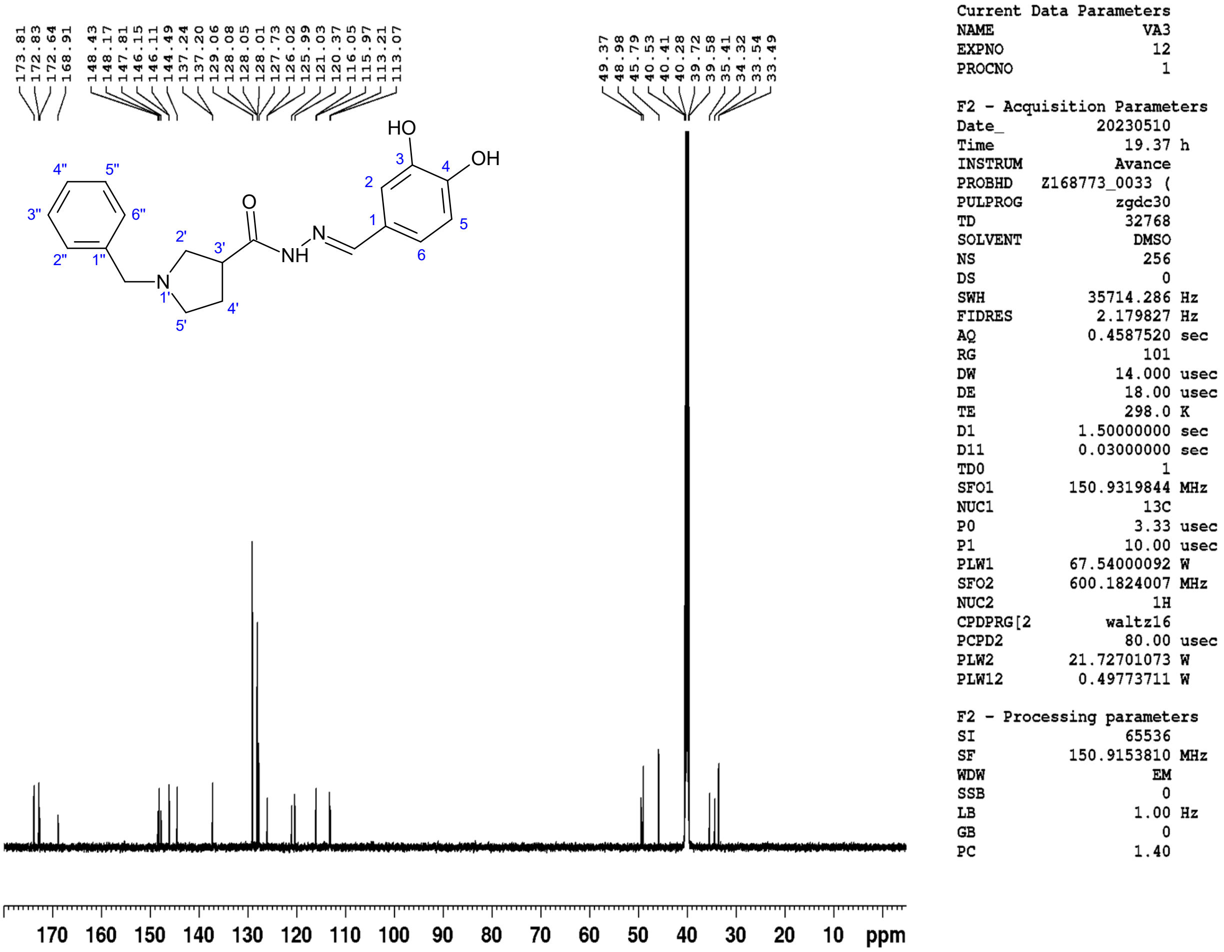

Figure S31. ^{13}C NMR spectrum of compound **3g** in DMSO-d_6

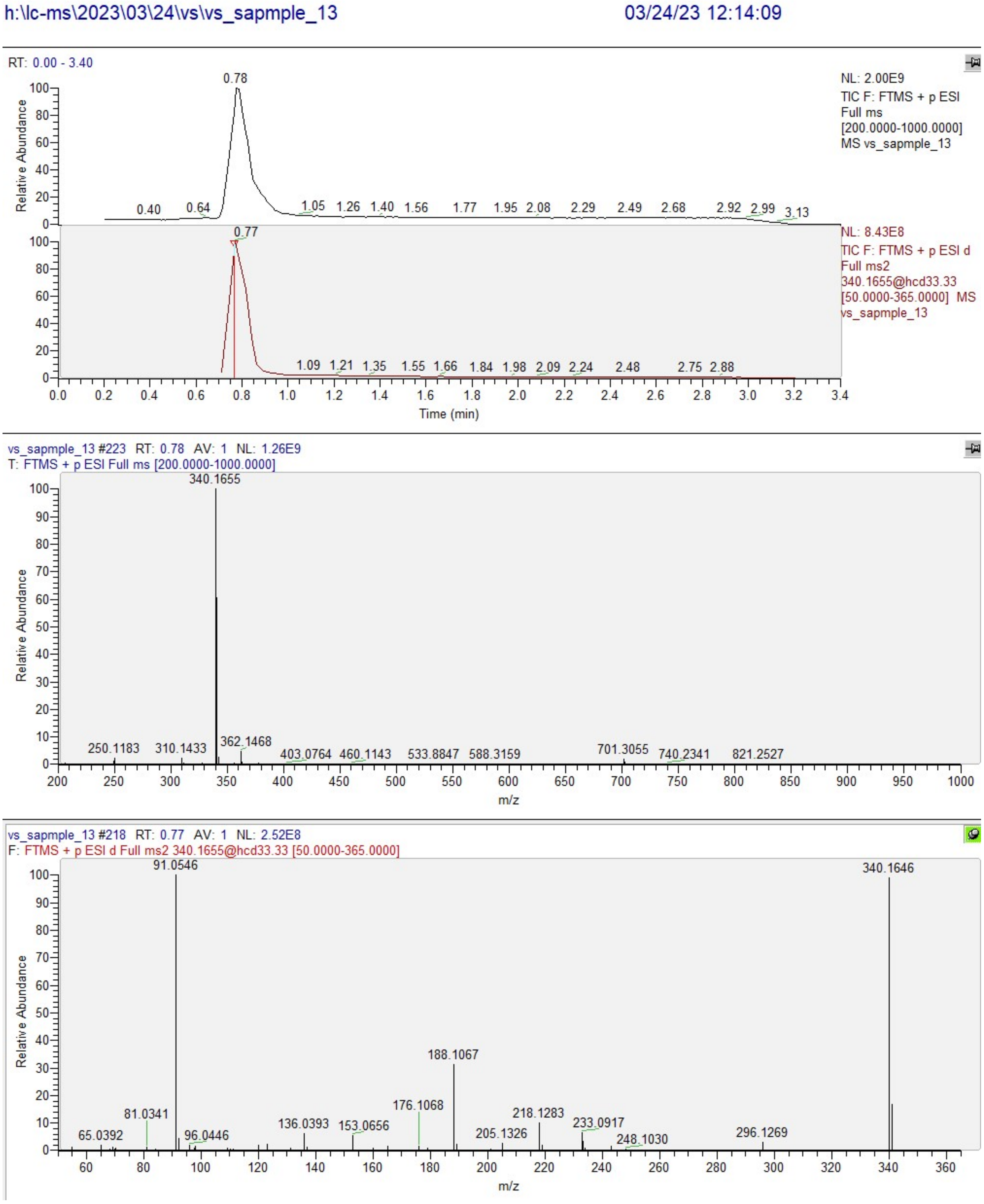

Figure S32. HRMS of compound **3g**

8. 1-benzyl-N'-[(E)-(2,4,6-trihydroxyphenyl)methylidene]pyrrolidine-3-carbohydrazide, **3h**

Yield: 65%; m.p. 215-217◦C.^{1}H NMR (600 MHz, DMSO-d$_6$): 1:0.19 mixture of conformers; signals for major *synperiplanar* conformer around the amide bond: δ = 1.91-2.03 (m, 2H, H-4'), 2.41-2.47 (m, 2H, H-5'), 2.66-2.69 (m, 1H, H-2'), 2.81-2.85 (m, 1H, H-2'), 2.85-2.91 (m, 1H, H-3'), 3.57 (d, J=12.8 Hz, 1H) and 3.60 (d, J=13.0 Hz, 1H, CH$_2$), 5.81 (s, 2H, H-3 and H-5), 7.23-7.27 (m, 1H, H-4''), 7.30-7.33 (m, 4H, H-2'', H-3'', H-5'' and H-6''), 8.49 (s, 1H, CH), 9.77 (bs, 1H, OH), 10.95 (bs, 2H, OH), 11.33 (s, 1H, NH); resolved signals for minor *antiperiplanar* conformer around the amide bond: 8.37 (s, 1H, CH), 9.80 (bs, 1H, OH), 10.51 (bs, 2H, OH), 11.12 (s, 1H, NH).

^{13}C NMR (151 MHz, DMSO-d$_6$): signals for major *synperiplanar* conformer around the amide bond: δ = 27.47 (C-3'), 41.19 (CH$_2$), 53.50 (C-4'), 56.81 (C-5'), 59.16 (C-2'), 94.29 (C-3 and C-5), 98.80 (C-1), 126.81 (C-4''), 128.14 (C-2'' and C-6''), 128.51 (C-3'' and C-5''), 139.12 (C-1''), 145.15 (CH), 159.48 (C-2 and C-6), 161.34 (C-4), 169.21 (C=O). HRMS (ESI) m/z: calcd: [M+H]+ 356.160483. Found: [M+H]+ 356.1604

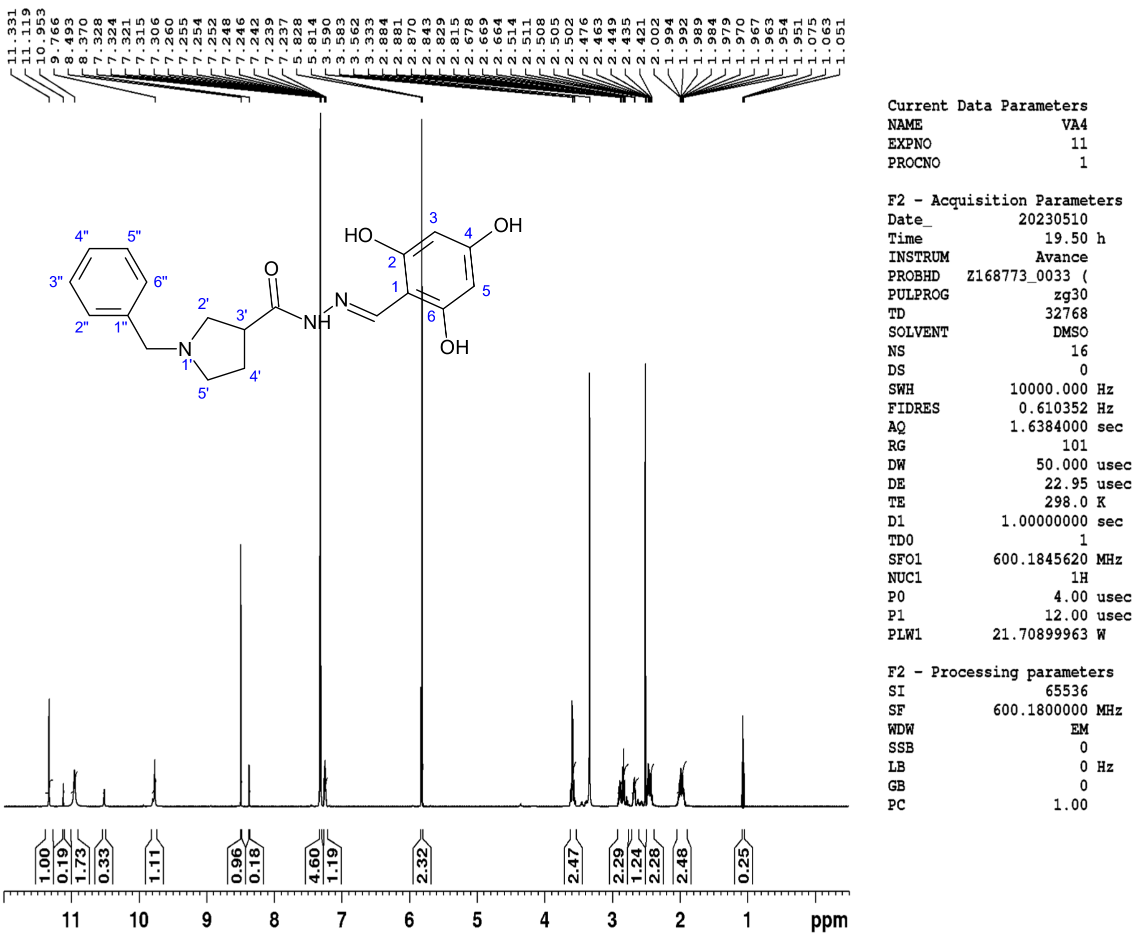

Figure S33. ^{1}H NMR spectrum of compound **3h** in DMSO-*d*$_6$

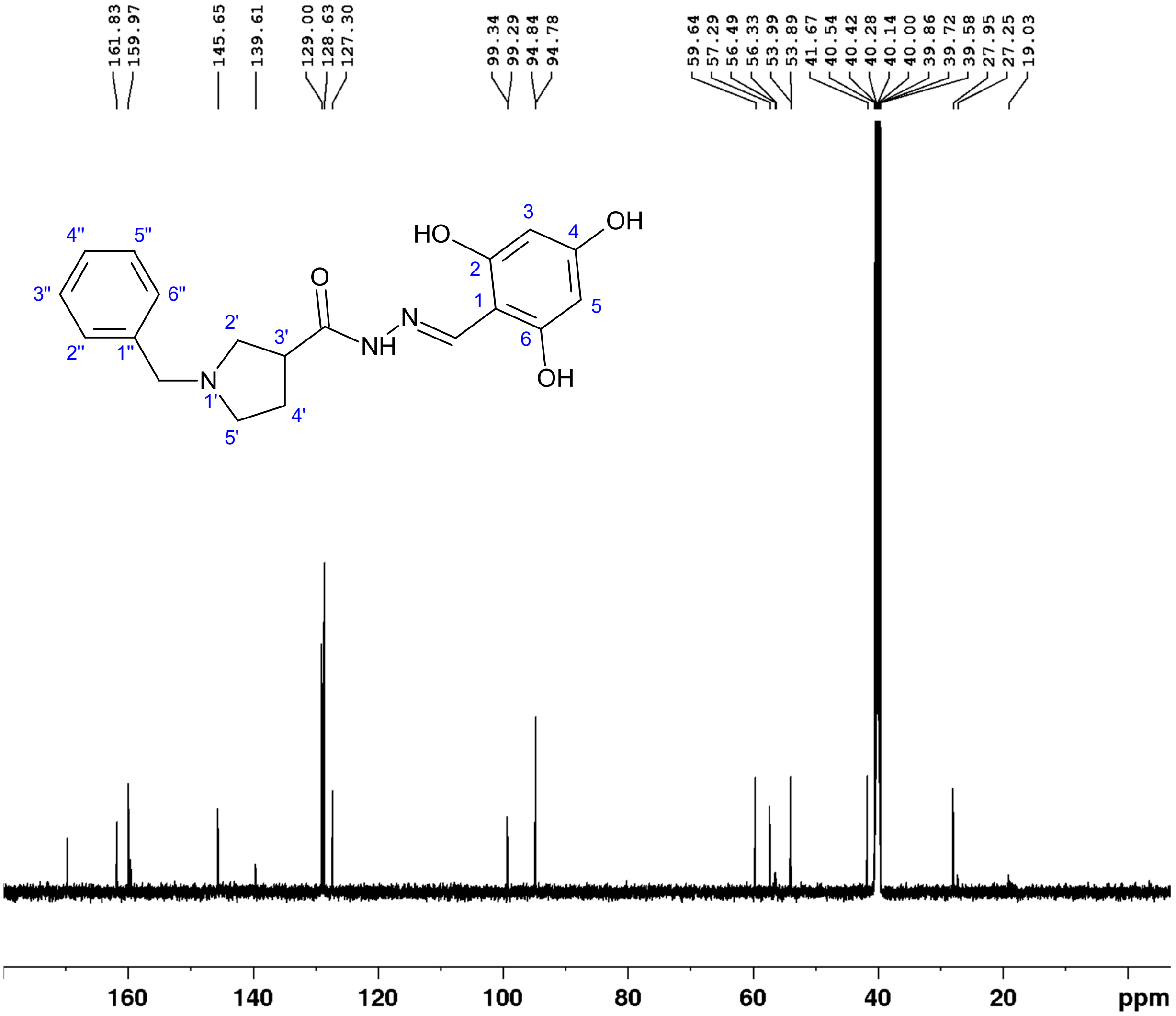

Figure S34. ^{13}C NMR spectrum of compound **3h** in DMSO-d_6

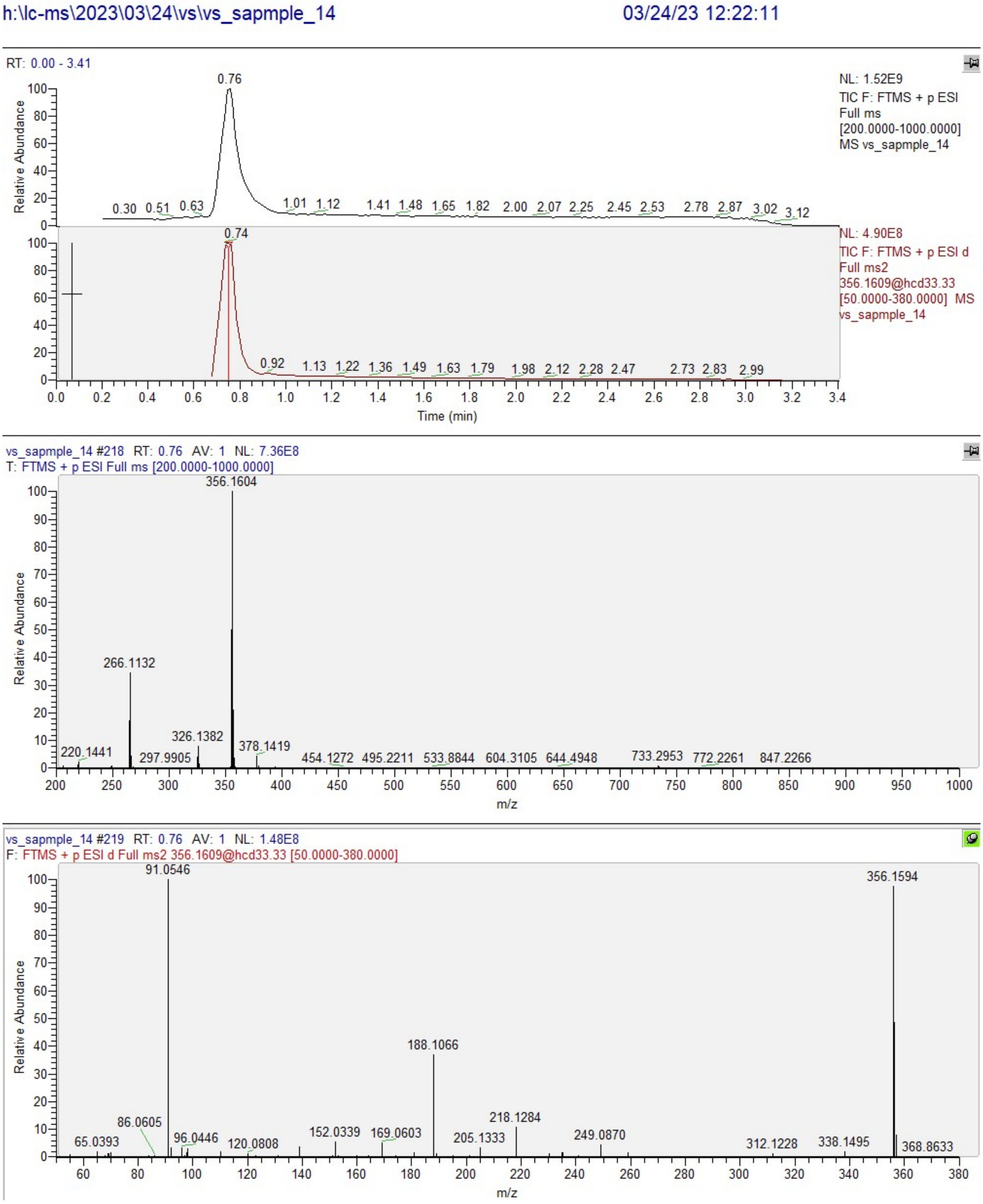

Figure S35. HRMS of compound **3h**

9. ***tert-butyl (2-{(2E)-2-[(1-benzyl-1H-indol-3-yl)methylidene]hydrazinyl}-2-oxoethyl)carbamate, 3i*** ^{1}H NMR, ^{13}C NMR and HRMS spectra of compound **3i** can be found in https://doi.org/10.1007/s00044-019-02293-w

10. tert-butyl (2-{(2E)-2-[(5-methoxy-1-methyl-1H-indol-3-yl)methylidene]hydrazinyl}-2-oxoethyl)carbamate, **3j**

Yield: 48%; m.p. 203-206◦C.^{1}H NMR (600 MHz, DMSO-d6): 1:0.28 mixture of conformers; signals for major *synperiplanar* conformer around the amide bond: δ = 1.41 (s, 9H, CH_3), 3.79 (s, 3H, OCH_3), 4.16 (d, J=6.0 Hz, 2H, CH_2), 6.76 (t, J=5.9 Hz, 1H, H-4), 6.85 (dd, J=2.6, 8.8 Hz, 1H, H-6), 7.34 (d, J=8.8 Hz, 1H, H-7), 7.62 (d, J=2.4 Hz, 1H, H-4), 7.72 (d, J=2.9 Hz, 1H, C-2), 8.14 (s, 1H, CH), 11.00 (s, 1H, NH), 11.40 (bs, 1H, NH); resolved signals for minor *antiperiplanar* conformer around the amide bond: 8.35 (s, 1H, CH), 11.09 (s, 1H, NH), 11.40 (bs, 1H, NH).

^{13}C NMR (151 MHz, DMSO-d6): signals for major *synperiplanar* conformer around the amide bond: δ = 28.22 (CH_3), 41.39 (CH_2), 55.05 (OCH_3), 77.88 (OC), 103.48 (C-4), 111.10 (C-3), 112.16 (C-7), 112.53 (C-6), 124.63 (C-3a), 130.50 (C-2), 131.93 (C-7a), 140.68 (CH), 154.37 (C-5), 155.89 (OC=O), 169.74 (C=O).HRMS (ESI) m/z: calcd: [M+H]+ 347.171382. Found: [M+H]+ 347.1712

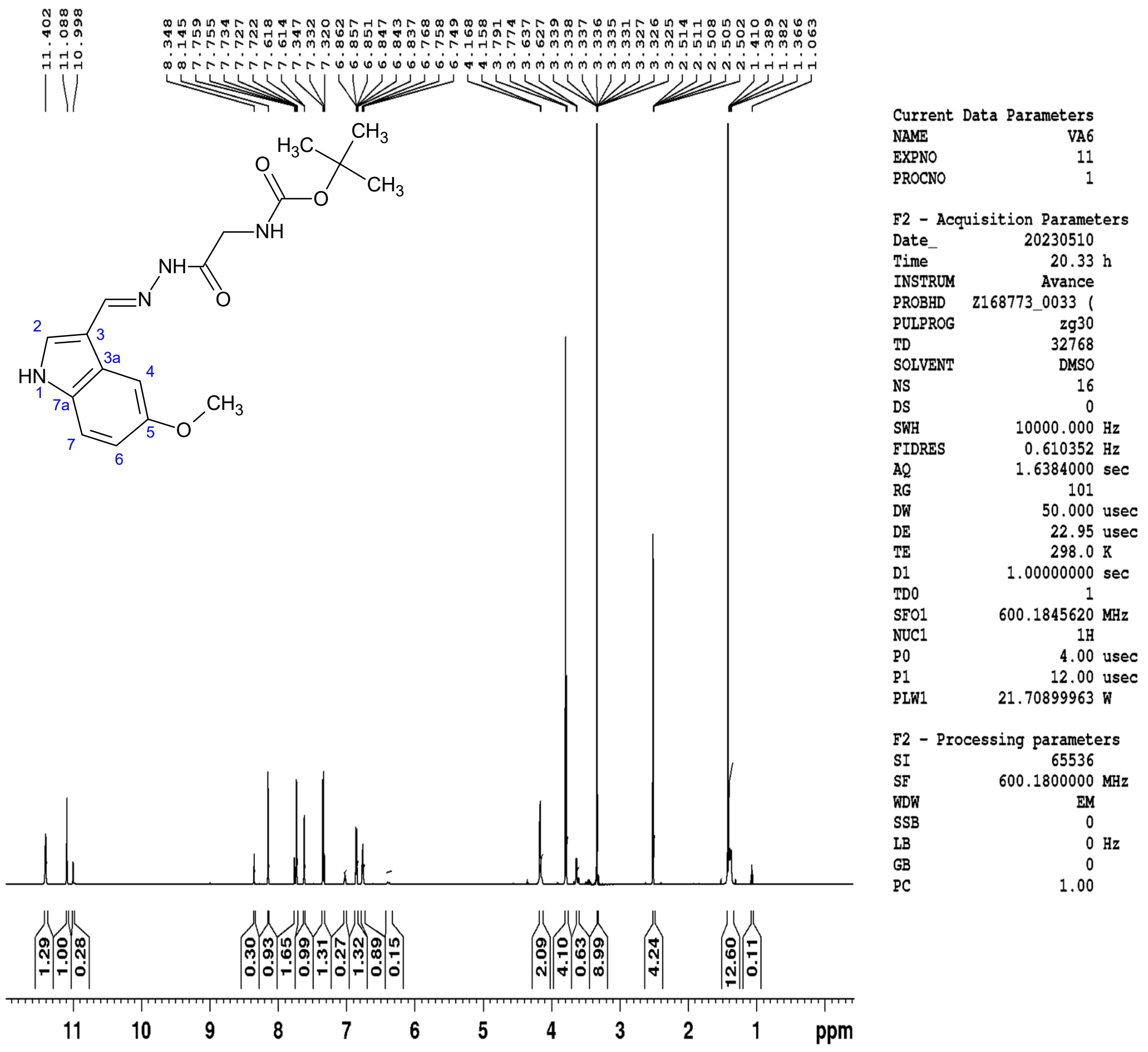

Figure S36. ^{1}H NMR spectrum of compound **3j** in DMSO-d_6

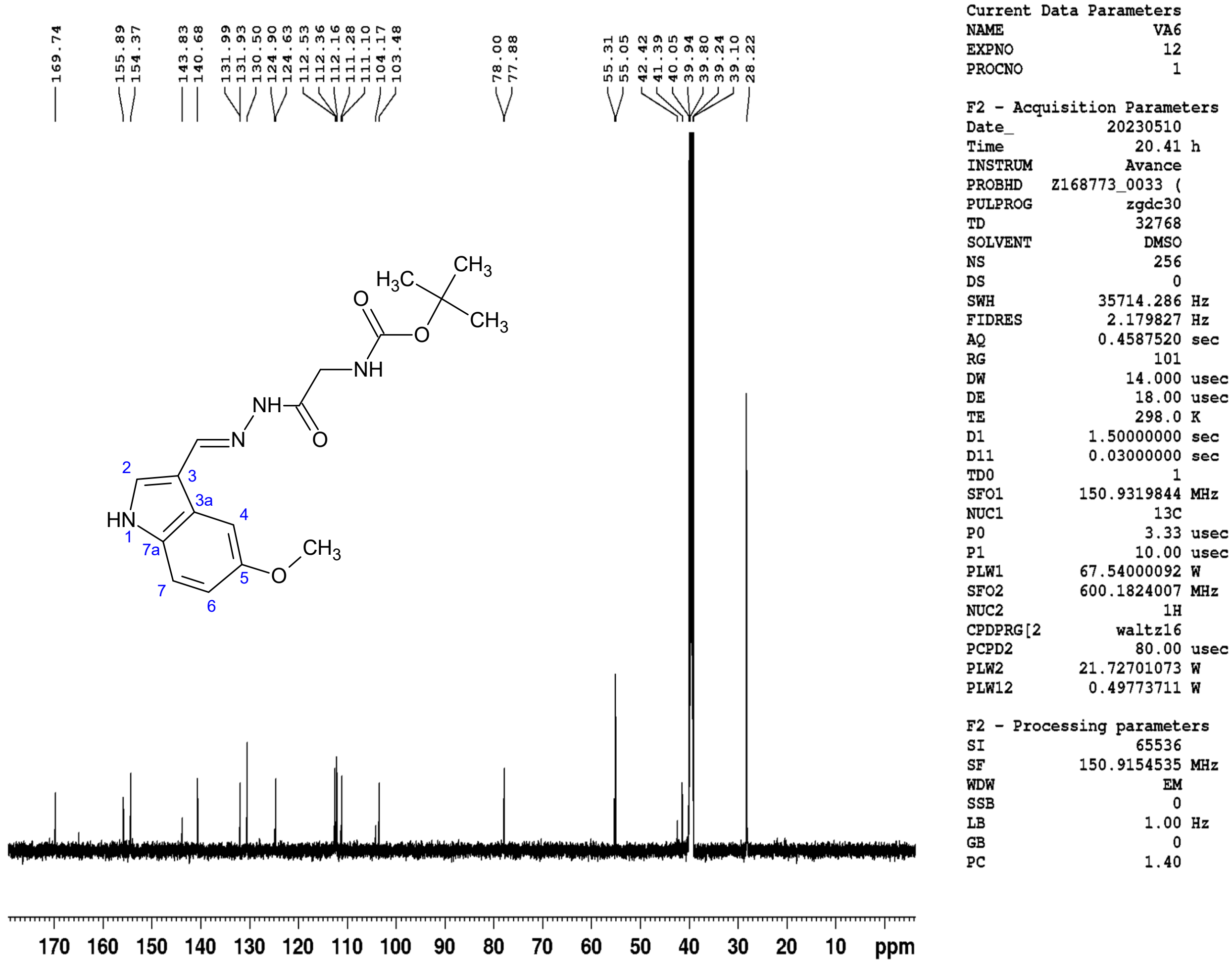

Figure S37. ^{13}C NMR spectrum of compound **3j** in DMSO-d_6

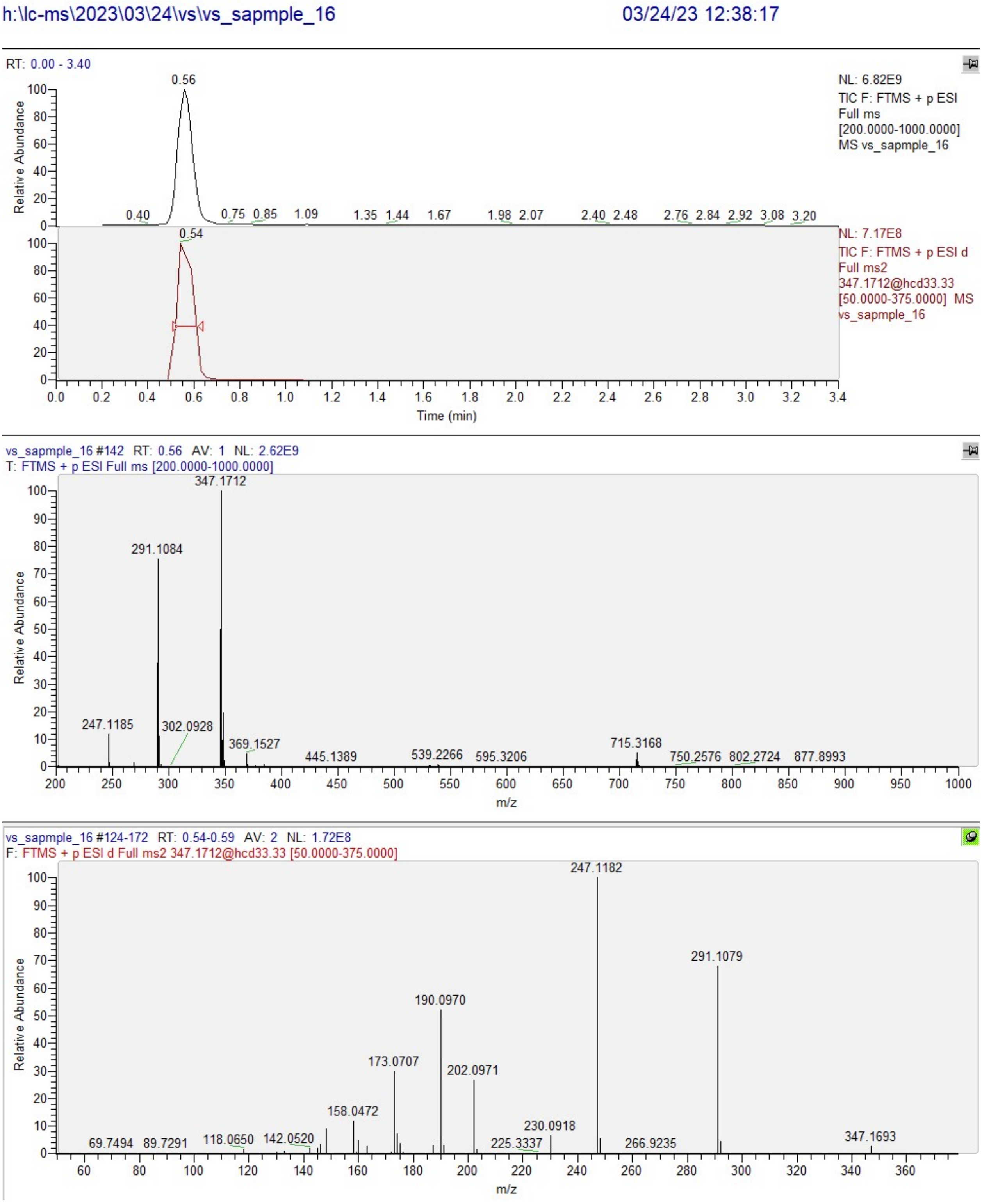

Figure S38. HRMS of compound **3j**

11. tert-butyl (2-{(2E)-2-[(5-methoxy-1H-indol-3-yl)methylidene]hydrazinyl}-2-oxoethyl)carbamate, 3k

Yield: 83%; m.p. 197-199◦C.^{1}H NMR (600 MHz, DMSO-d$_6$): 1:0.33 mixture of conformers; signals for major *synperiplanar* conformer around the amide bond: δ = 1.41 (s, 9H, CH_3), 3.78 (s, 3H, NCH_3), 3.80 (s, 3H, OCH_3), 4.16 (d, J=5.9 Hz, 2H, CH_2), 6.76 (t, J=5.9 Hz, 1H, NH), 6.92 (dd, J=2.6, 8.8 Hz, 1H, H-6), 7.41 (d, J=8.8 Hz, 1H, H-7), 7.62 (d, J=2.5 Hz, 1H, H-4), 7.71 (s, 1H, C-2), 8.11 (s, 1H, CH), 11.07 (s, 1H, NH); resolved signals for minor *antiperiplanar* conformer around the amide bond: 8.32 (s, 1H, CH), 11.00 (1H, s),

^{13}C NMR (151 MHz, DMSO-d$_6$): signals for major *synperiplanar* conformer around the amide bond: δ = 28.01 (CH_3), 32.91 (NCH_3), 41.38 (CH_2), 55.11 (OCH_3), 77.88 (OC), 103.66 (C-4), 109.95 (C-3), 111.05 (C-7), 112.10 (C-6), 125.01 (C-3a), 132.65 (C-7a), 134.03 (C-2), 140.20 (CH), 154.65 (C-5), 155.89 (OC=O), 169.72 (C=O).HRMS (ESI) m/z: calcd: [M+H]+ 361.187032. Found: [M+H]+ 361.1869

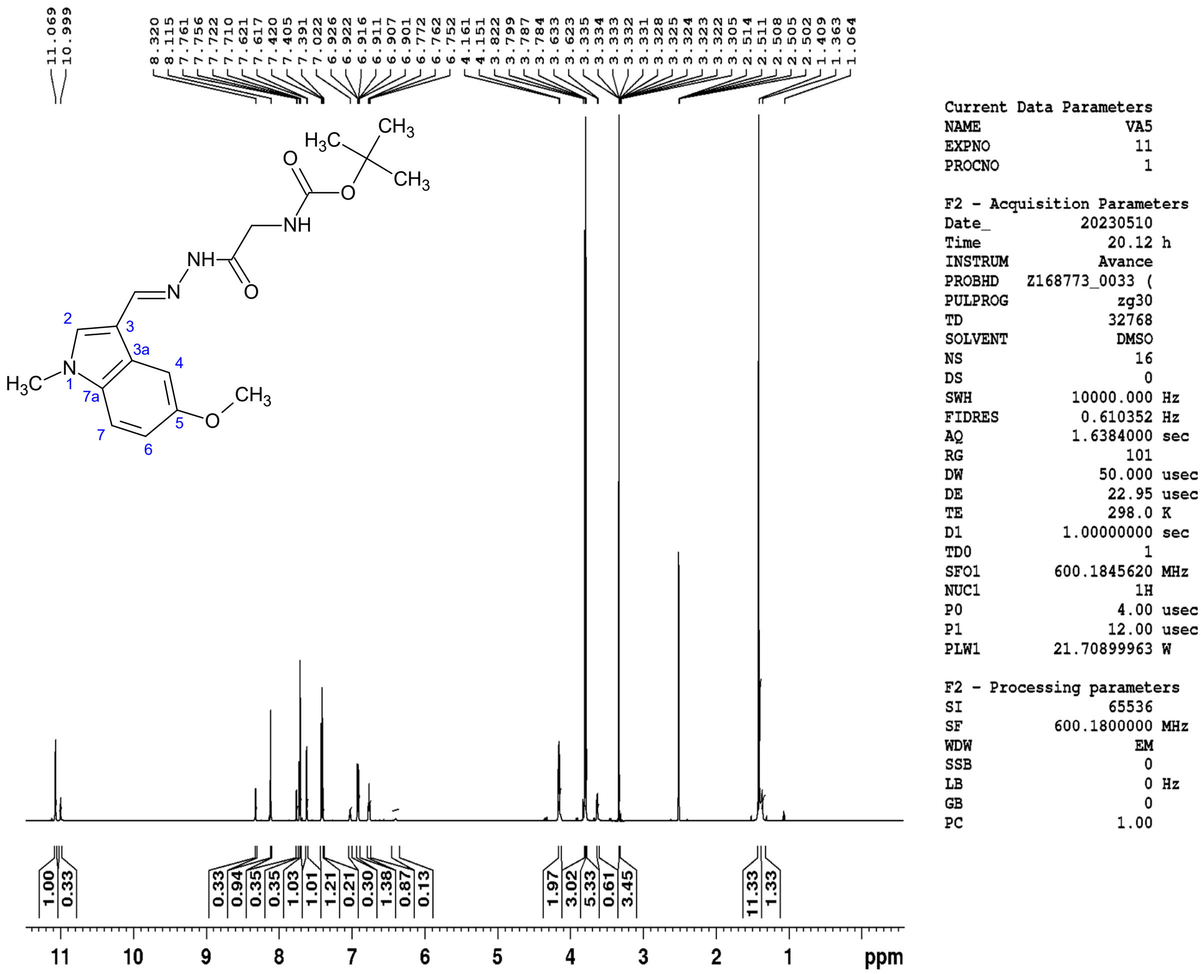

Figure S39. ^{1}H NMR spectrum of compound **3k** in DMSO-*d$_6$*

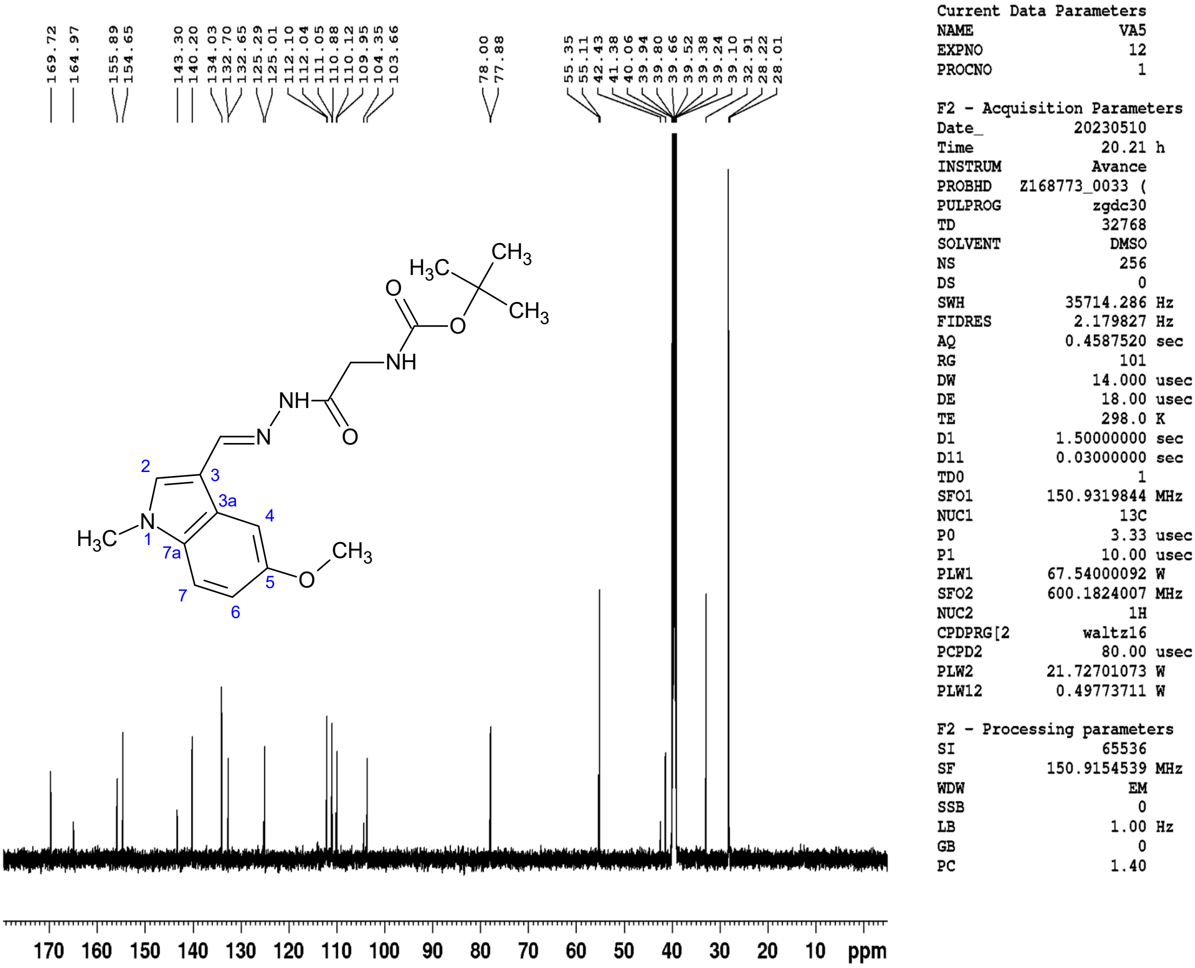

Figure S40. ^{13}C NMR spectrum of compound **3k** in DMSO-d_6

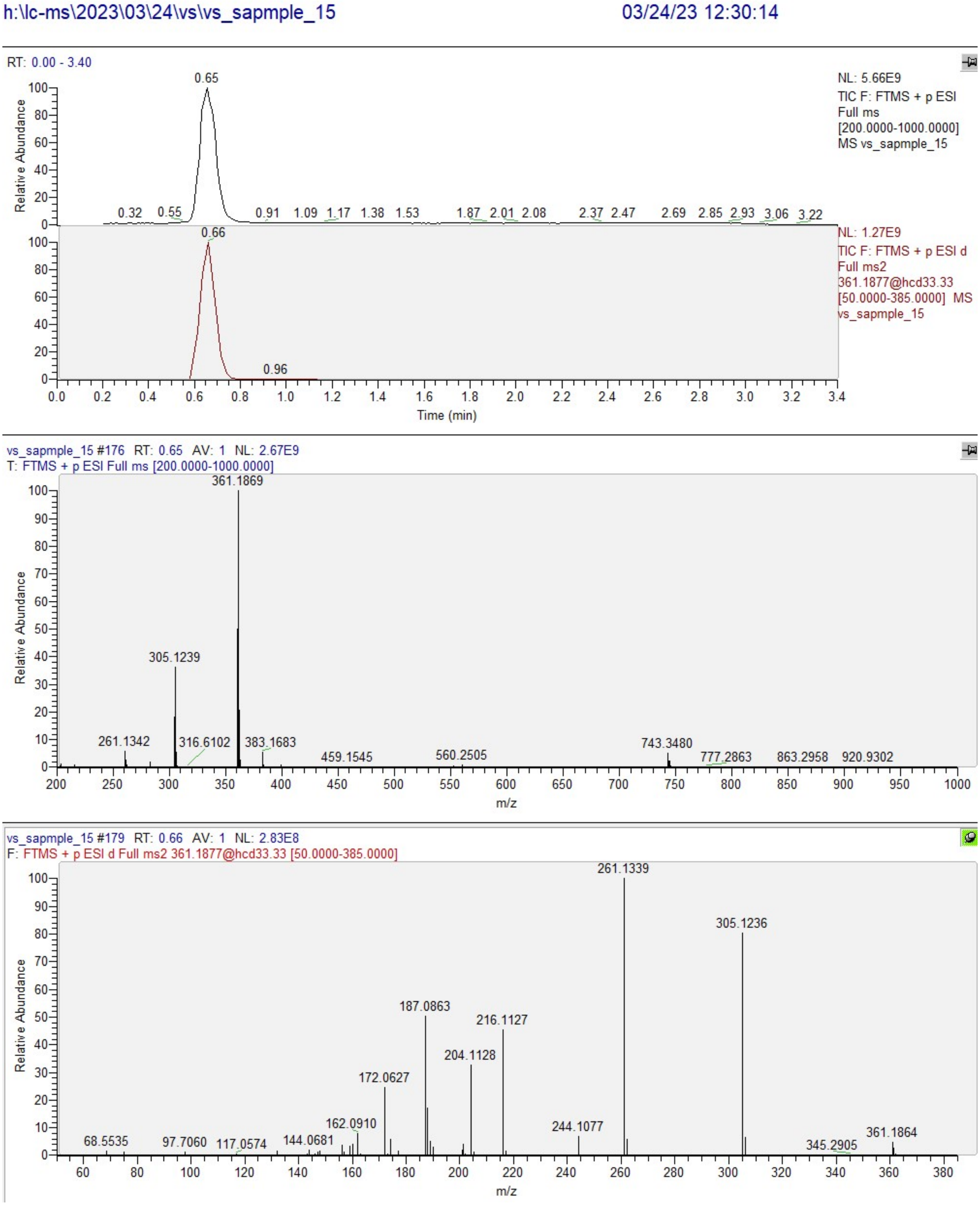

Figure S41. HRMS of compound **3k**

12.. *tert-butyl (2-{(2E)-2-[(1-methyl-1H-indol-3-yl)methylidene]hydrazinyl}-2-oxoethyl)carbamate,* **3l**

Yield: 54%; m.p. 178-179◦C.^{1}H NMR (600 MHz, DMSO-d6): 1:0.40 mixture of conformers; signals for major *synperiplanar* conformer around the amide bond: δ = 1.41 (s, 9H, CH_3), 3.82 (s, 3H, NCH_3), 4.12 (d, J=6.1 Hz, 2H, CH_2), 6.83 (t, J=6.1 Hz, 1H, NH), 7.22 (ddd, J=0.7, 7.1, 7.8 Hz, 1H, H-5), 7.28 (ddd, J=1.2, 7.0, 8.2 Hz, 1H, H-6), 7.51 (d, J=7.5 Hz, 1H, H-7), 7.77 (s, 1H, C-2), 8.08 (d, J=7.8 Hz, 1H, H-4), 8.13 (s, 1H, CH), 11.06 (s, 1H, NH); resolved signals for minor *antiperiplanar* conformer around the amide bond: 8.34 (s, 1H, CH), 11.02 (s, 1H, NH).

^{13}C NMR (151 MHz, DMSO-d6): signals for major *synperiplanar* conformer around the amide bond: δ = 28.24 (CH_3), 32.76 (NCH_3), 41.42 (CH_2), 77.87 (OC), 110.27 (C-7), 110.38 (C-3), 120.83 (C-5), 121.55 (C-4), 122.66 (C-6), 124.43 (C-3a), 133.90 (C-2), 137.56 (C-7a), 140.22 (CH), 155.96 (OC=O), 169.78 (C=O).HRMS (ESI) m/z: calcd: [M+H]+ 331.176467. Found: [M+H]+ 331.1764

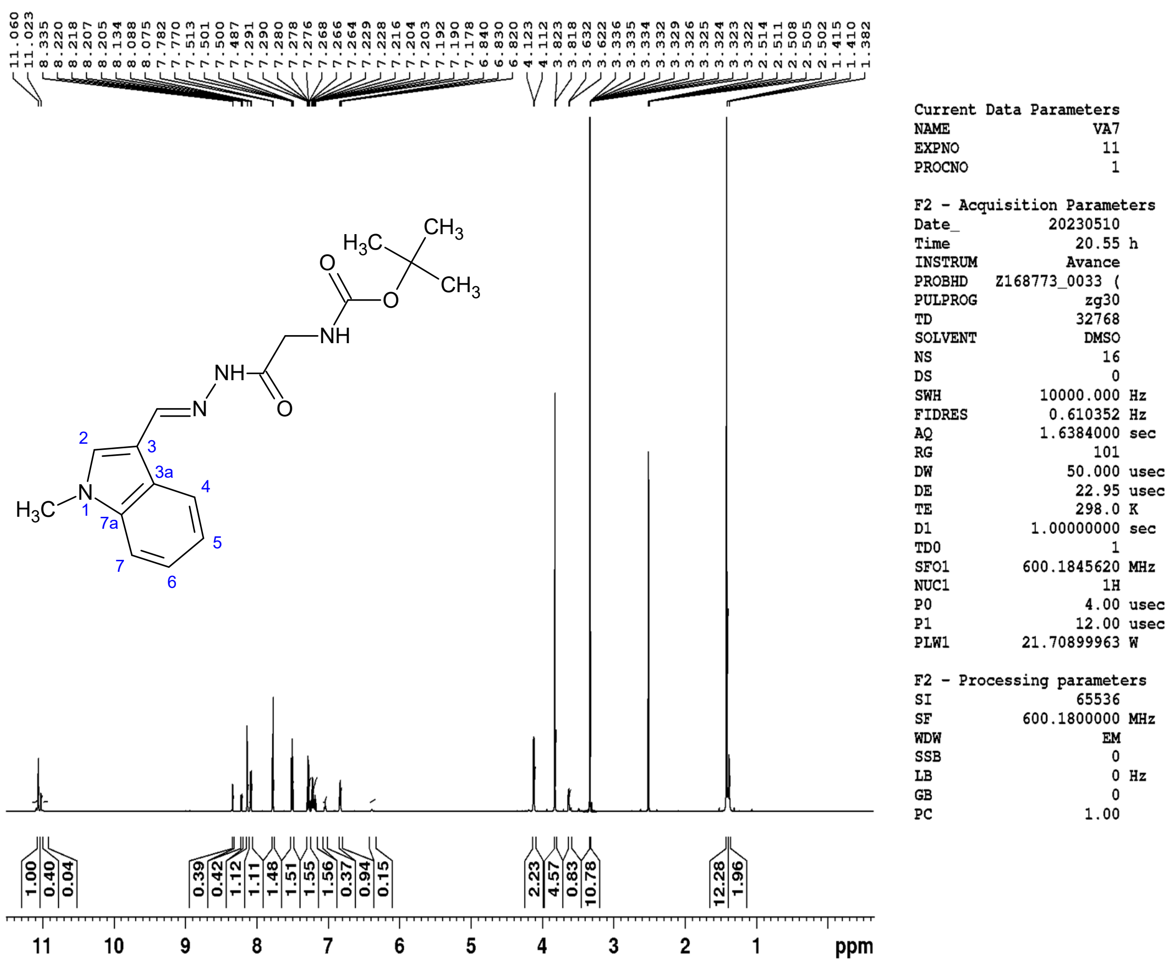

Figure S42. ^{1}H NMR spectrum of compound **3l** in DMSO-d_6

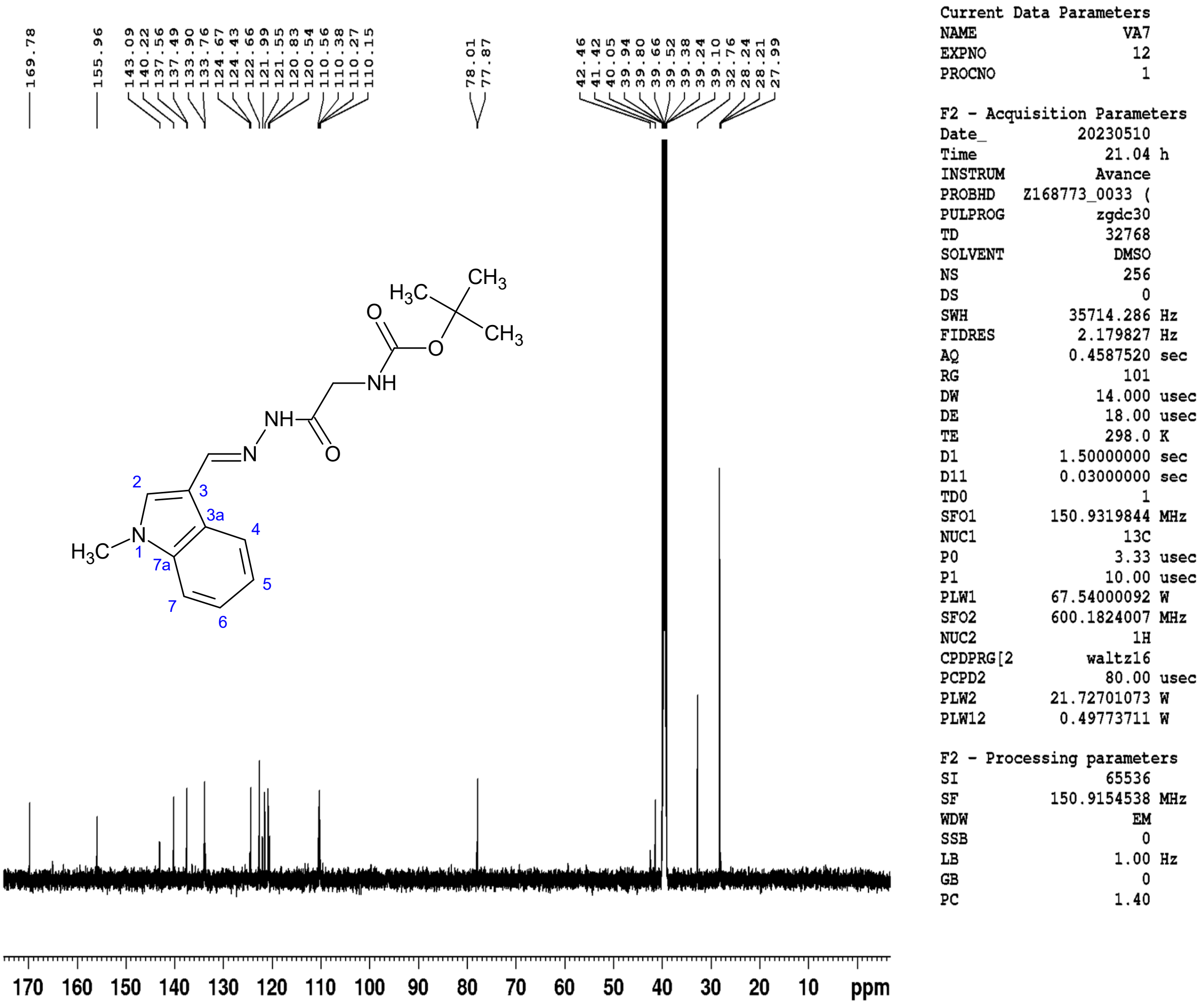

Figure S43. ^{13}C NMR spectrum of compound **3l** in DMSO-d_6

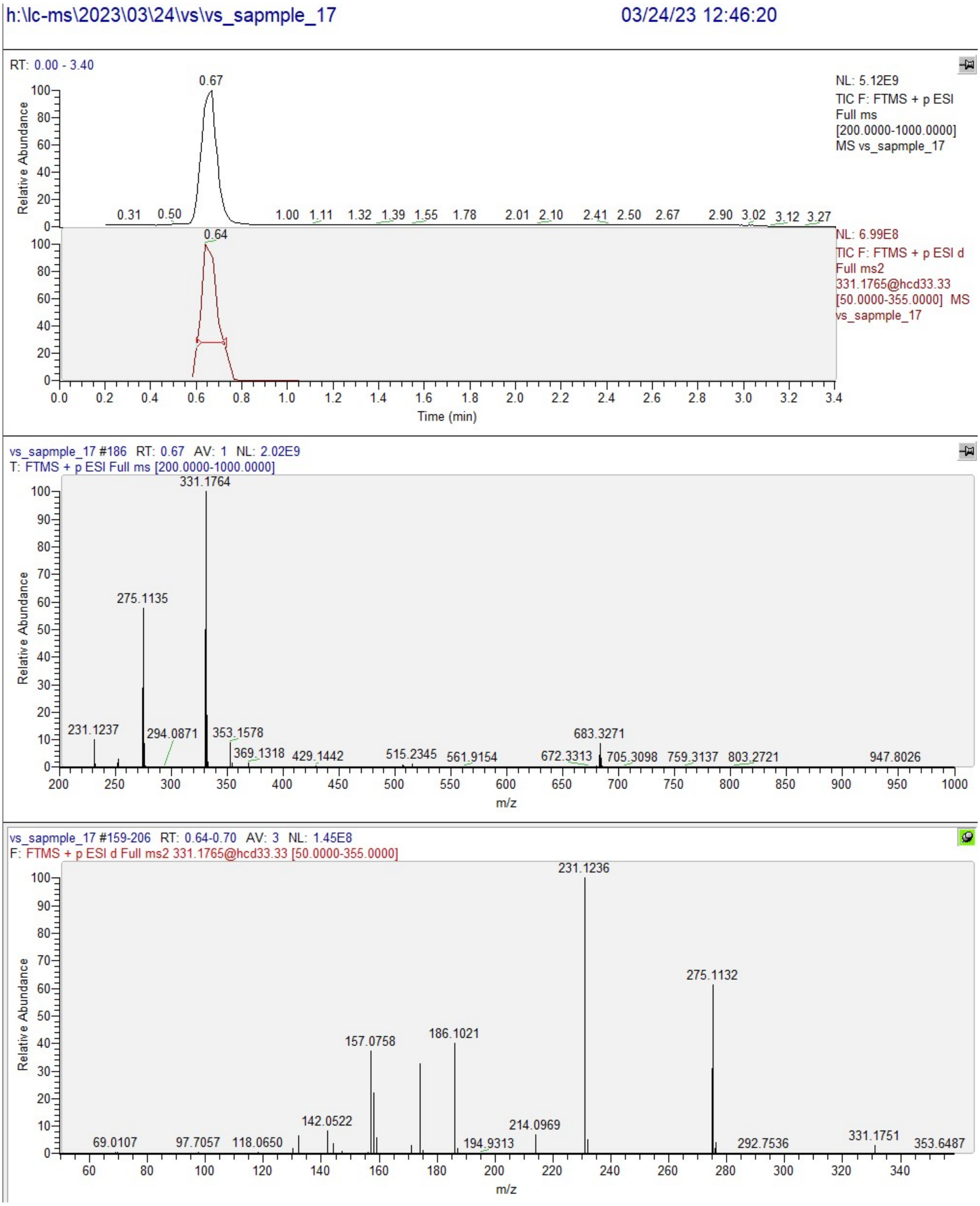

Figure S44. HRMS of compound **3l**

13. tert-butyl (2-{(2E)-2-[(1-benzyl-1H-indol-3-yl)methylidene]hydrazinyl}-2-oxoethyl)carbamate, **3m**

Yield: 90%; m.p. 207–208 ◦C.^{1}H NMR (600 MHz, DMSO-d$_6$): 1:0.40 mixture of conformers; signals for major *antiperiplanar* conformer around the amide bond: δ 11.10 (s, 1H, NH), 8.15 (s, 1H, CH), 8.08 (d, *J* = 8.3 Hz, 1H, H-4), 7.95 (s, 1H, H-2), 7.50 (d, *J* = 7.4 Hz, 1H, H-7), 7.30 (d, *J* = 7.5 Hz, 2H, o-Ph), 7.26-7.22 (m, 1H, H-6), 7.21-7.18 (m, 1H, H-5), 7.24 (t, *J* = 8.7 Hz, 2H, m-Ph), 7.20 (t, *J* = 8.0 Hz, 1H, p-Ph), 6.85 (t, *J* = 6.1 Hz, 1H, NH), 5.44 (s, 2H, CH$_2$), 4.10 (d, *J* = 6.1 Hz, 2H, CH$_2$), 1.40 (s, 9H, CH$_3$); resolved signals for minor *antiperiplanar* conformer around the amide bond: 7.05 (t, *J* = 6.0 Hz, 1H, NH), 8.21 (d, *J* = 7.9 Hz, 1H, H-4), 8.34 (s, 1H, CH) , 3.62 (d, *J* = 6.1 Hz, 2H, CH$_2$).

^{13}C NMR (151 MHz, DMSO-d$_6$): signals for major *antiperiplanar* conformer around the amide bond: δ 170.03 (C=O), 156.16 (O-C=O), 140.39 (CH), 137.68 (i-Ph), 137.02 (C-7a), 133.54 (C-2), 128.79 (C-o), 127.72 (p-Ph), 127.27 (m-Ph), 124.85 (C-3a), 123.03 (C-5), 121.87 (C-4), 121.18 (C-6), 111.15 (C-3), 110.93 (C-7), 78.12 (C), 49.45 (CH$_2$), 42.56 (CH$_2$), 28.38 (CH$_3$); resolved signals for minor *synperiplanar* conformer around the amide bond: 143.22 (CH), 122.29 (C-2), 78.27 (C), 41.57 (CH$_2$), 28.35 (CH$_3$). HRMS (ESI) m/z: calcd: [M+H]+ 407.207767. Found: [M+H]+ 407.2078

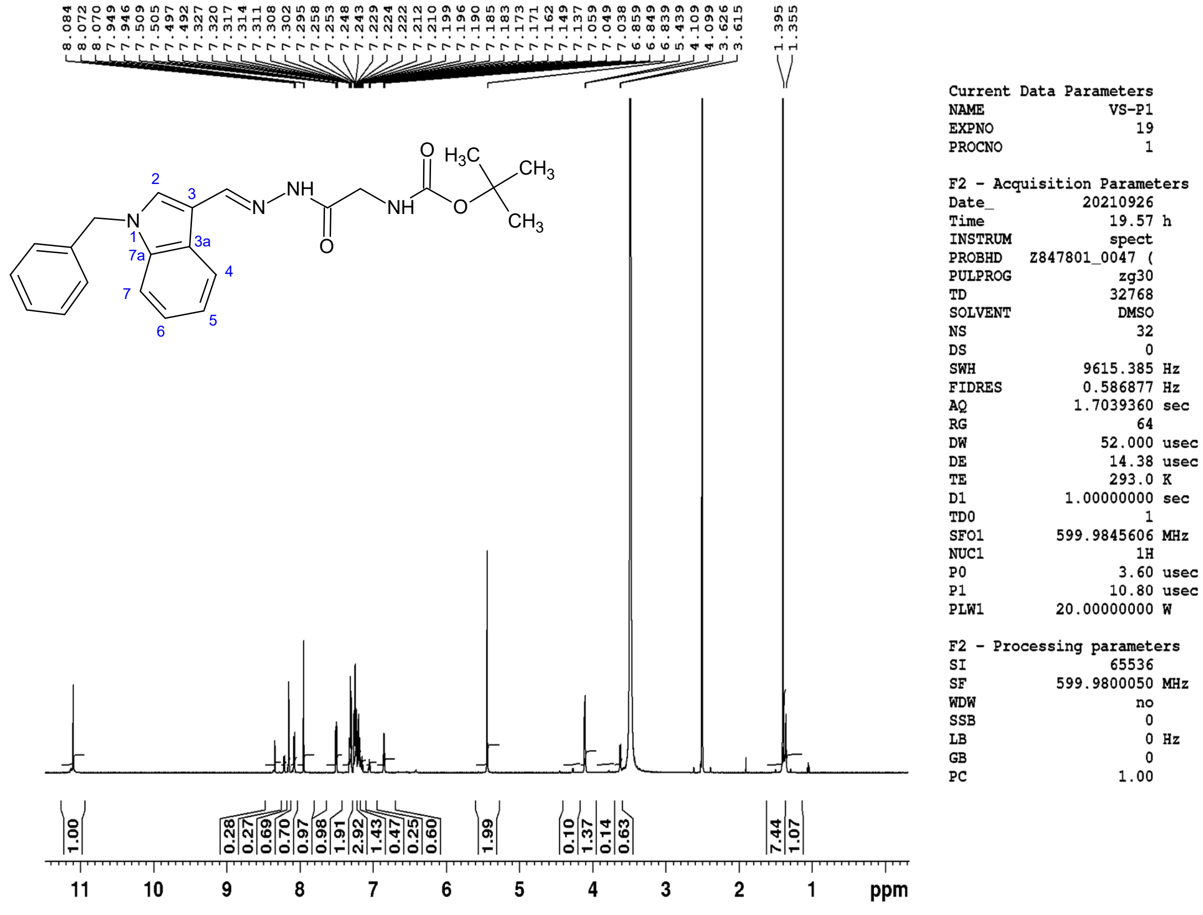

Figure S45. ^{1}H NMR spectrum of compound **3m** in DMSO-*d$_6$*

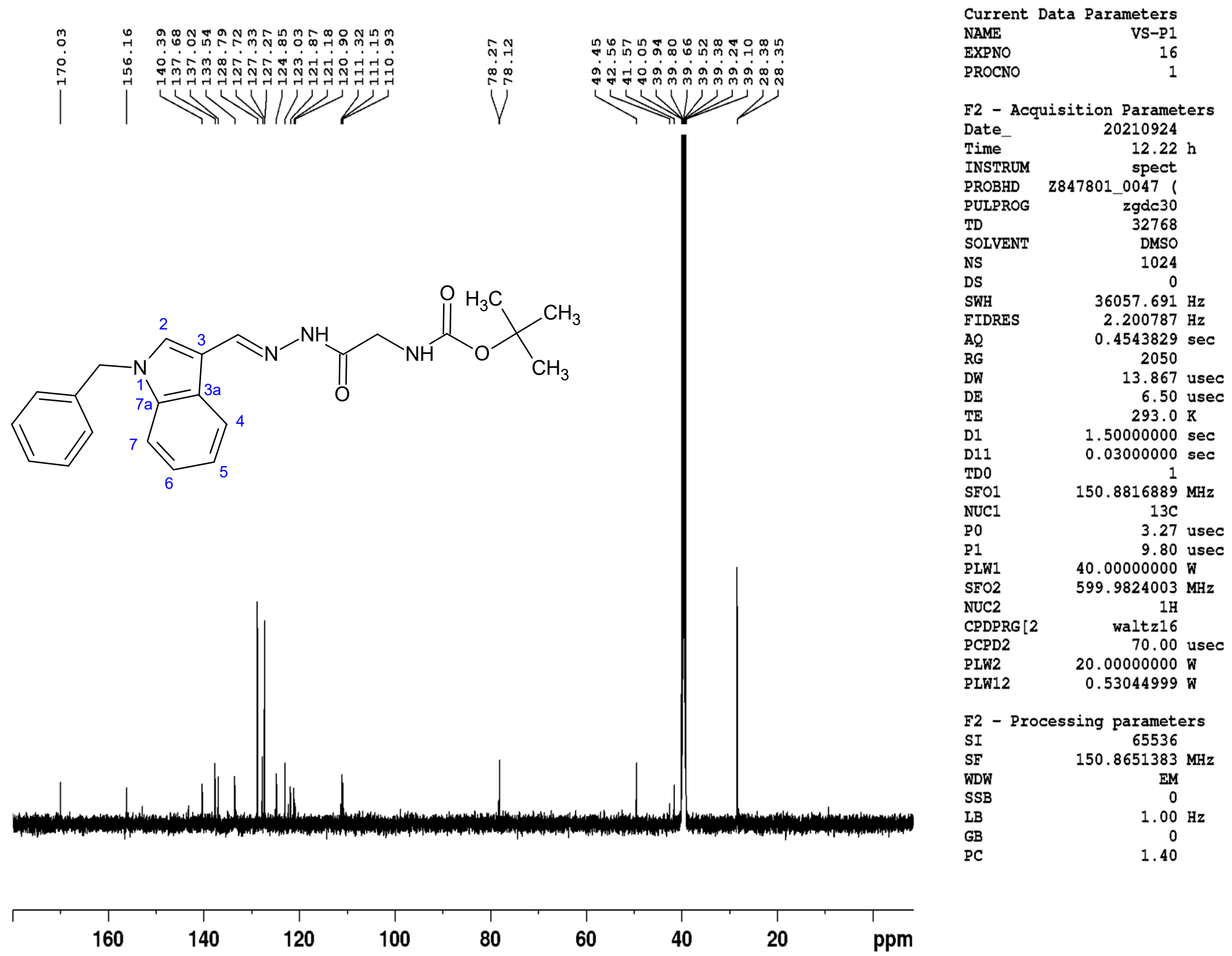

Figure S46. ^{13}C NMR spectrum of compound **3m** in DMSO-d_6

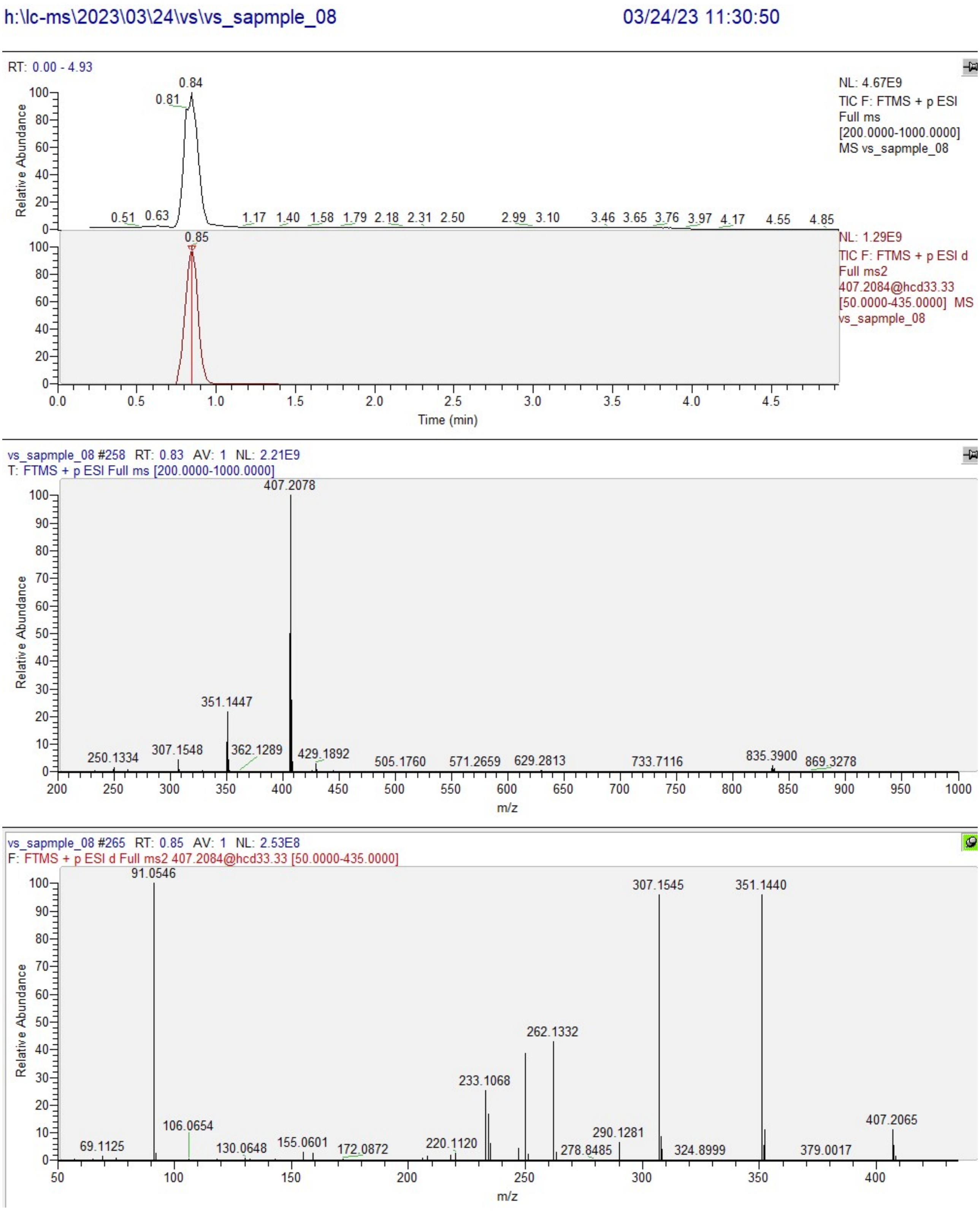

Figure S47. HRMS of compound **3m**

14. 2-(1*H*-indol-3-yl)-*N*'-[(*E*)-(5-methoxy-1*H*-indol-3-yl)methylidene]acetohydrazide, 3n [1]

^{1}H NMR, ^{13}C NMR and HRMS spectra of compound **3n** can be found in https://doi.org/10.1016/j.bmcl.2021.128516

***15. N'-[(E)-(3,4-dimethoxyphenyl)methylidene]-1H-indole-3-carbohydrazide,* 3o**

Yield: 78%; m.p. 267-270◦C. ^{1}H NMR (400 MHz, DMSO-d$_6$, 353K): δ = 3.83 (s, 3H, OCH_3), 3.84 (s, 3H, OCH_3), 7.03 (d, J=8.3 Hz, 1H, H-5'), 7.14 (ddd, J=1.3, 7.0, 7.7 Hz, 1H, H-5), 7.18 (dt, J=1.6, 6.6 Hz, 1H, H-6), 7.20 (dd, J=1.6, 7.2 Hz, 1H, H-6'), 7.35 (d, J=1.9 Hz, 1H, H-2'), 7.48 (d, J=8.0 Hz, 1H, H-7), 8.21 (d, J=7.6 Hz, 1H, H-4), 8.26 (s, 1H, CH), 10.98 (bs, 1H, NH), 11.52 (bs, 1H, NH). ^{13}C NMR (151 MHz, DMSO-d$_6$, 353K): δ = 55.48 (CH_3), 55.56 (CH_3), 108.45 (C-3), 109.14 (C-2'), 111.45 (C-5'), 112.05 (C-7), 120.22 (C-4), 120.74 (C-5), 120.89 (C-6'), 121.73 (C-6), 126.53 (C-1'), 127.66 (C-3a), 129.17 (C-2), 135.69 (C-7a), 144.03 (CH), 149.15 (C-3'), 150.35 (C-4'), 162.13 (C=O). HRMS (ESI) *m/z*: calcd: [M+H]+ 324.134268. Found: [M+H]+ 324.13358

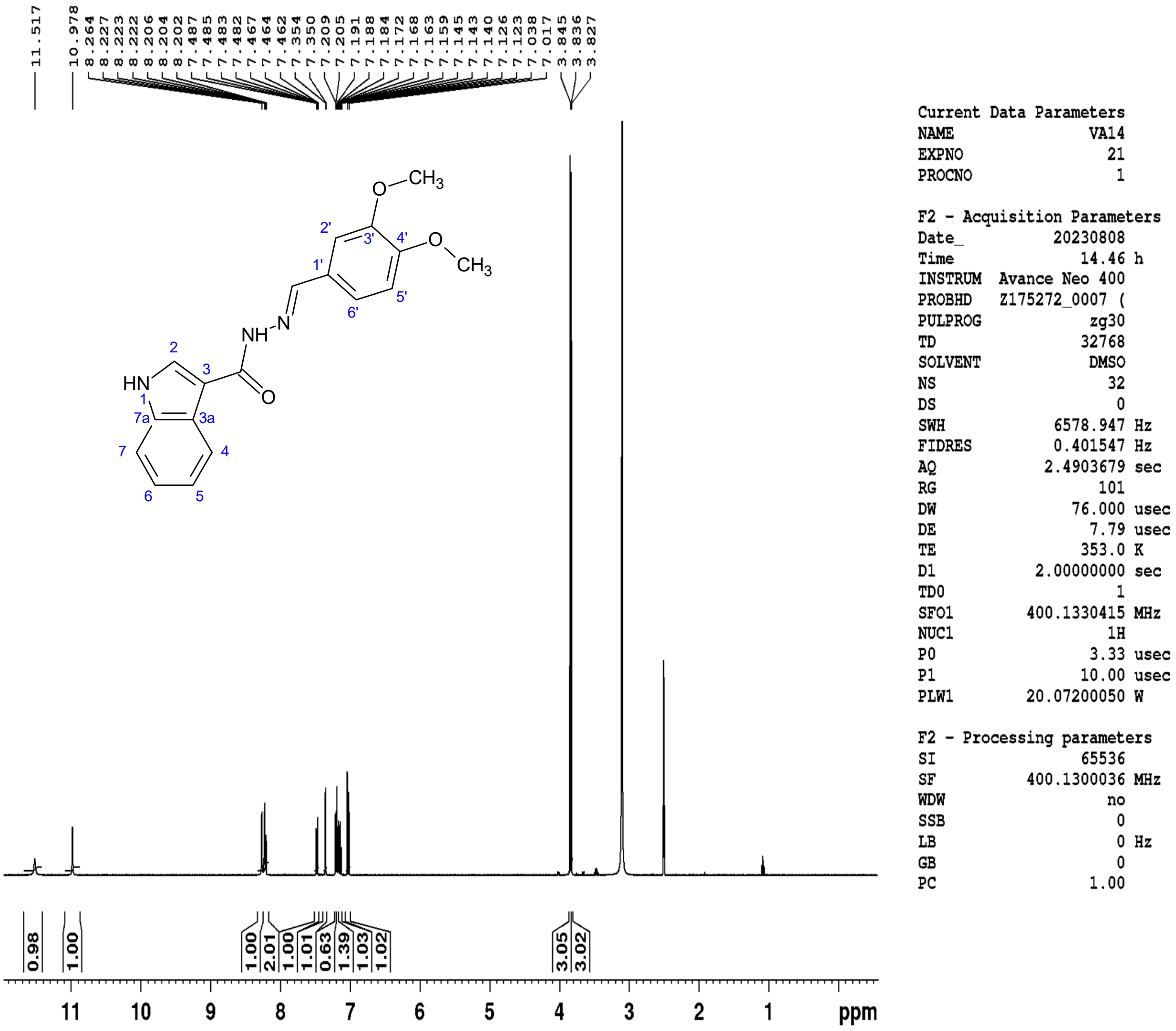

Figure S48. ^{1}H NMR spectrum of compound **3o** in DMSO-d_6

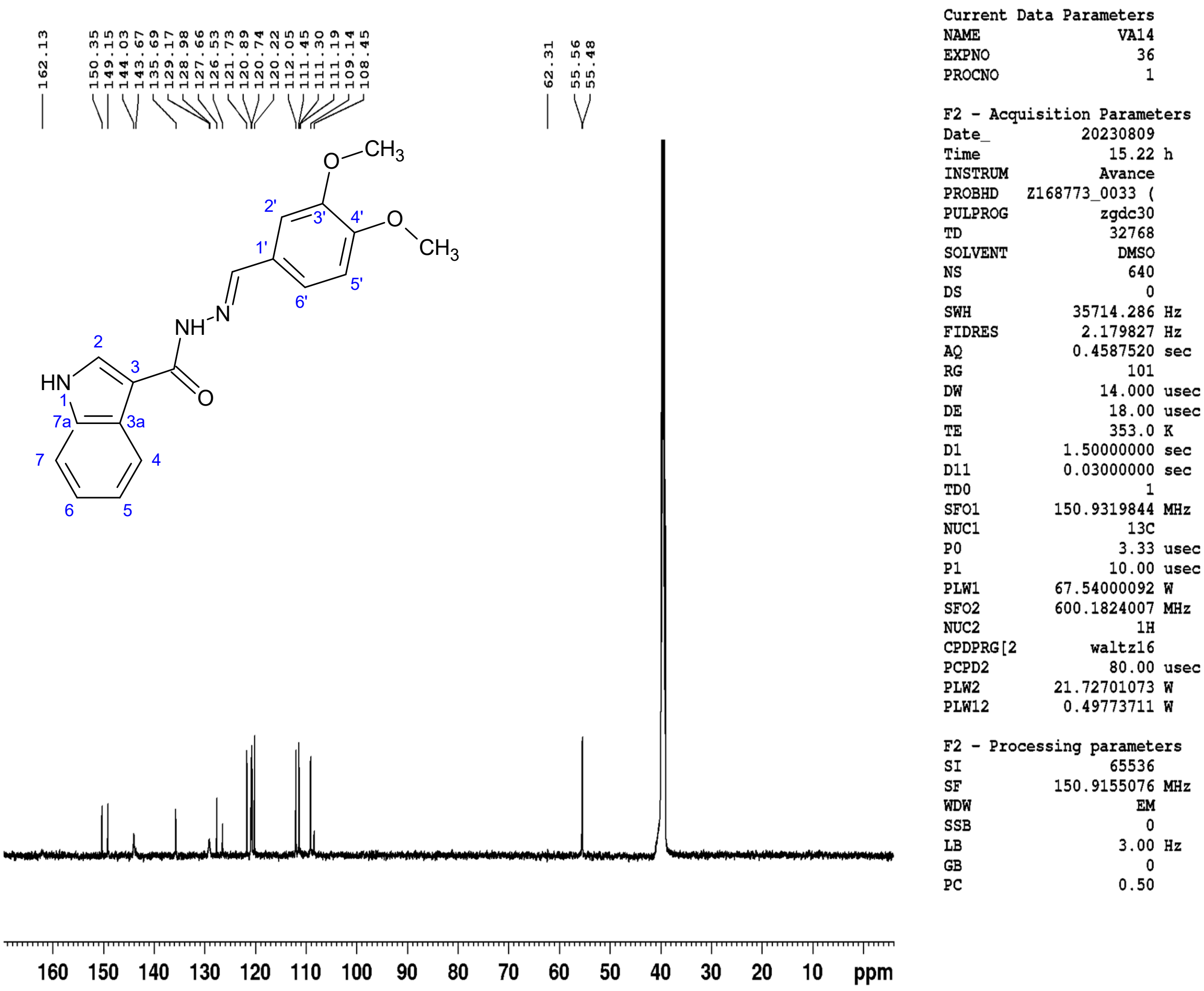

Figure S49. ^{13}C NMR spectrum of compound **3o** in DMSO-d_6

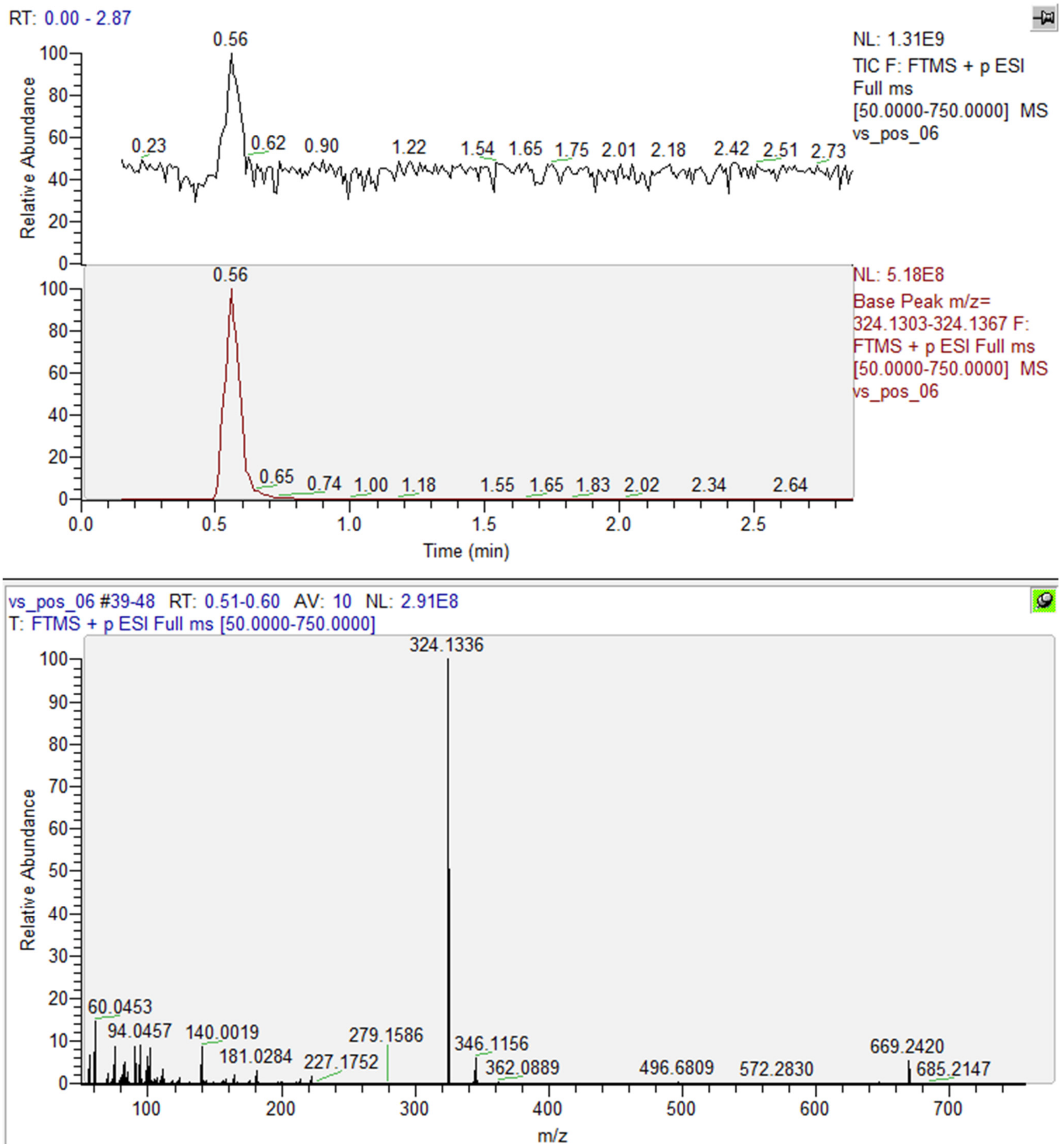

Figure S50. HRMS of compound **3o**

16. N'-[(E)-(3,4-dimethoxyphenyl)methylidene]-2-(1H-indol-3-yl)acetohydrazide, 3p

Yield: 92%; m.p. 177-180◦C. ^{1}H NMR (600 MHz, DMSO-d$_6$): 1:0.80 mixture of conformers; signals for major *antiperiplanar* conformer around the amide bond: δ = 3.80 (s, 3H, OCH_3), 3.81 (s, 3H, OCH_3), 4.05 (s, 2H, CH_2), 6.95 (t, J=7.5 Hz, 1H, H-5), 7.01 (d, 8.5 Hz, 2H, H-5'), 7.06 (t, J=7.0 Hz, 1H, H-6), 7.17 (dt, J=1.8, 8.7 Hz, 2H, H-6'), 7.28 (d, J=1.7 Hz, 1H, H-2'), 7.35 (d, J=2.42 Hz, 1H, H-2), 7.36 (d, J=9.27 Hz, 1H, H-7), 7.60 (dd, J=3.46, 7.85 Hz, 1H, H-4), 7.91 (s, 1H, CH), 10.87 (bs, 1H, NH), 11.18 (s, 1H, NH),; resolved signals for minor *antiperiplanar* conformer around the amide bond: 8.15 (s, 1H, CH), 10.91 (bs, 1H, NH), 11.40 (s, 1H, NH)

^{13}C NMR (151 MHz, DMSO-d$_6$): signals for major *antiperiplanar* conformer around the amide bond: δ = 29.24 (CH_2), 55.40 (OCH_3), 108.22 (C-2'), 108.25 (C-3), 111.30 (C-5'), 111.56 (C-7), 118.28 (C-4), 118.66 (C-5), 120.97 (C-6'), 121.59 (C-6), 123.86 (C-2), 127.13 (C-1'), 127.39 (C-3a), 135.98 (C-7a), 142.46 (CH), 148.99 (C-3'), 150.56 (C-4'), 172.52 (C=O). HRMS (ESI) m/z: calcd: [M+H]+ 338.149918. Found: [M+H]+ 338.14902

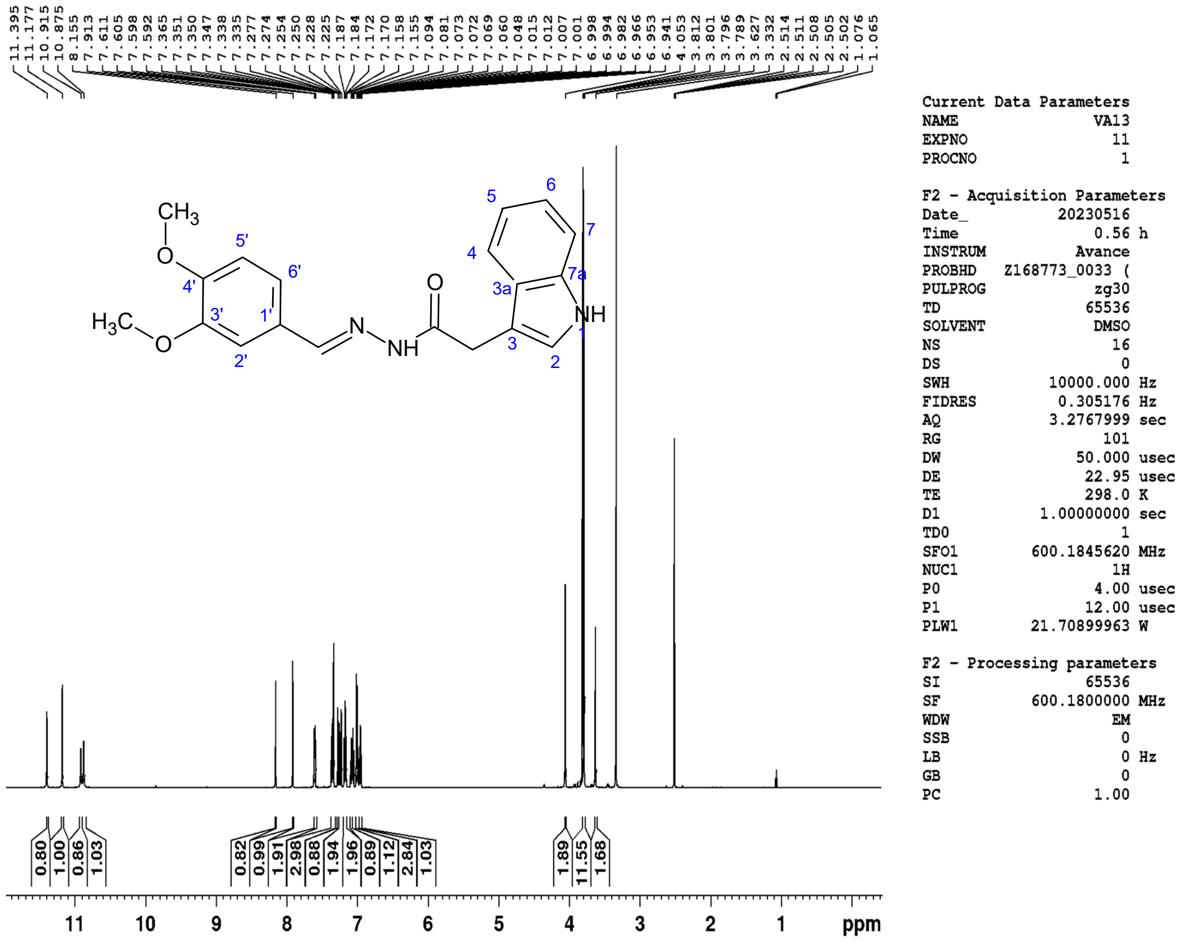

Figure S51. ^{1}H NMR spectrum of compound **3p** in DMSO-d_6

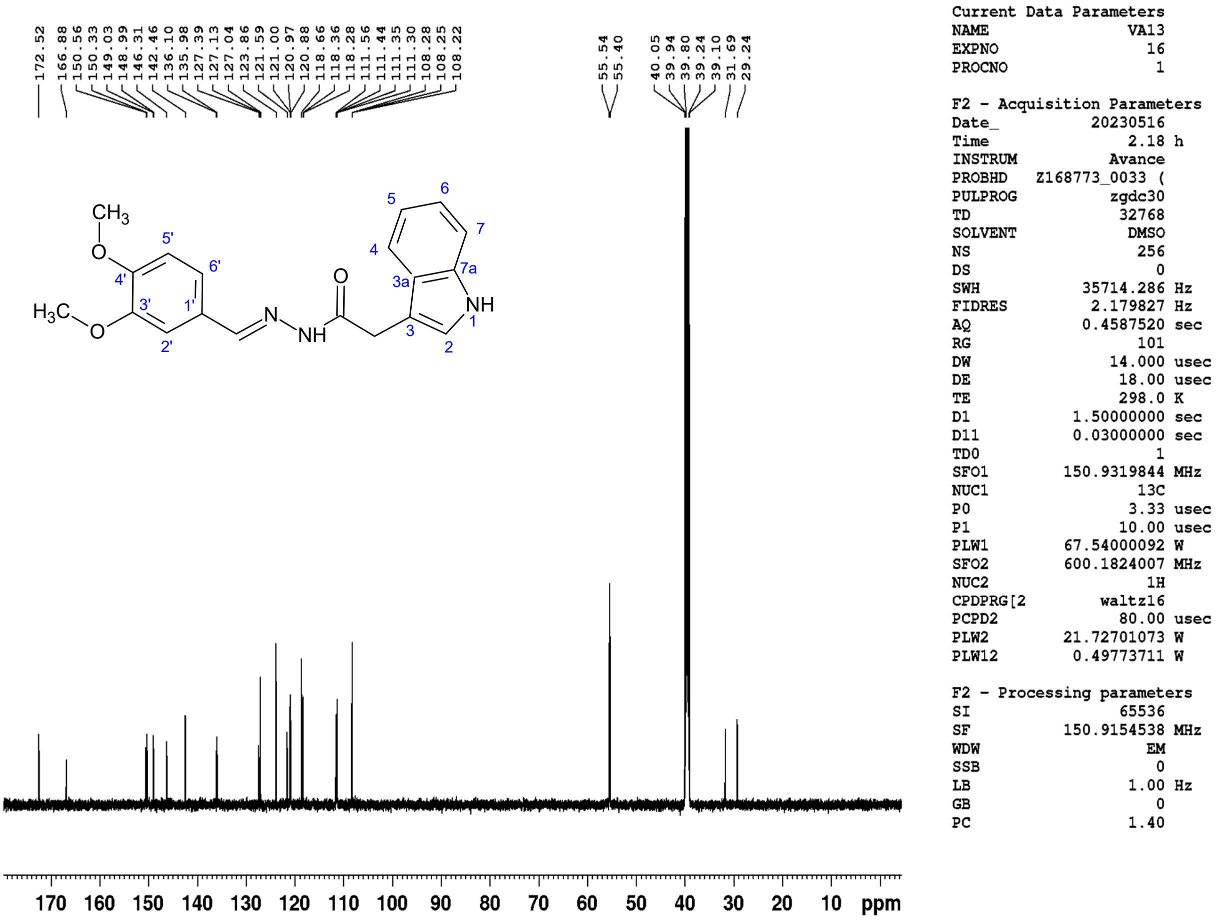

Figure S52. ^{13}C NMR spectrum of compound **3p** in DMSO-d_6

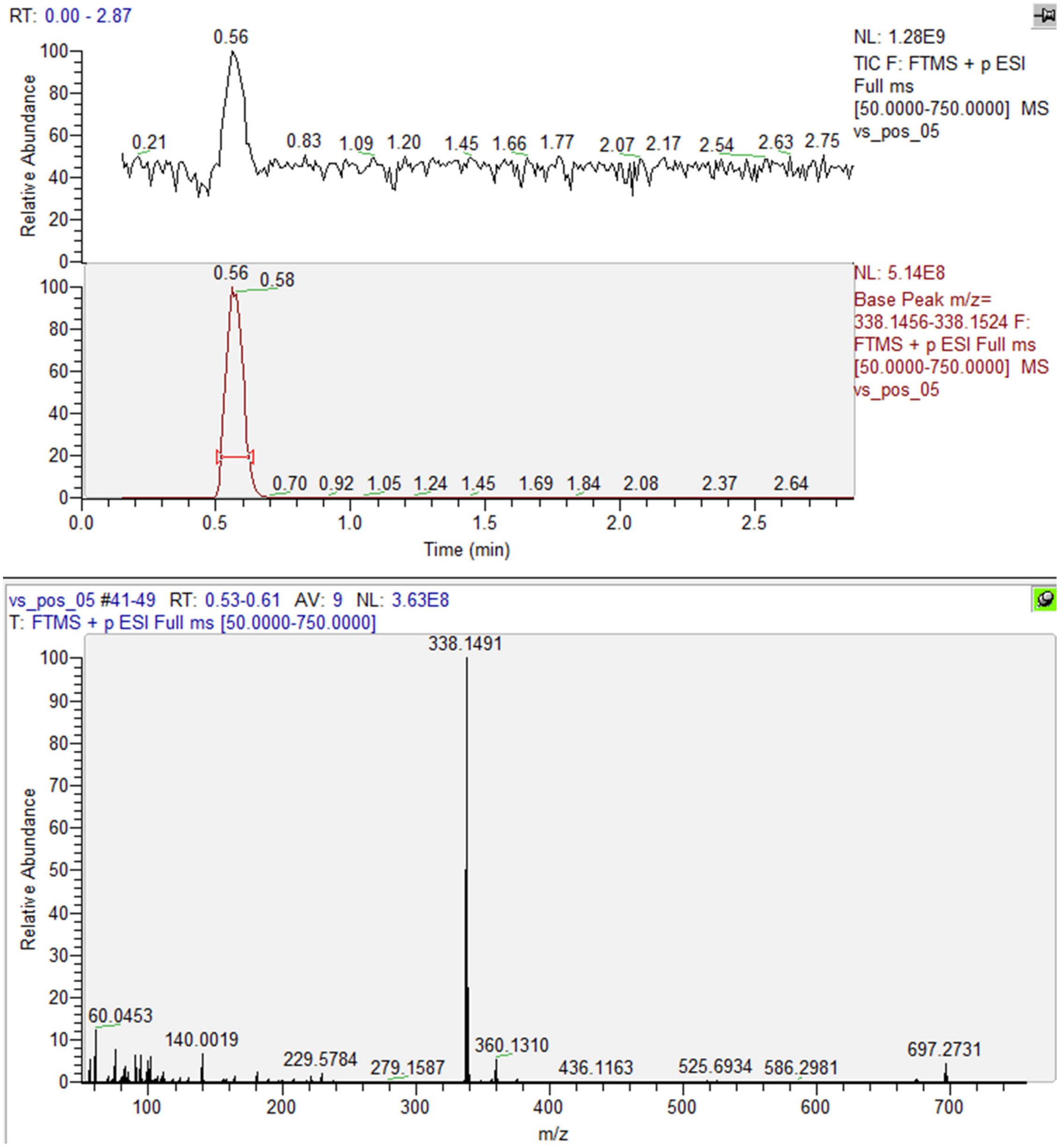

Figure S53. HRMS of compound **3p**

17. N'-[(E)-(4-hydroxy-3-methoxyphenyl)methylidene]-2-(1H-indol-3-yl)acetohydrazide, 3q

Yield: 72%; m.p. 246-247◦C. ^{1}H-NMR (400 MHz, DMSO-d$_6$): 0.54 : 0.46 mixture of two conformers; signals for major conformer: δ = 3.81 (s, 3H, CH3), 4.04 (s, 2H, CH2), 6.82 (d, J=8.1 Hz, 1H, H-5'), 6.96-7.00 (m, 1H, H-5), 7.02-7.09 (m, 2H, H-6 and H-6'), 7.22 (d, J=2.3 Hz, 1H, H-2), 7.29 (d, J=1.8 Hz, 1H, H-2'), 7.33 (d, J=6.8 Hz, 1H, H-7), 7.59 (d, J=7.9 Hz, 1H, H-4), 7.87 (s, 1H, CH), 9.46 (s, 1H, OH), 10.86 (s, 1H, NH), 11.10 (s, 1H, NH); Signals for minor conformer: δ(ppm): 3.61 (s, 2H, CH2), 3.79 (s, 3H, CH3), 6.81 (d, J=8.1 Hz, 1H, H-5'), 6.93-6.97 (m, 1H, H-5), 7.02-7.09 (m, 2H, H-6 and H-6'),7.24 (d, J=1.9 Hz, 2H, H-2 and H-2'), 7.35 (d, J=7.0 Hz, 1H, H-7), 7.59 (d, J=7.9 Hz, 1H, H-4), 8.11 (s, 1H, CH), 9.48 (s, 1H, OH), 10.90 (s, 1H, NH), 11.33 (s, 1H, NH).

^{13}C-NMR (151 MHz, DMSO-d_6): 0.54 : 0.46 mixture of two conformers; signals for major conformer: δ = 29.20 (CH2), 55.51 (CH3), 108.33 (C-3), 109.10 (C-2’), 111.30 (C-7), 115.37 (C-5’), 118.27 (C-5), 118.68 (C-4), 120.88 (C-6’), 121.11 (C-6), 123.86 (C-2), 125.82 (C-1’), 127.42 (C-4a), 135.98 (C-7a), 142.81 (CH), 147.97 (C-3’), 148.51 (C-4’), 172.43 (C=O); Signals for minor conformer: δ(ppm): 31.69 (CH2), 55.51 (CH3), 108.31 (C-3), 108.93 (C-2’), 111.35 (C-7), 115.52 (C5’), 118.36 (C-5), 118.68 (C-4), 121.00 (C-6’), 121.87 (C-6), 123.86 (C-2), 125.73 (C-1’), 127.14 (C-4a), 136.10 (C-7a), 146.65 (CH), 147.97 (C-3’), 148.78 (C-4’), 166.79 (C=O).HRMS (ESI) m/z: calcd: [M+H]+ 324.134268. Found: [M+H]+ 324.1341

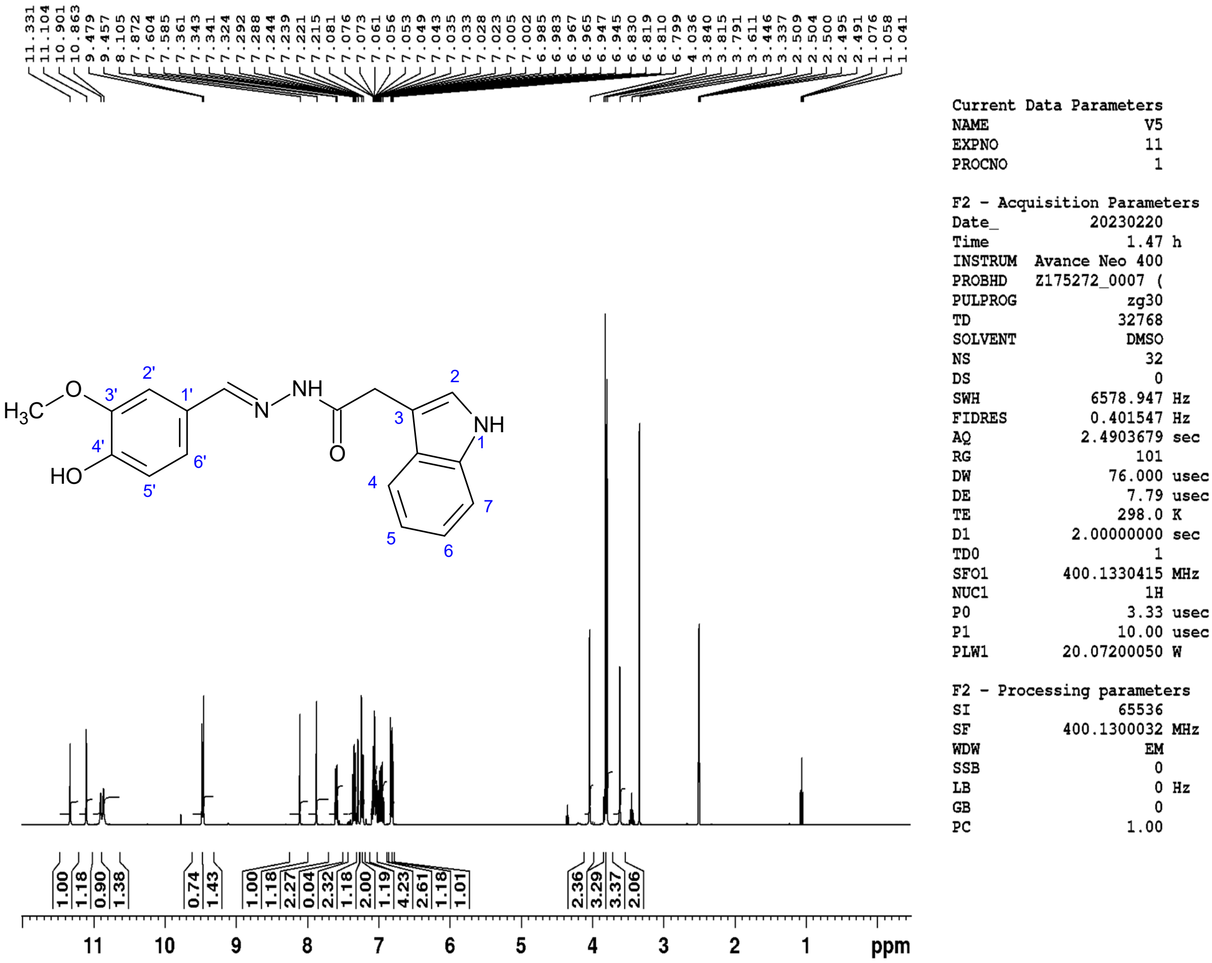

Figure S54. ^{1}H NMR spectrum of compound **3q** in DMSO-d_6

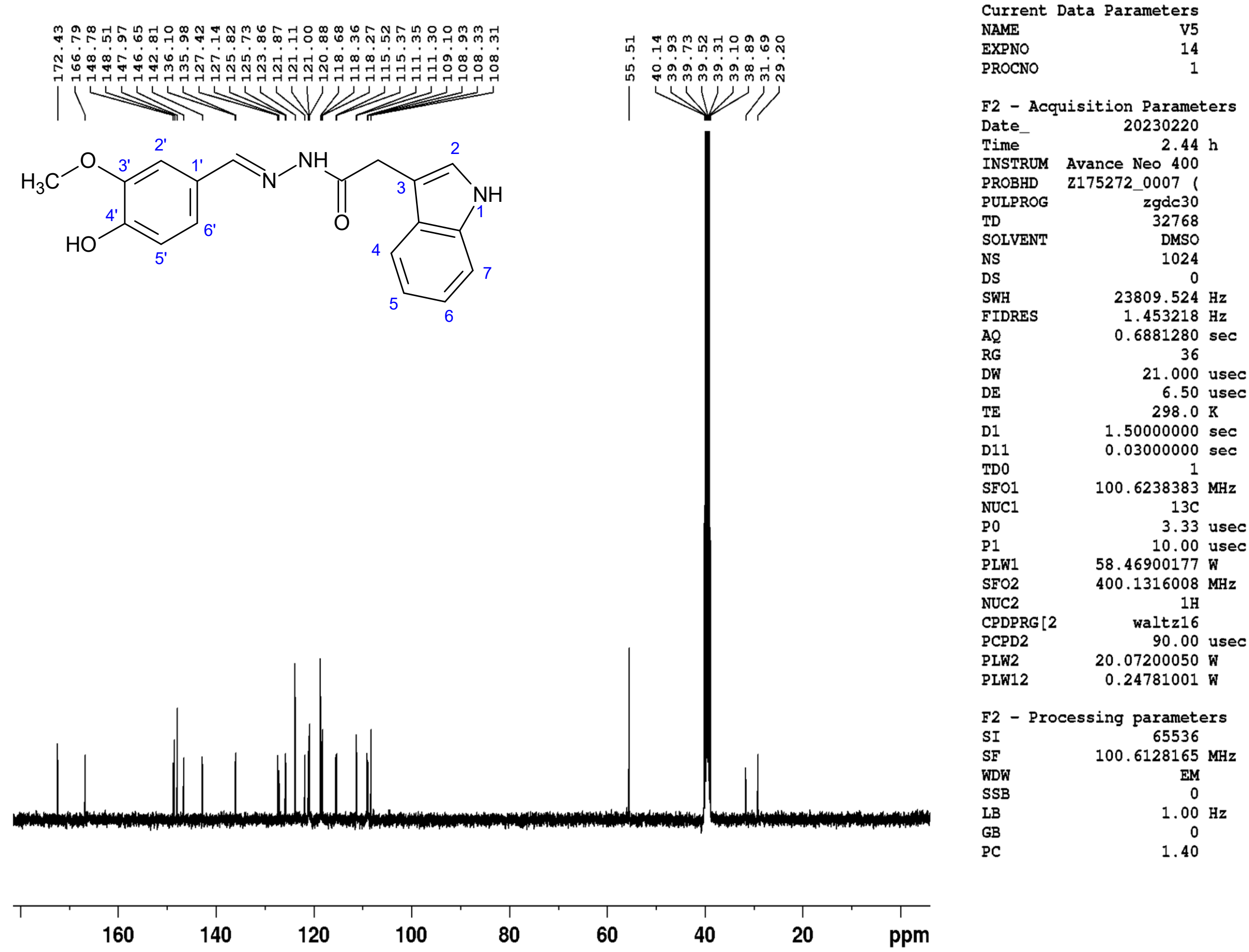

Figure S55. ^{13}C NMR spectrum of compound **3q** in DMSO-d_6

h:\lc-ms\2023\03\24\vs\vs_sapmple_04 03/24/23 10:48:35

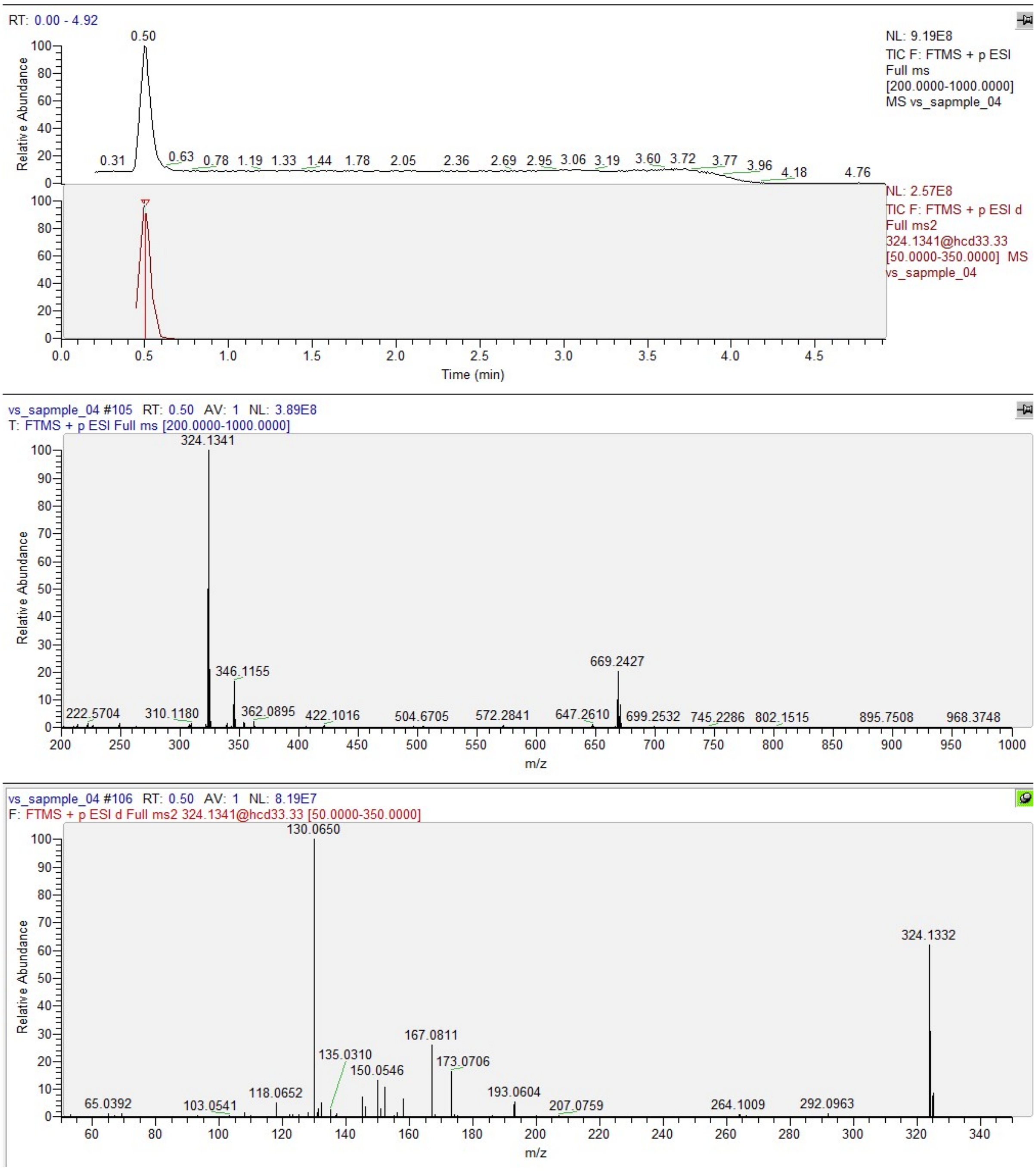

Figure S56. HRMS of compound **3q**

***18. 2,4-dihydroxy-N'-[(E)-(4-hydroxy-3-methoxyphenyl)methylidene]benzohydrazide,* 3r**

Yield: 76%; m.p. 242-243◦C. ^{1}H NMR (400 MHz, DMSO-d_6): 3.36 (s, 3H, CH_3), 6.31 (d, J=2.4 Hz, 1H, H-3), 6.37 (dd, J=2.4, 8.7 Hz, 1H, H-5), 6.85 (d, J=8.1 Hz, 1H, H-6'), 7.10 (dd, J=1.8, 8.1 Hz, 1H, H-5'), 7.32 (d, J=1.8 Hz, 1H, H-2'), 7.80 (d, J=8.7 Hz, 1H, H-6), 8.33 (s, 1H, CH), 9.56 (s, 1H, OH), 10.19 (s, 1H, OH), 11.54 (s, 1H, NH), 12.45 (s, 1H, OH).

^{13}C NMR (100 MHz, DMSO-d_6): 55.55 (CH_3), 102.86 (C-3), 106.20 (C-1), 107.33 (C-5), 108.99 (C-2'), 115.45 (C-6'), 122.27 (C-5'), 125.59 (C-1'), 129.49 (C-6), 148.05 (C-4'), 148.61 (CH), 149.09 (C-3'), 162.31 (C-4), 162.57 (C-2), 165.28 (C=O).

HRMS (ESI) m/z: calcd: [M+H]+ 303.097548. Found: [M+H]+ 303.0974

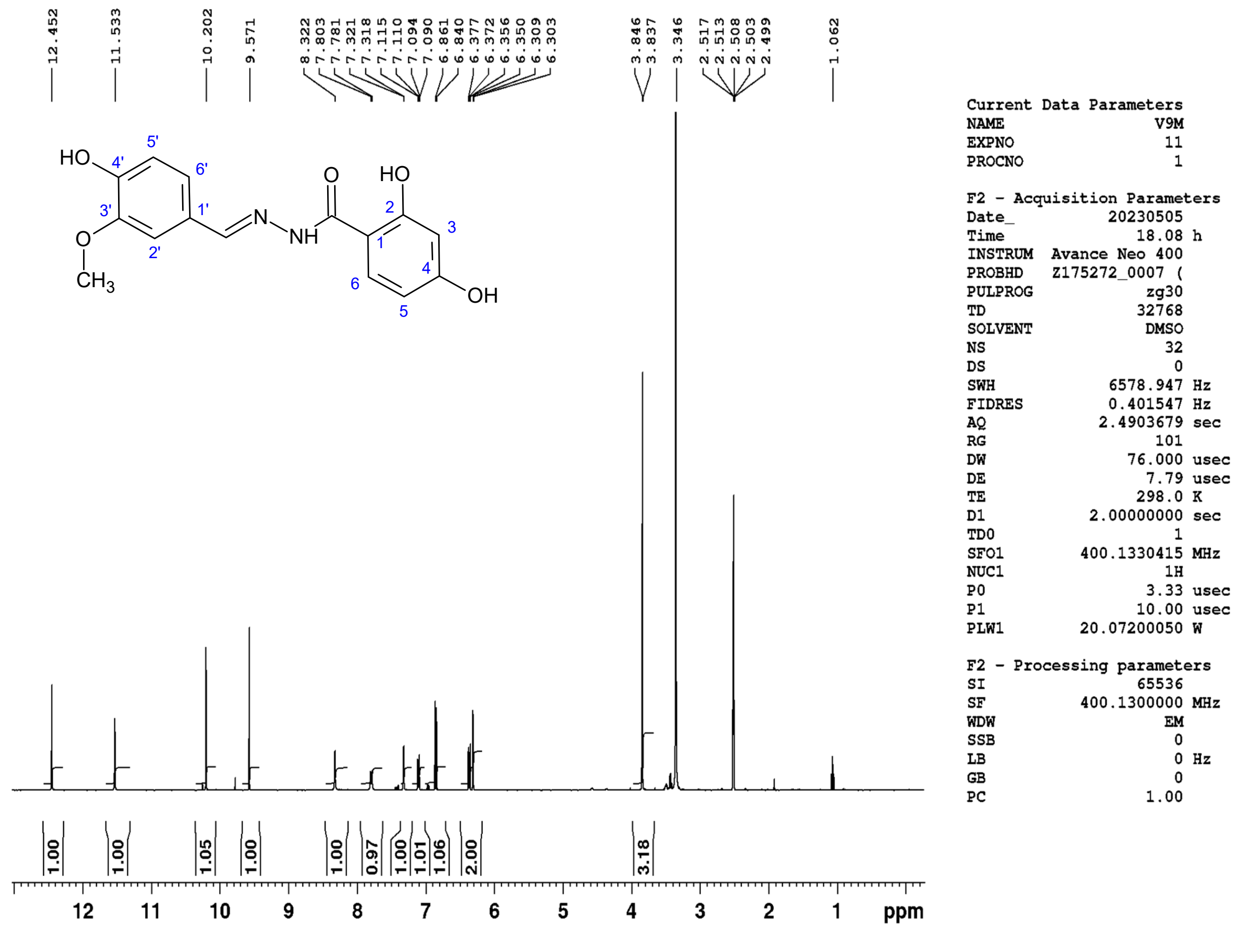

Figure S57. ^{1}H NMR spectrum of compound **3r** in DMSO-d_6

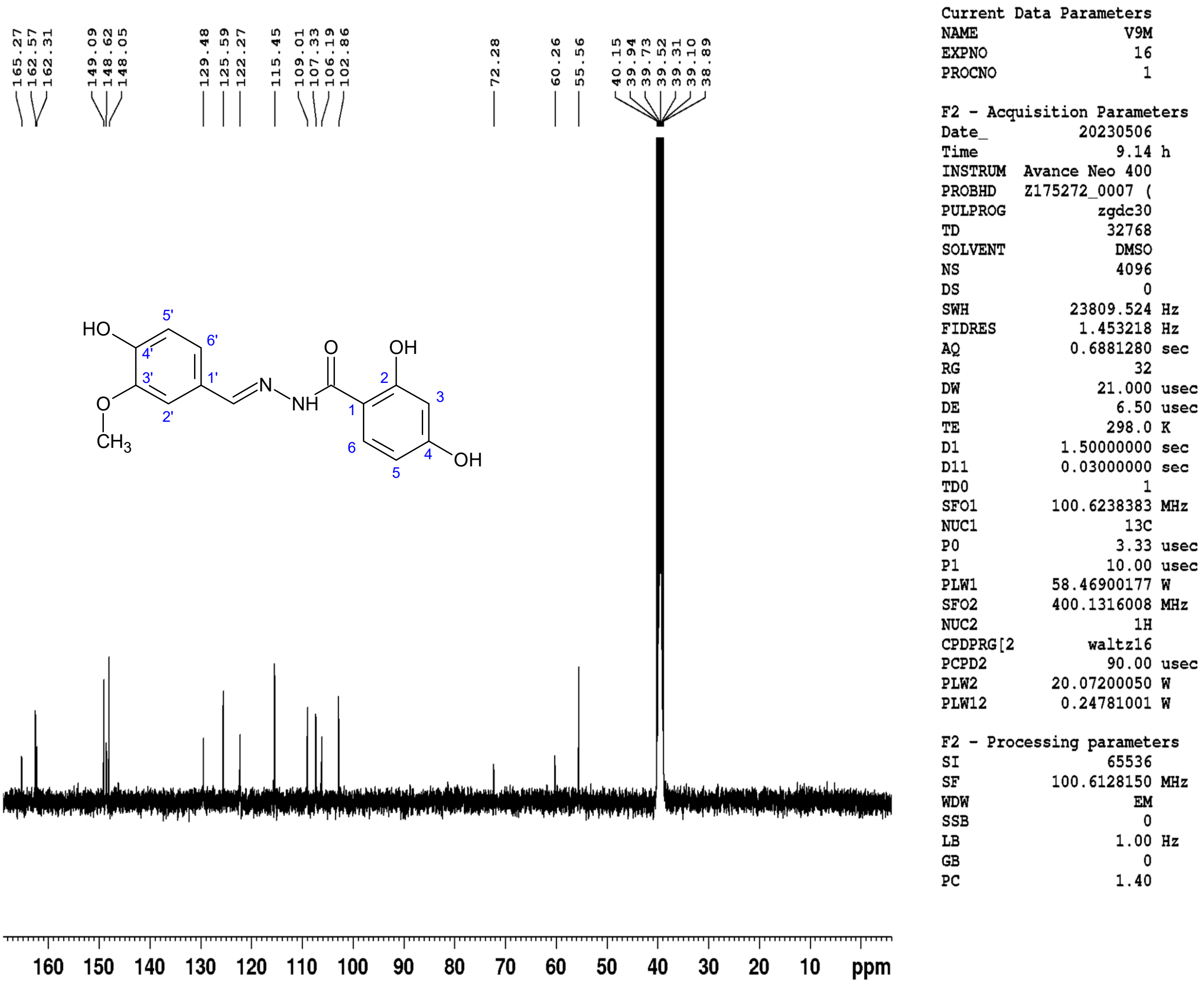

Figure S58. ^{13}C NMR spectrum of compound **3r** in DMSO-d_6

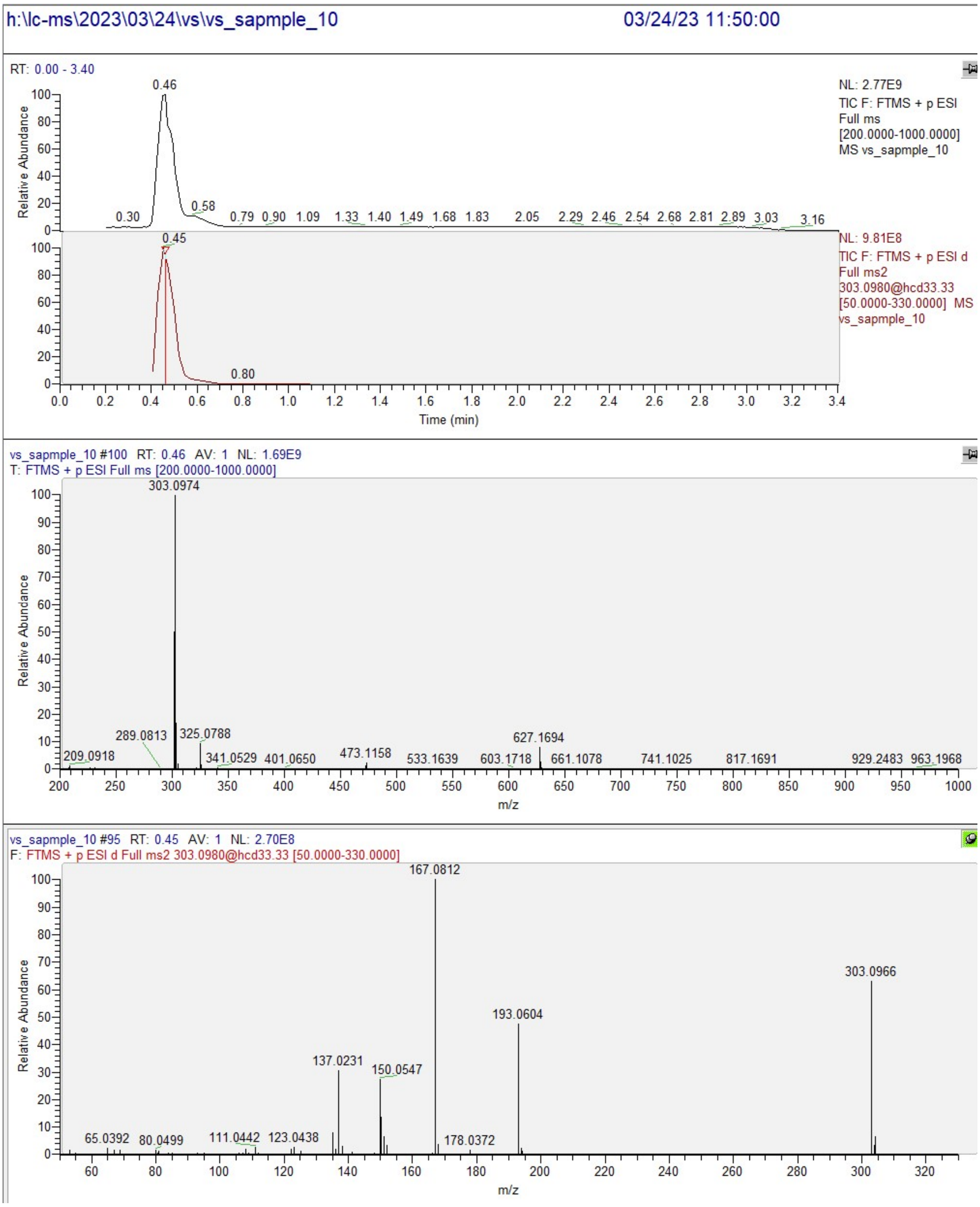

Figure S59. HRMS of compound **3r**

***19. N'-[(E)-(1-benzylpiperidin-4-yl)methylidene]benzenesulfonohydrazide,* 5a**

Yield: 59%; m.p. 110-111◦C.^{1}H NMR (400 MHz, DMSO-d$_6$): δ = 1.26-1.36 (m, 2H, CH_2), 1.54-1.58 (m, 2H, CH_2), 1.91-1.97 (m, 2H, CH_2), 2.05-2.14 (m, 1H CH), 2.65-2.68 (m, 2H, CH_2), 3.40 (s, 2H, CH_2), 7.19 (d, J=4.8 Hz, 1H, CH), 7.21-7.27 (m, 3H, H-2'', H-4'' and H-6''), 7.29-7.33 (m, 2H, H-3'' and H-5''), 7.59-7.63 (m, 2H, H-3' and H-5'), 7.65-7.69 (m, 1H, H-4'), 7.78-7.81 (m, 2H, H-2' and H-6'), 10.93 (1H, s).

^{13}C NMR (DMSO) (100 MHz, DMSO-d$_6$): δ = 27.62 (CH_2), 36.94 (CH), 51.11 (CH_2), 61.25 (CH_2), 125.85 (C-4''), 126.12 (C-2' and C-6'), 127.11 (C-3'' and C-5''), 127.75 (C-2'' and C-6''), 128.07 (C-3' and C-5'), 131.86 (C-4'), 137.28 (C-1''), 137.94 (C-1'), 153.47 (CH).HRMS (ESI) m/z: calcd: [M+H]+ 358.158373. Found: [M+H]+ 358.1581

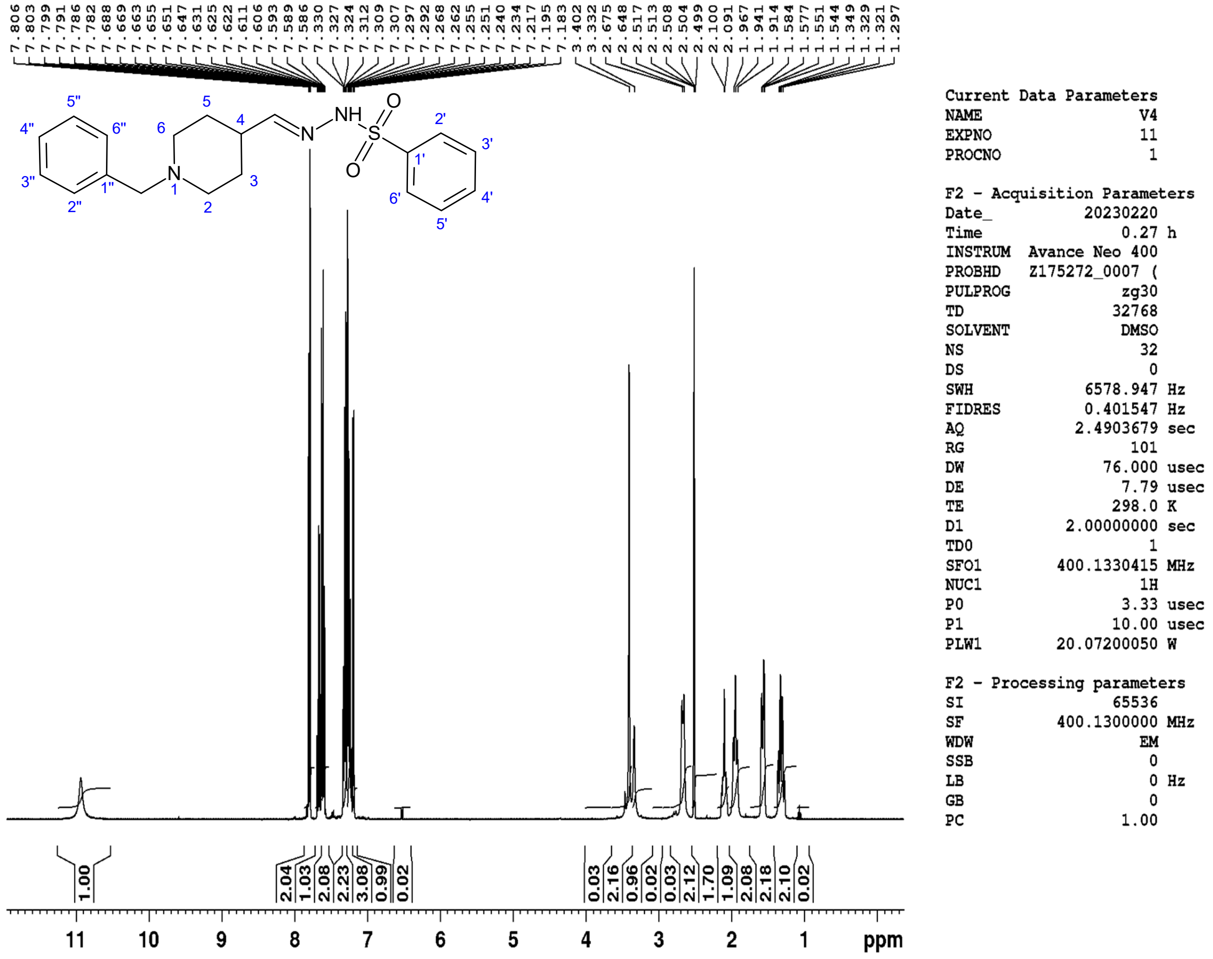

Figure S60. ^{1}H NMR spectrum of compound **5a** in DMSO-d_6

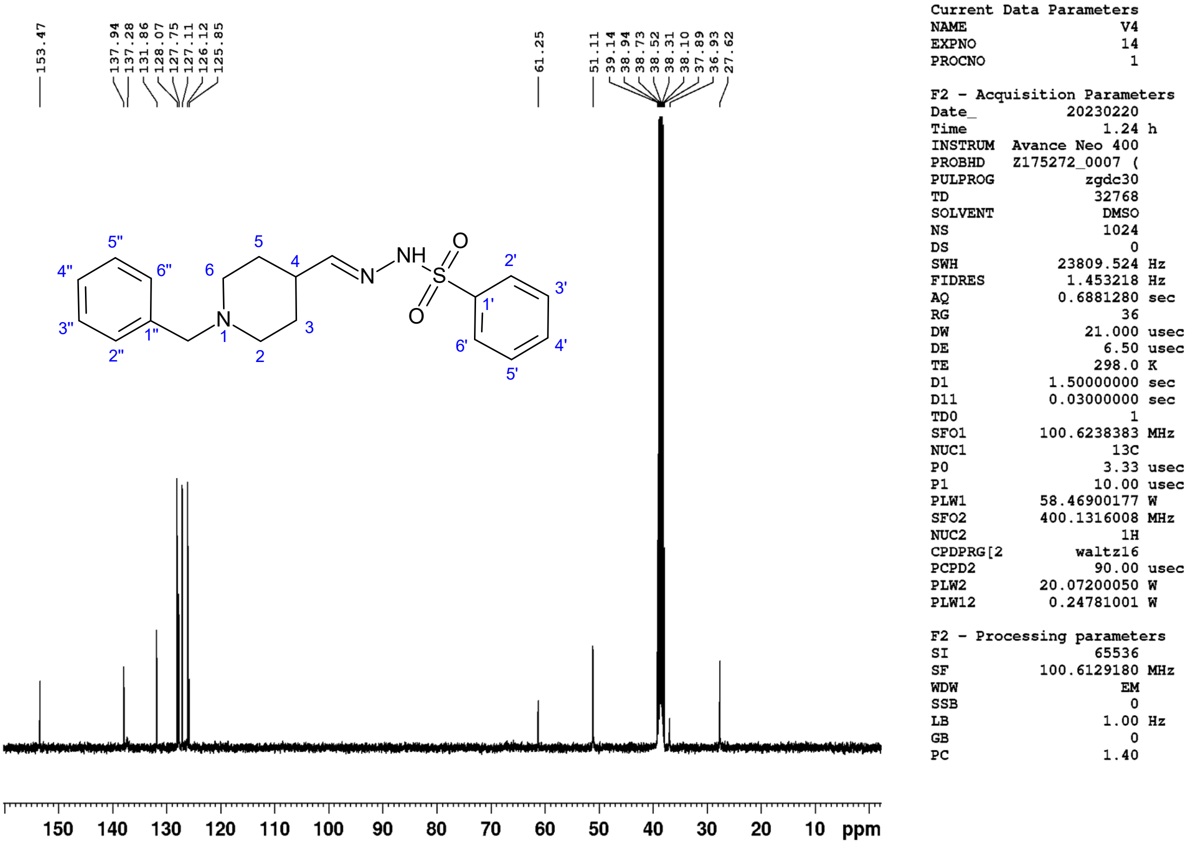

Figure S61. ^{13}C NMR spectrum of compound **5a** in DMSO-d_6

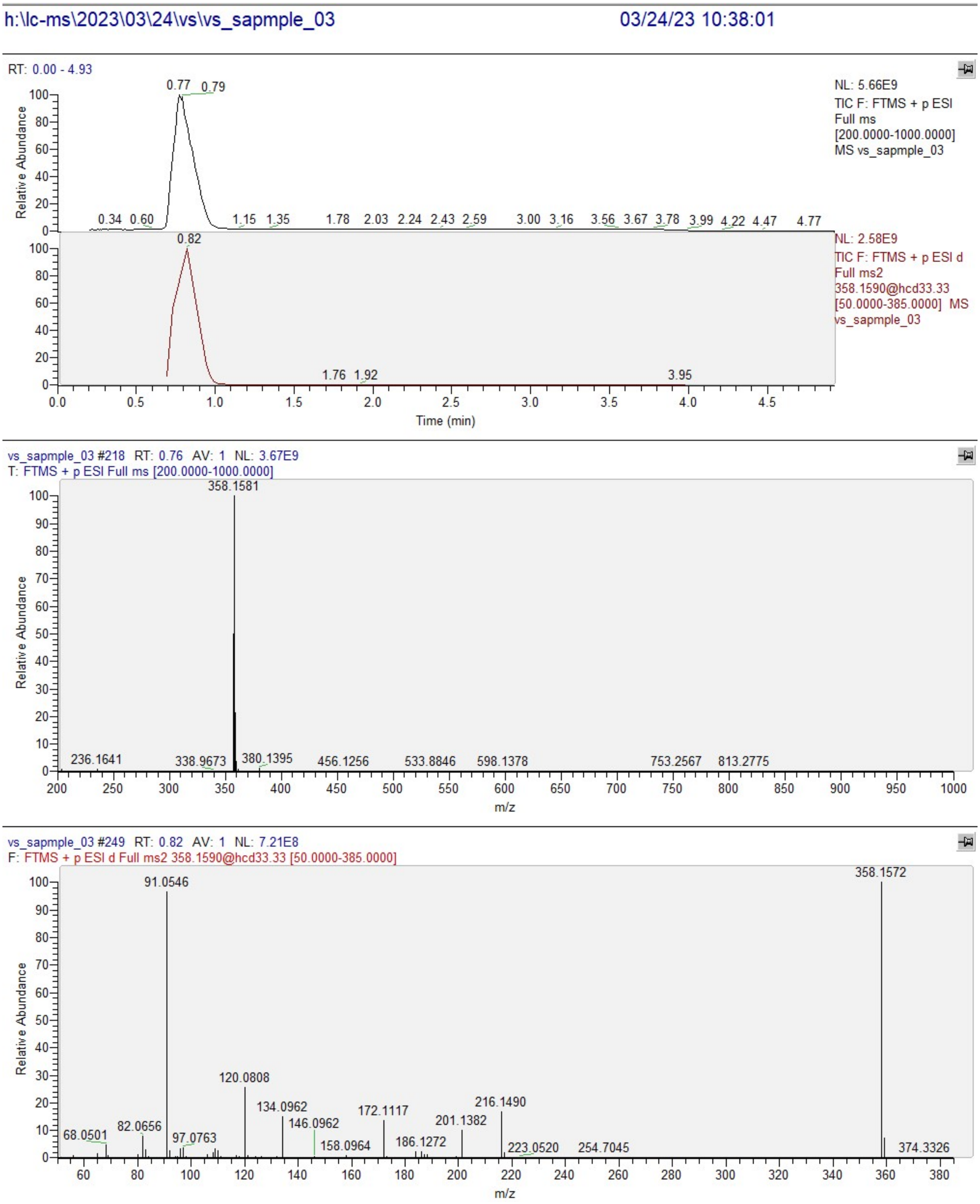

Figure S62. HRMS of **5a**

20. 4-methoxy-N'-[(E)-(5-methoxy-1-methyl-1H-indol-3-yl)methylidene]benzenesulfonohydrazide, **5b**

Yield: 78%; m.p. 145-146◦C. ^{1}H NMR (600 MHz, DMSO-d_6): δ = 3.74 (s, 3H, NCH_3), 3.77 (s, 3H, OCH_3), 3.80 (s, 3H, OCH_3), 6.86 (dd, J=2.6, 8.8 Hz, 1H, H-6), 7.11 (d, J=9.0 Hz, 2H, H-3' and H-5'), 7.36 (d, J=8.8 Hz, 1H, H-7), 7.47 (d, J=2.5 Hz, 1H, H-4), 7.64 (s, 1H, H-2), 7.85 (d, J=8.8 Hz, 2H, H-2' and H-6'), 8.05 (s, 1H, CH), 10.75 (s, 1H, NH).

^{13}C NMR (151 MHz, DMSO-d_6): δ = 32.90 (NCH_3), 55.22 (OCH_3), 55.64 (OCH_3), 103.31 (C-4), 109.71 (C-3), 111.01 (C-7), 112.54 (C-6), 114.20 (C-3' and C-5'), 124.90 (C-3a), 129.49 (C-1'), 130.83 (C-2' and C-6'), 132.52 (C-7a), 134.20 (C-2), 145.01 (CH), 154.66 (C-4), 162.45 (C-4'). HRMS (ESI) m/z: calcd: [M+H]+ 374.11597. Found: [M+H]+ 374.1169

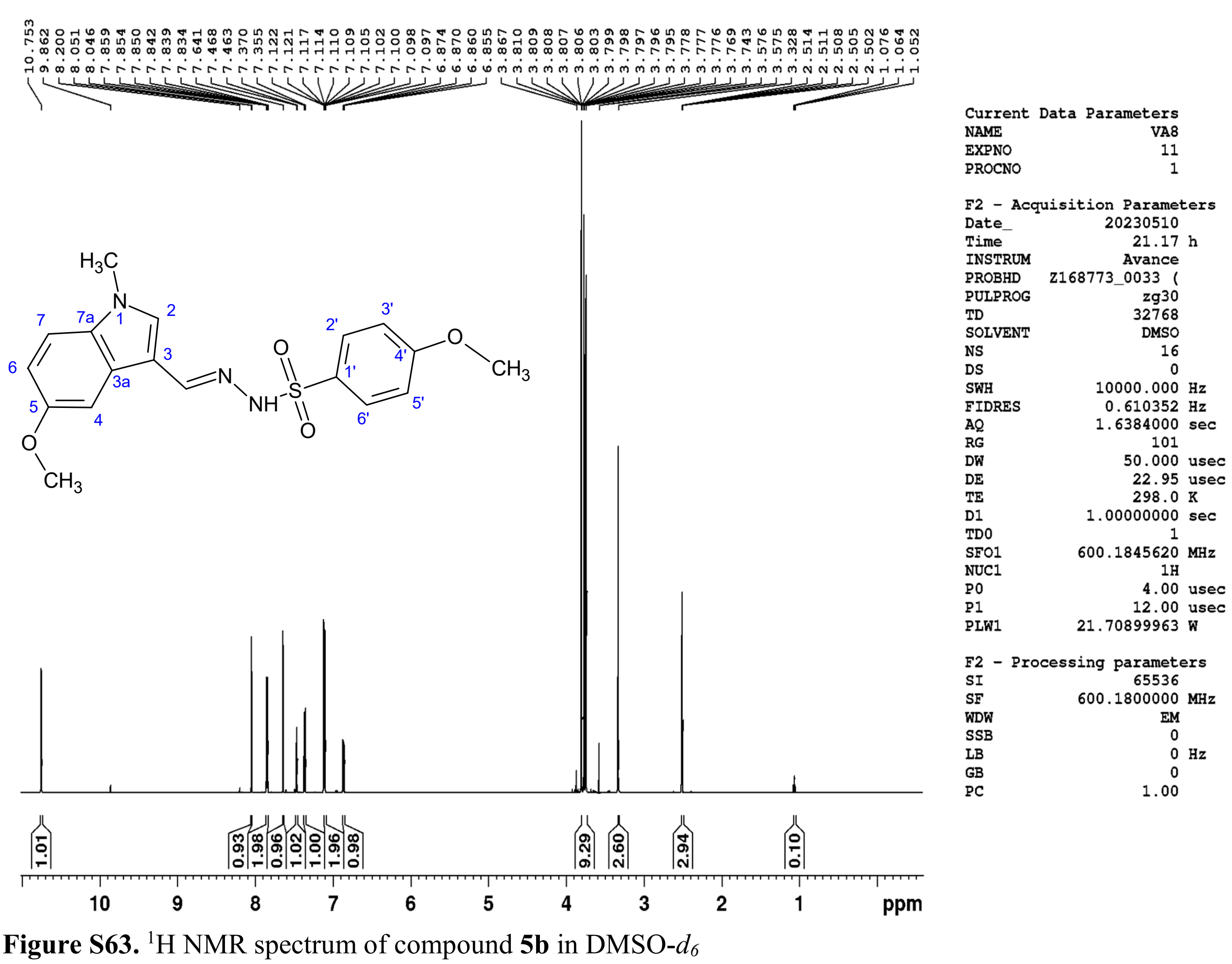

Figure S63. ^{1}H NMR spectrum of compound **5b** in DMSO-d_6

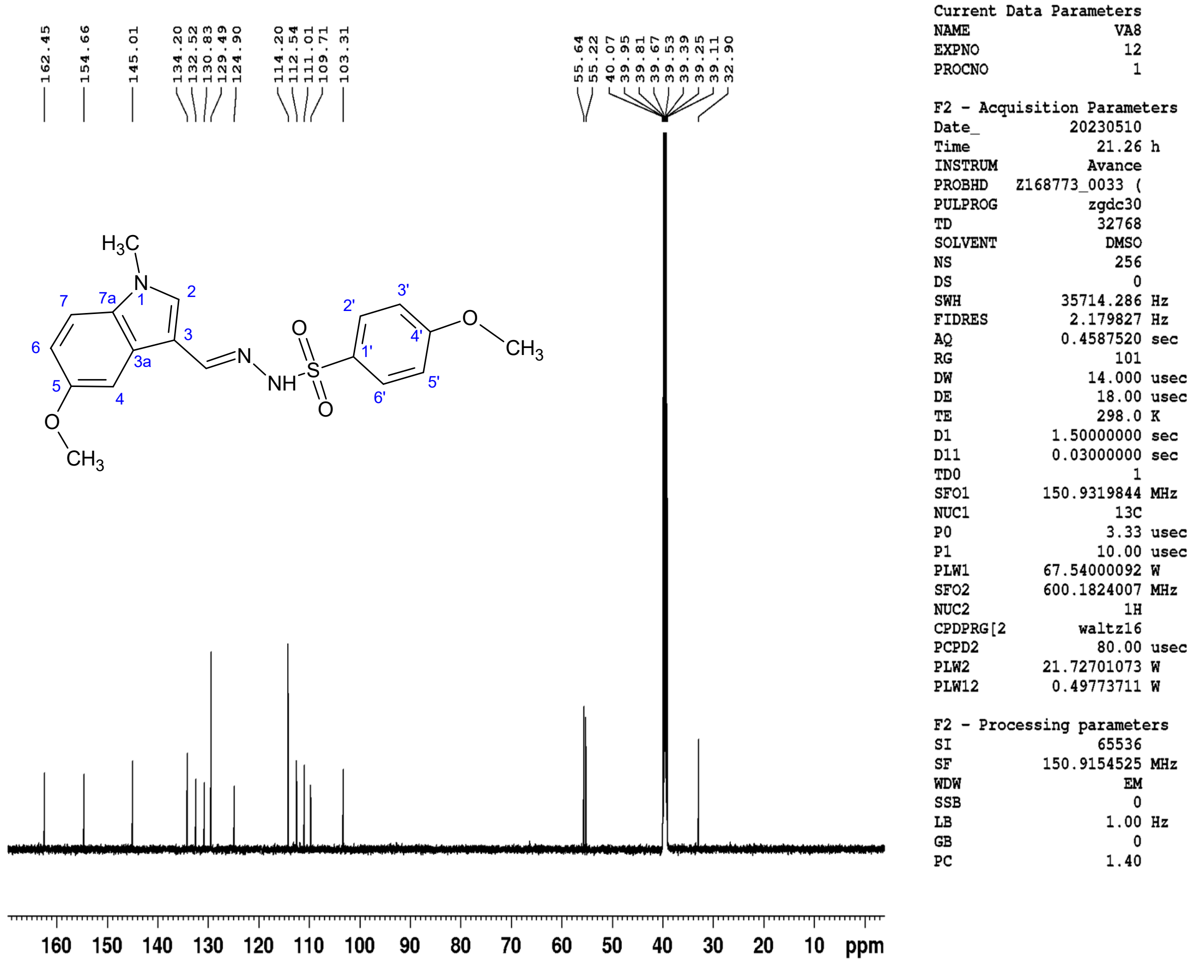

Figure S64. ^{13}C NMR spectrum of compound **5b** in DMSO-d_6

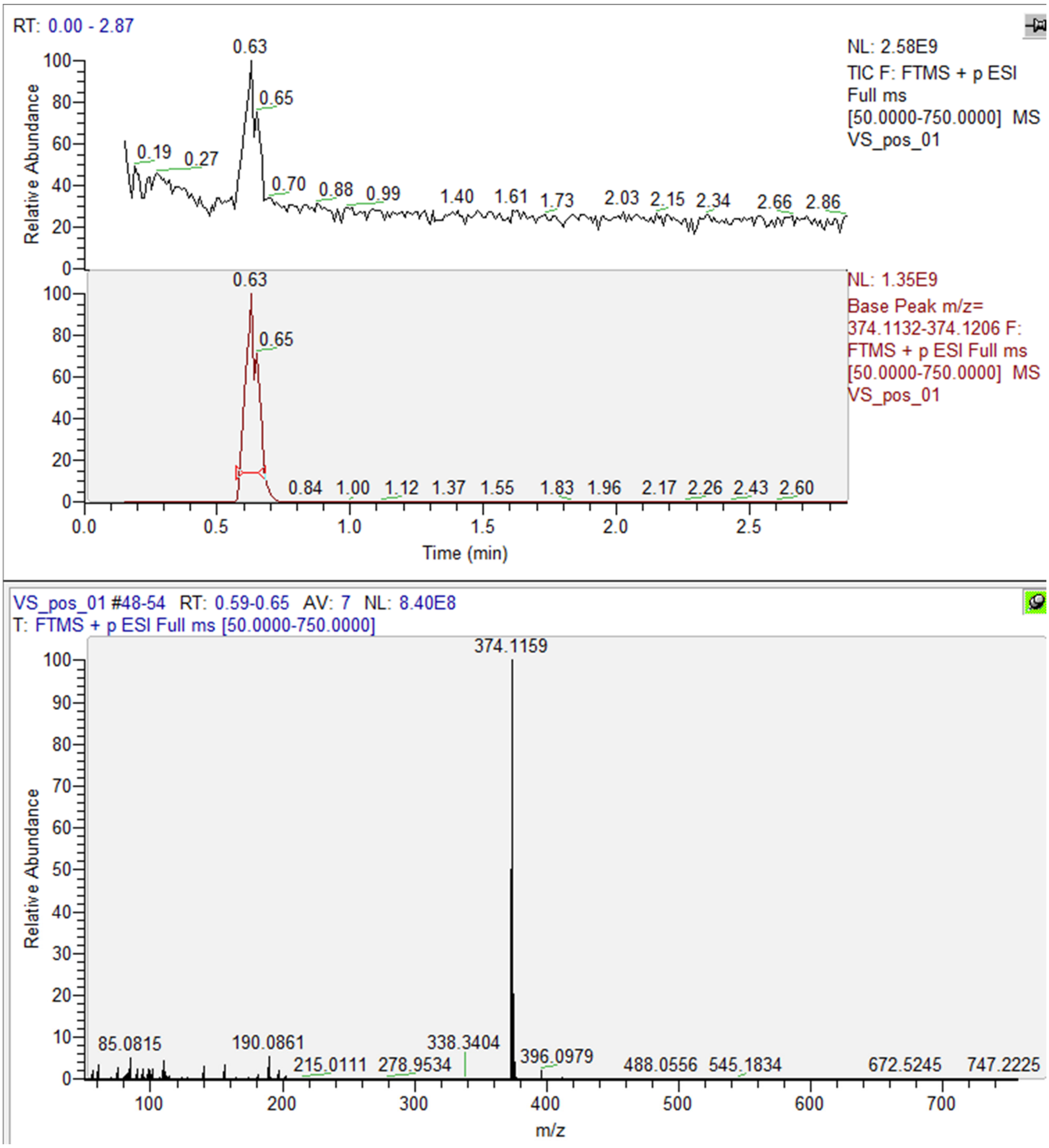

Figure S65. HRMS of compound **5b**

21. 4-methoxy-N'-[(E)-(5-methoxy-1H-indol-3-yl)methylidene]benzenesulfonohydrazide, **5c**

Yield: 53%; m.p. 159-163◦C. ^{1}H NMR (400 MHz, DMSO-d$_6$): δ = 3.76 (s, 3H, OCH$_3$), 3.80 (s, 3H, OCH$_3$), 6.80 (dd, J=2.6, 8.8 Hz, 1H, H-6), 7.11 (d, J=9.0 Hz, 2H, H-3' and H-5'), 7.29 (d, J=8.8 Hz, 1), 7.46 (d, J=2.6 Hz, 1H, H-4), 7.66 (d, J=2.8 Hz, 1H, H-2), 7.85 (d, J=9.0 Hz, 2H, H-2' and H-6'), 8.08 (s, 1H, CH), 10.76 (s, 1H, NH), 11.37 (d, J=2.2 Hz, 1H, NH).

^{13}C NMR (100 MHz, DMSO-d$_6$): δ = 55.15 (OCH$_3$), 55.63 (OCH$_3$), 103.12 (C-4), 110.85 (C-3), 112.48 (C-7), 112.59 (C-6), 114.19 (C-3' and C-5'), 124.49 (C-3a), 129.48 (C-1'), 130.73 (C-2' and C-6'), 130.84 (C-7a), 131.78 (C-2), 145.46 (CH), 154.35 (C-4), 162.43 (C-4'). HRMS (ESI) m/z: calcd: [M+H]+ 360.10125. Found: [M+H]+ 360.10037

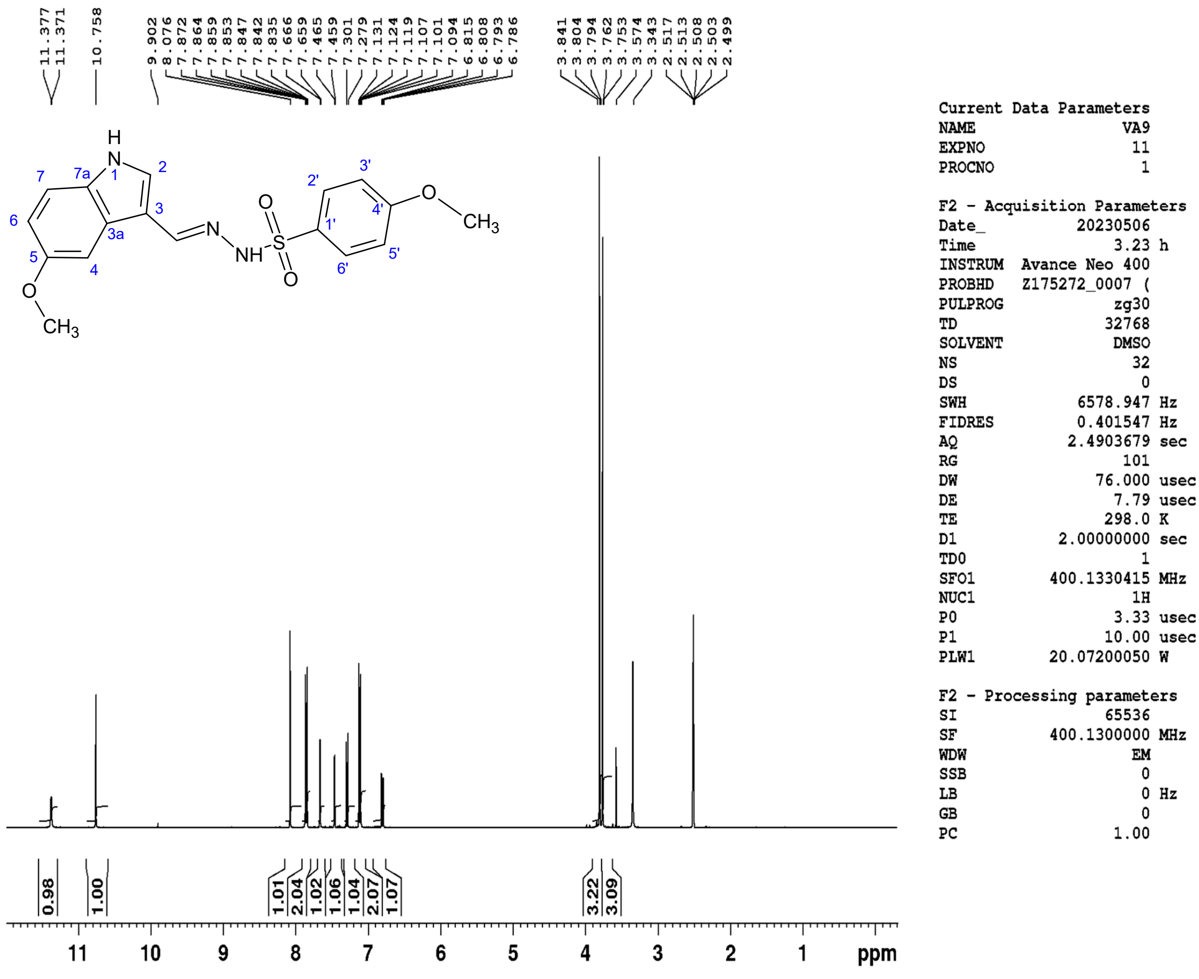

Figure S66. ^{1}H NMR spectrum of compound **5c** in DMSO-d_6

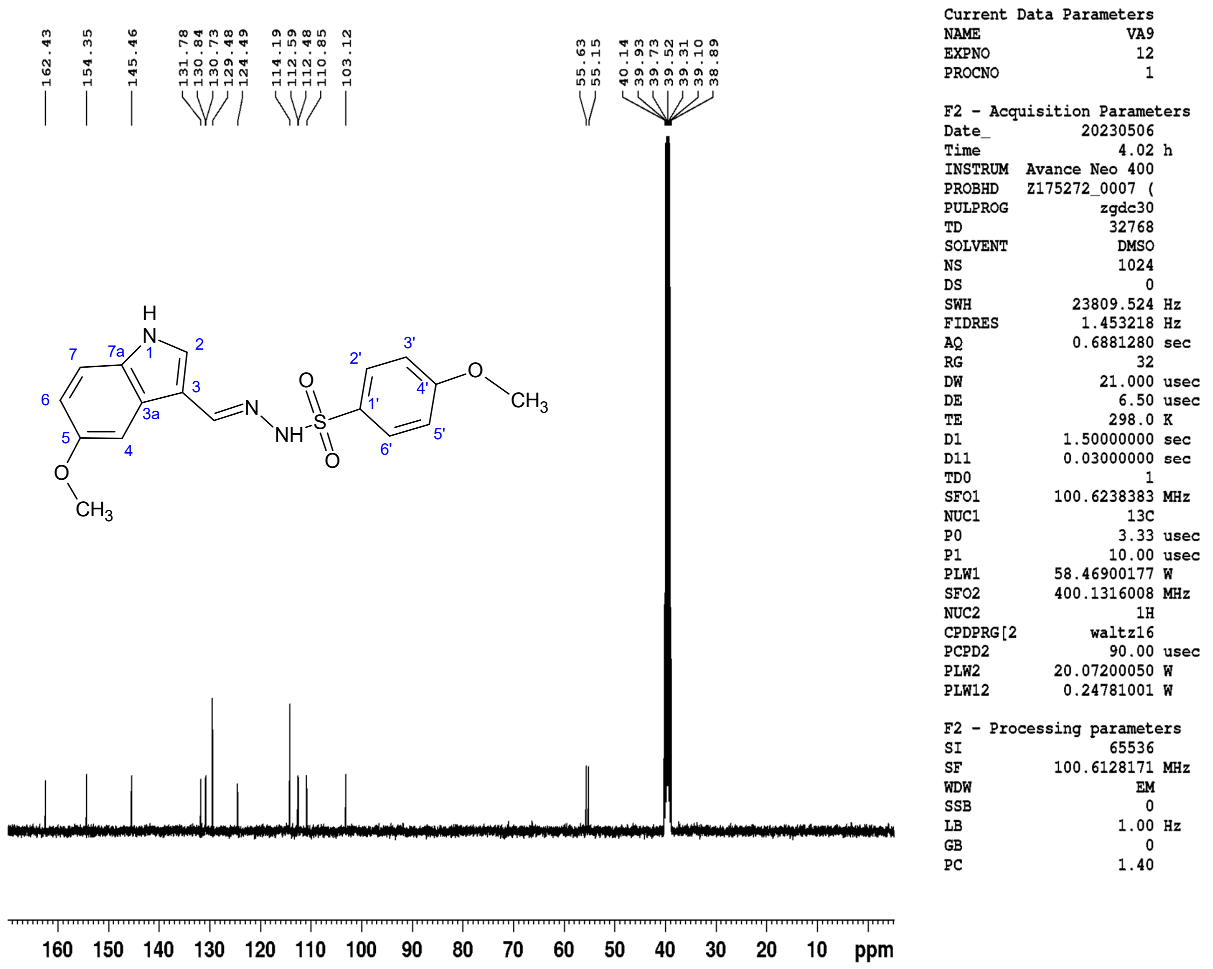

Figure S67. ^{13}C NMR spectrum of compound **5c** in DMSO-d_6

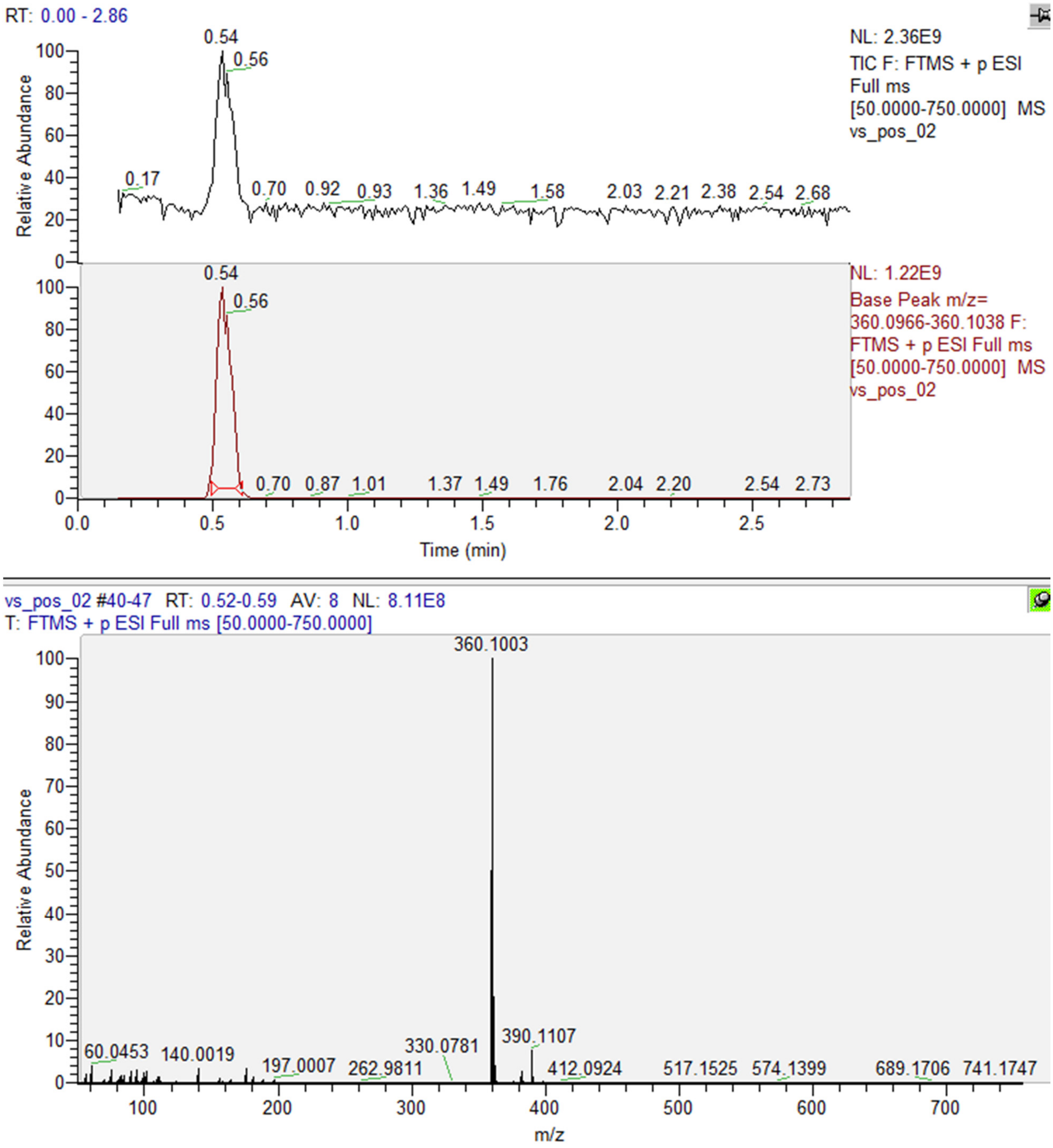

Figure S68. HRMS of compound **5c**

22. N'-[(E)-(1-benzyl-1H-indol-3-yl)methylidene]benzenesulfonohydrazide, **5d**

Yield: 89%; m.p. 146-149◦C.^{1}H NMR (600 MHz, DMSO-d$_6$): δ = 5.41 (s, 3H, CH_2), 7.15 (t, J=7.4 Hz, 1H, H-5), 7.18 (ddd, J=1.2, 7.1, 8.2 Hz, 1H, H-6), 7.21 (d, J=7.0 Hz, 2H, H-2' and H-6'), 7.24 (t, J=7.3 Hz, 1H, H-4'), 7.30 (t, J=7.3 Hz, 2H, H-3' and H-5'), 7.48 (d, J=8.1 Hz, 1H, H-7), 7.59-7.65 (m, 3H, H-3'', H-4''and H-5''), 7.90 (s, 1H, H-2), 7.93 (dd, J=1.0, 7.6 Hz, 2H, H-2'' and H-6''), 7.97 (d, J=7.8 Hz, 1H, H-4), 8.10 (s, 1H, CH), 11.01 (s, 1H, NH).

^{13}C NMR (151 MHz, DMSO-d$_6$): δ = 49.27 (CH_2), 110.65 (C-3), 110.67 (1C, s), 120.89 (C-5), 121.78 (C-4), 122.79 (C-6), 124.61 (C-3a), 127.09 (C-2' and C-6''), 127.28 (C-2'' and C-6''), 127.51 (C-4'), 128.59 (C-3' and C-5'), 129.05 (C-3'' and V-5''), 132.82 (C-4''), 133.50 (C-2), 136.75 (C-7a), 137.44 (C-1'), 139.13 (C-1''), 144.60 (CH). HRMS (ESI) m/z: calcd: [M+H]+ 390.127073. Found: [M+H]+ 390.12604

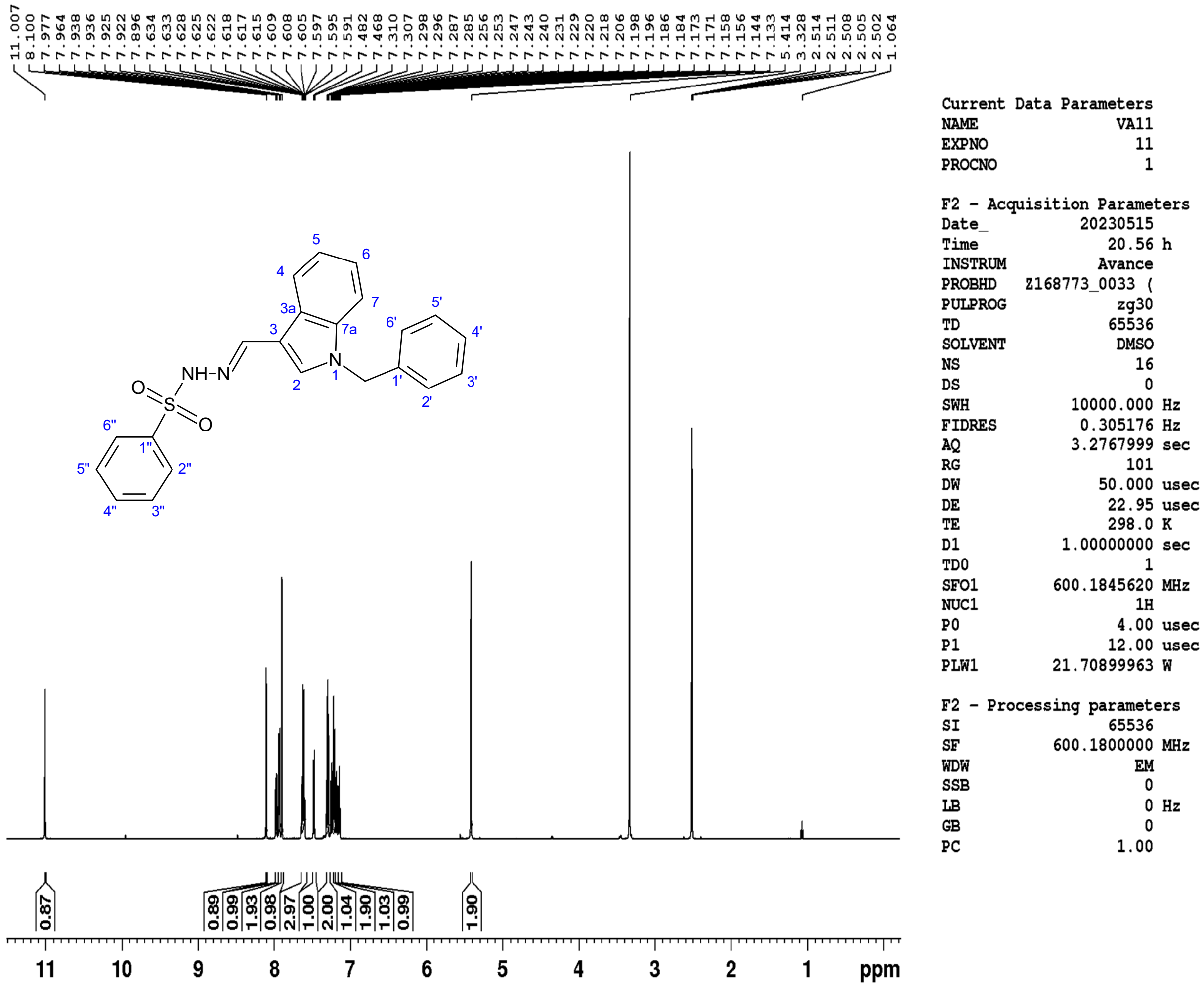

Figure S69. ^{1}H NMR spectrum of compound **5d** in DMSO-d_6

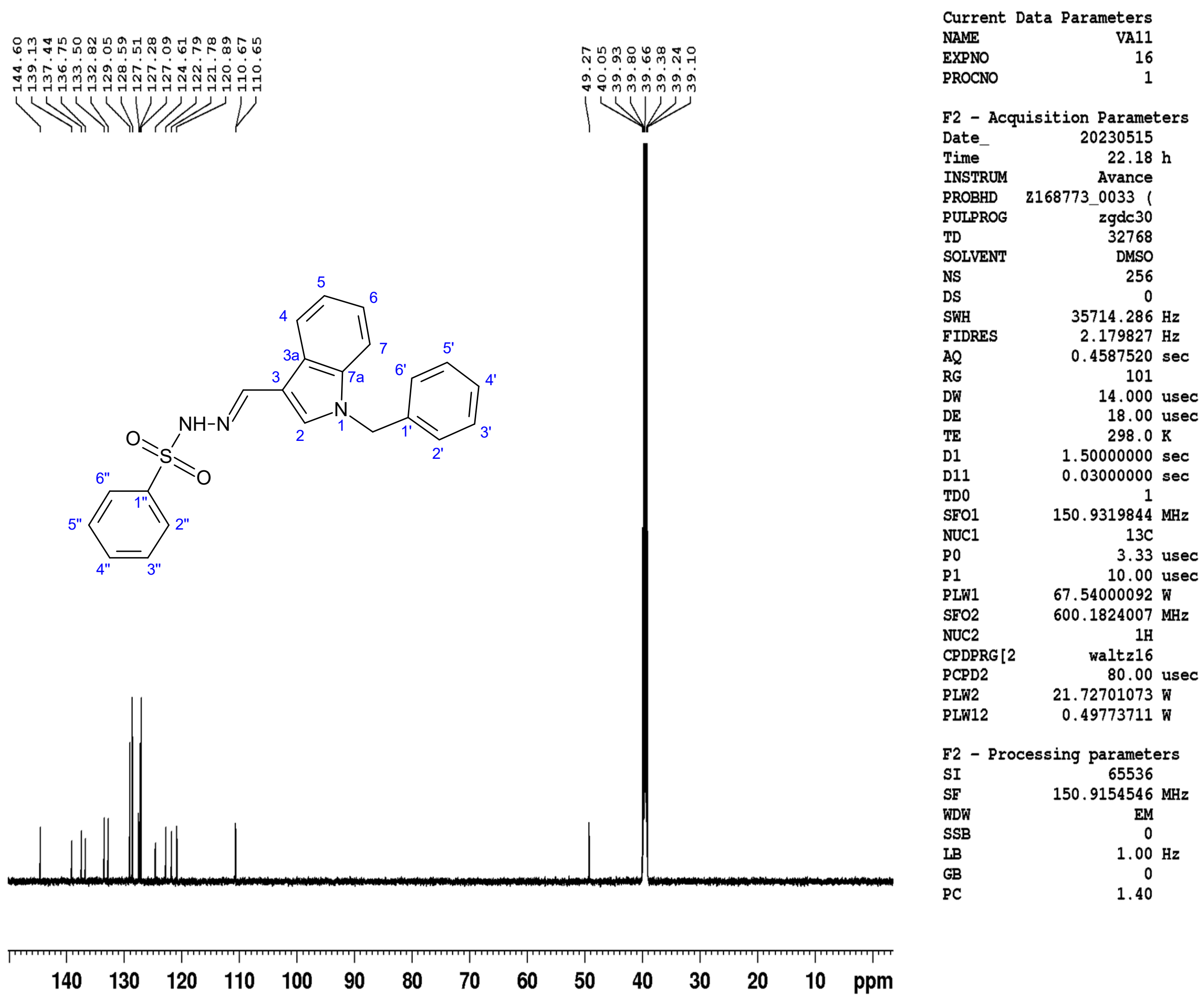

Figure S70. ^{13}C NMR spectrum of compound **5d** in DMSO-d_6

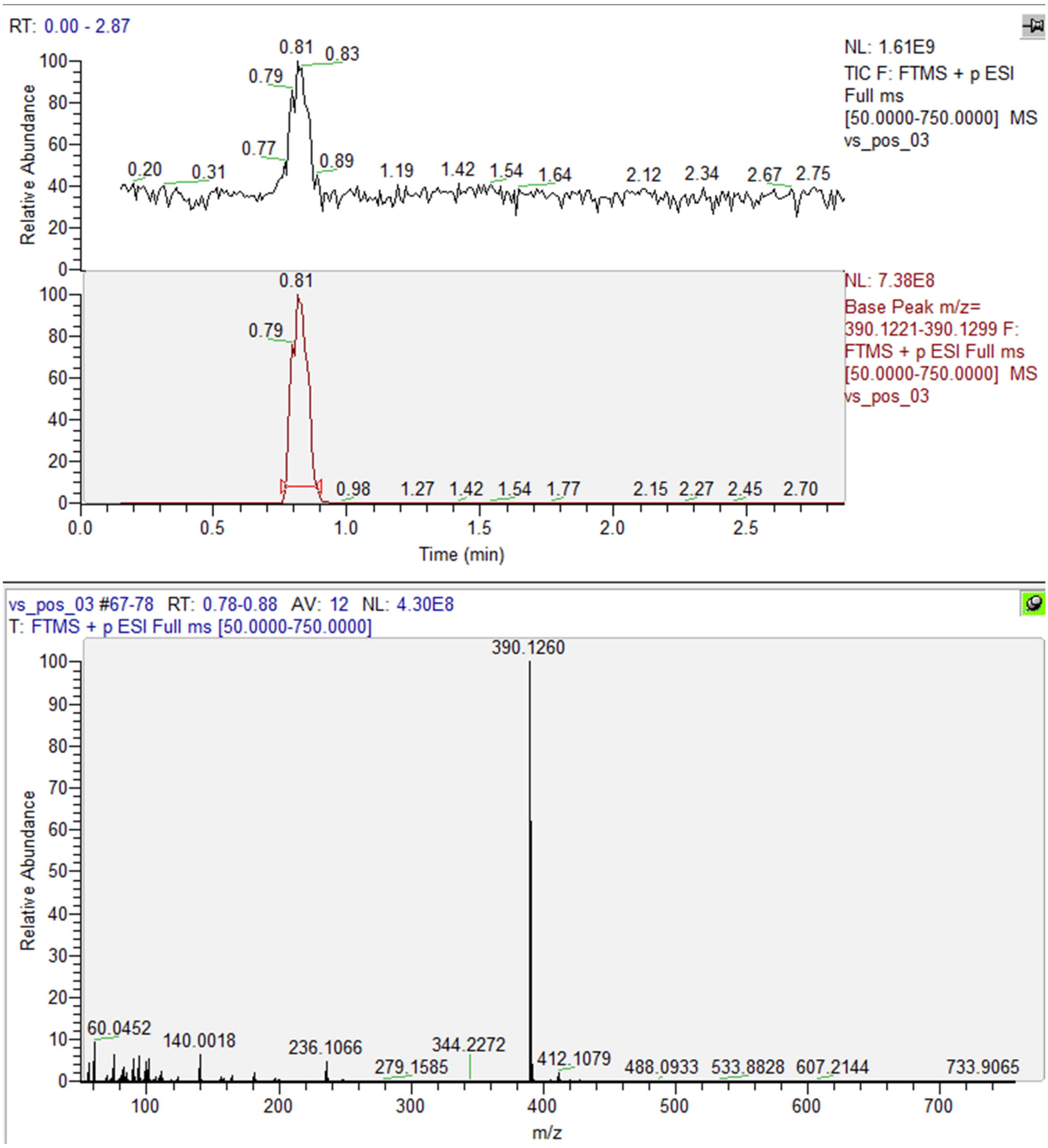

Figure S71. HRMS of compound **5d**

23. 4-methoxy-N'-[(E)-(1-methyl-1H-indol-3-yl)methylidene]benzenesulfonohydrazide, 5e

Yield: 66%; m.p. 170-175◦C. ^{1}H NMR (600 MHz, DMSO-d6): δ = 3.78 (s, 3H, NCH_3), 3.79 (s, 3H, OCH_3), 7.11 (d, J=9.0 Hz, 2H, H-3' and H-5'), 7.17 (t, J=7.5 Hz, 1H, H-5), 7.24 (ddd, J=1.1, 7.1, 8.1 Hz, 1H, H-6), 7.46 (d, J=8.2 Hz, 1H, H-7), 7.69 (s, 1H, H-2), 7.84 (d, J=9.0 Hz, 2H, H2' and H-6'), 7.97 (d, J=7.9 Hz, 1H, H-4), 8.05 (s, 1H, CH), 10.76 (s, 1H, NH).

^{13}C NMR (151 MHz, DMSO-d6): δ = 32.73 (NCH_3), 55.61 (OCH_3), 110.11 (C-3), 110.17 (C-7), 114.18 (C-3' and H-5'), 120.74 (C-5), 121.66 (C-4), 122.62 (C-6), 124.40 (C-3a), 129.48 (C-2' and C-6'), 130.81 (C-2' and C-6'), 133.89 (C-1'), 137.42 (C-7a), 144.44 (CH), 162.44 (C-4'). HRMS (ESI) m/z: calcd: [M+H]+ 344.106338. Found: [M+H]+ 344.1055

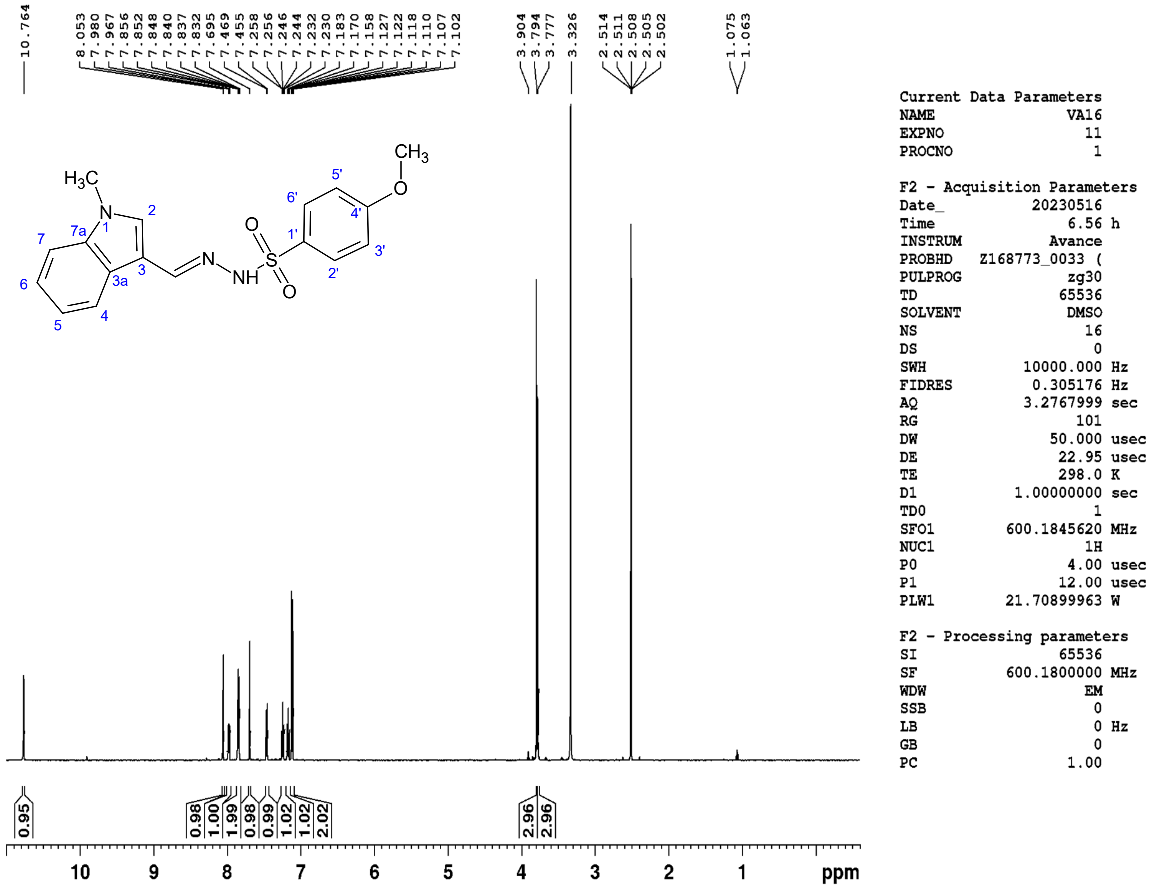

Figure S72. ^{1}H NMR spectrum of compound **5e** in DMSO-d_6

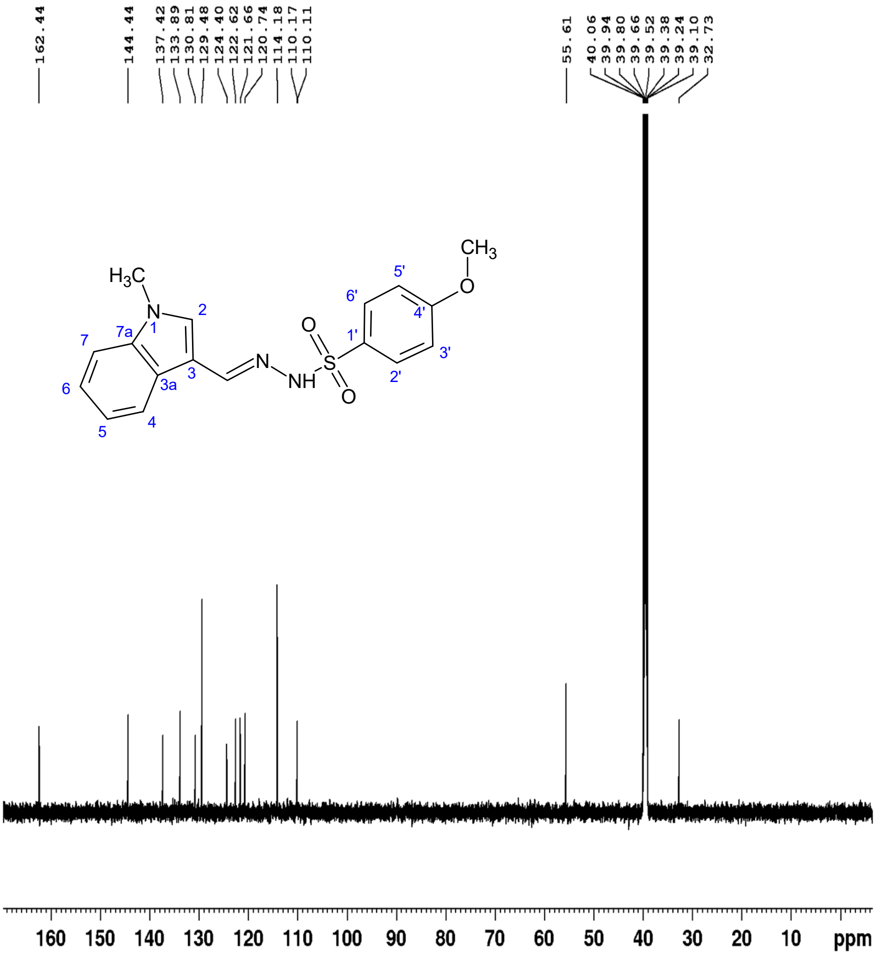

Current Data Parameters
NAME VA16
EXPNO 16
PROCNO 1

F2 - Acquisition Parameters
Date_ 20230516
Time 8.18 h
INSTRUM Avance
PROBHD Z168773_0033 (
PULPROG zgdc30
TD 32768
SOLVENT DMSO
NS 256
DS 0
SWH 35714.286 Hz
FIDRES 2.179827 Hz
AQ 0.4587520 sec
RG 101
DW 14.000 usec
DE 18.00 usec
TE 298.0 K
D1 1.50000000 sec
D11 0.03000000 sec
TD0 1
SFO1 150.9319844 MHz
NUC1 13C
P0 3.33 usec
P1 10.00 usec
PLW1 67.54000092 W
SFO2 600.1824007 MHz
NUC2 1H
CPDPRG[2 waltz16
PCPD2 80.00 usec
PLW2 21.72701073 W
PLW12 0.49773711 W

F2 - Processing parameters
SI 65536
SF 150.9154542 MHz
WDW EM
SSB 0
LB 1.00 Hz
GB 0
PC 1.40

Figure S73. ^{13}C NMR spectrum of compound **5e** in DMSO-d_6

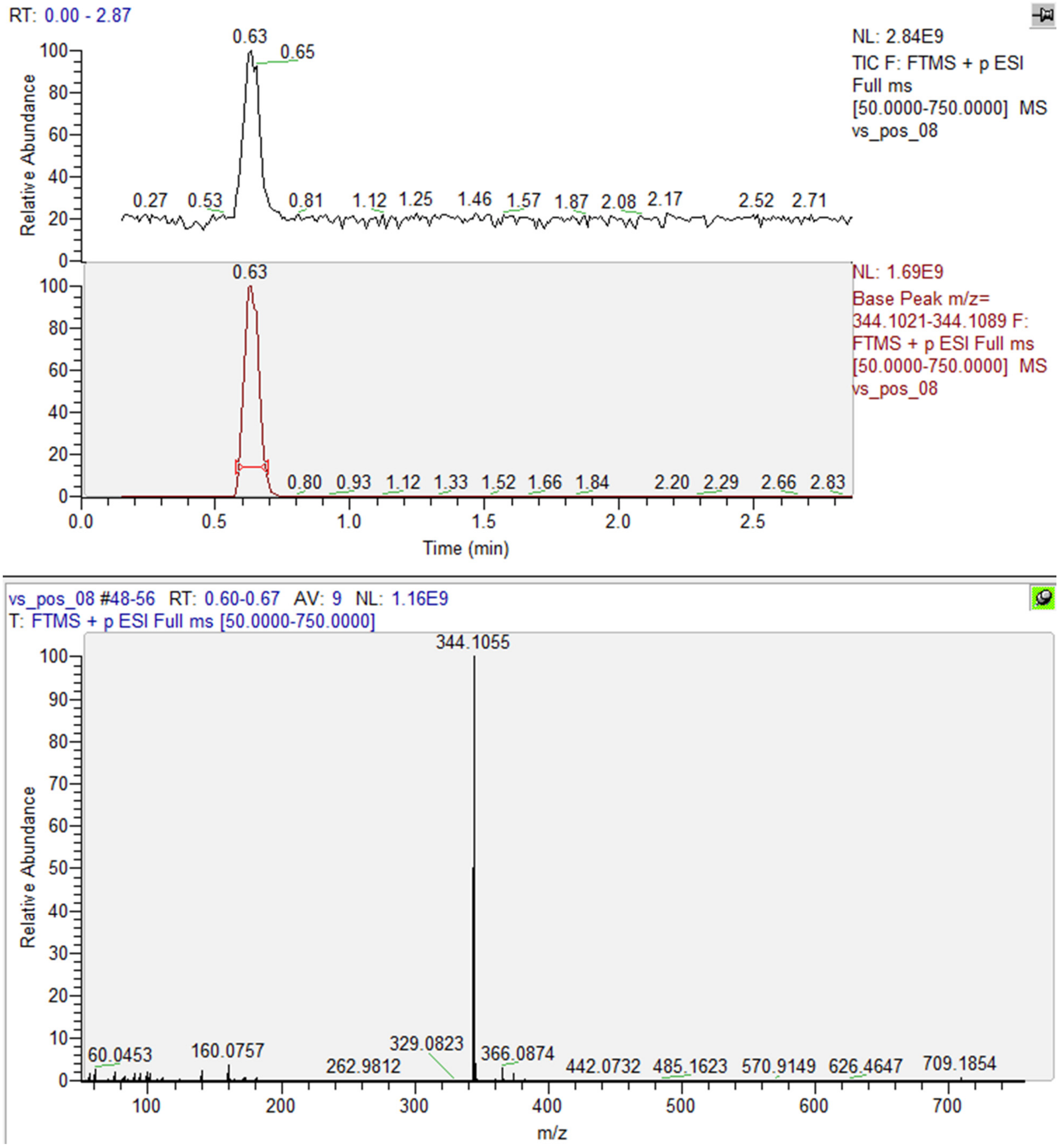

Figure S74. HRMS of compound **5e**

24. N'-[(E)-(5-methoxy-1-methyl-1H-indol-3-yl)methylidene]benzenesulfonohydrazide, 5f [2] ^{1}H NMR, ^{13}C NMR and HRMS spectra of compound 5f can be found in **https://doi.org/10.3390/molecules28052058**

25. N'-[(E)-(1-benzyl-1H-indol-3-yl)methylidene]-4-methylbenzenesulfonohydrazide, **5g**

Yield: 79%; m.p. 145-146◦C.^{1}H NMR (400 MHz, DMSO-d6): δ = 2.34 (s, 3H, CH_3), 5.41 (s, 2H, CH_2), 7.13-7.32 (m, 7H, H-5, H6, H-2'', H-3'', H-4'', H-5'' and H-6''), 7.39 (d, J=7.9 Hz, 2H, H-3' and H-5'), 7.48 (dd, J=1.3, 7.1 Hz, 1H, H-7), 7.81 (d, J=8.2 Hz, 2H, H-2' and H-6'), 7.89 (s, 1H, H-2), 7.99 (d, J=7.3 Hz, 1H, H-4), 8.08 (s, 1H, CH), 10.92 (s, 1H, NH).

^{13}C NMR (100 MHz, DMSO-d6): δ = 20.95 (CH_3), 49.26 (CH_2), 110.66 (C-7), 110.72 (C-3), 120.88 (C-5), 121.83 (C-4), 122.78 (C-6), 124.63 (C-4a), 127.08 (C-2'' and C-6''), 127.33 (C-2' and C-6'), 127.51 (C-4''), 128.59 (C-3'' and C-5''), 129.47 (C-3' and C-5'), 133.41 (C-2), 136.26 (C-1'), 136.74 (C-7a), 137.46 (C-1''), 143.16 (C-4'), 144.32 (C-H).HRMS (ESI) m/z: calcd: [M+H]+ 404.142723. Found: [M+H]+ 404.1425

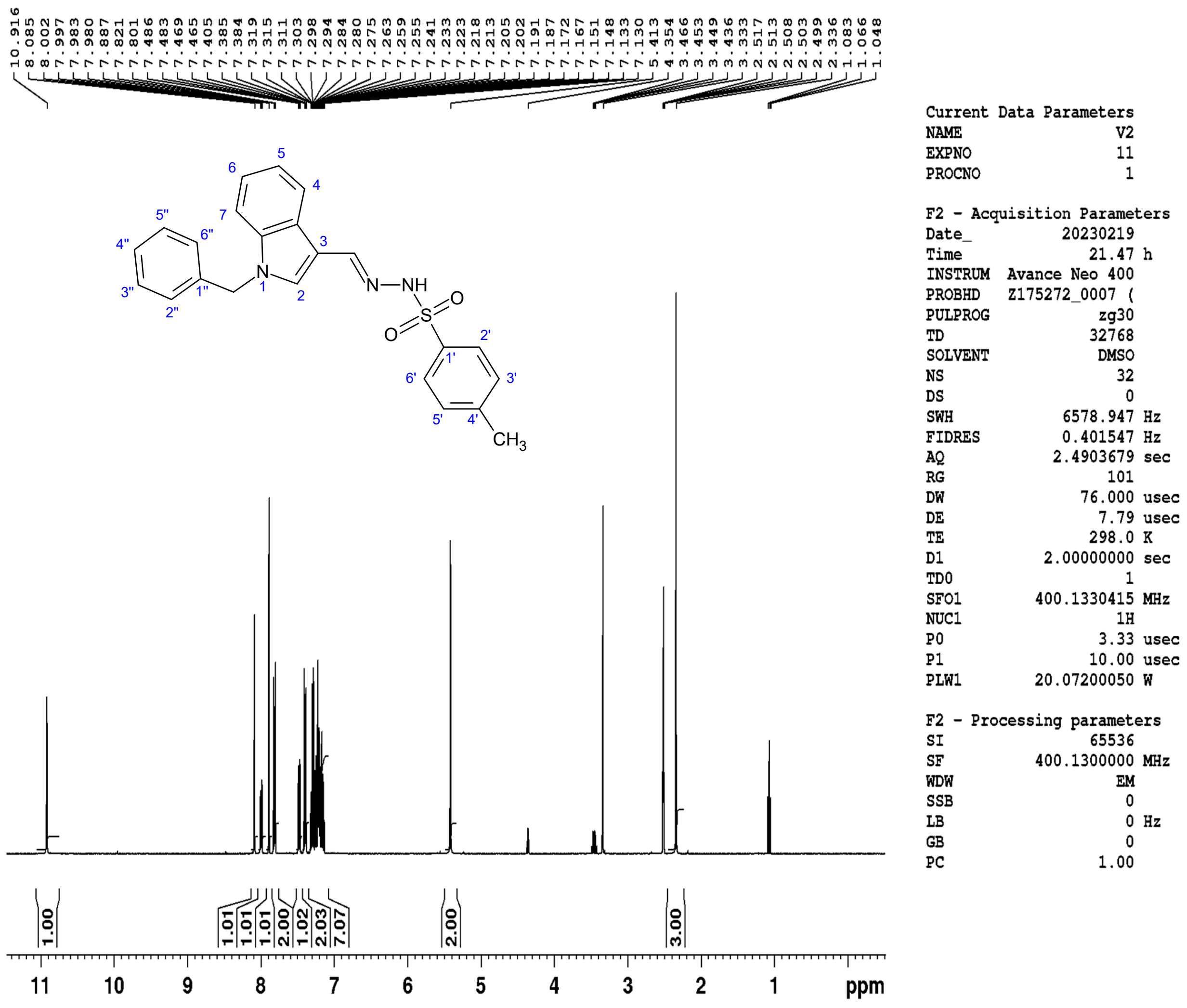

Figure S75. ^{1}H NMR spectrum of compound **5g** in DMSO-d_6

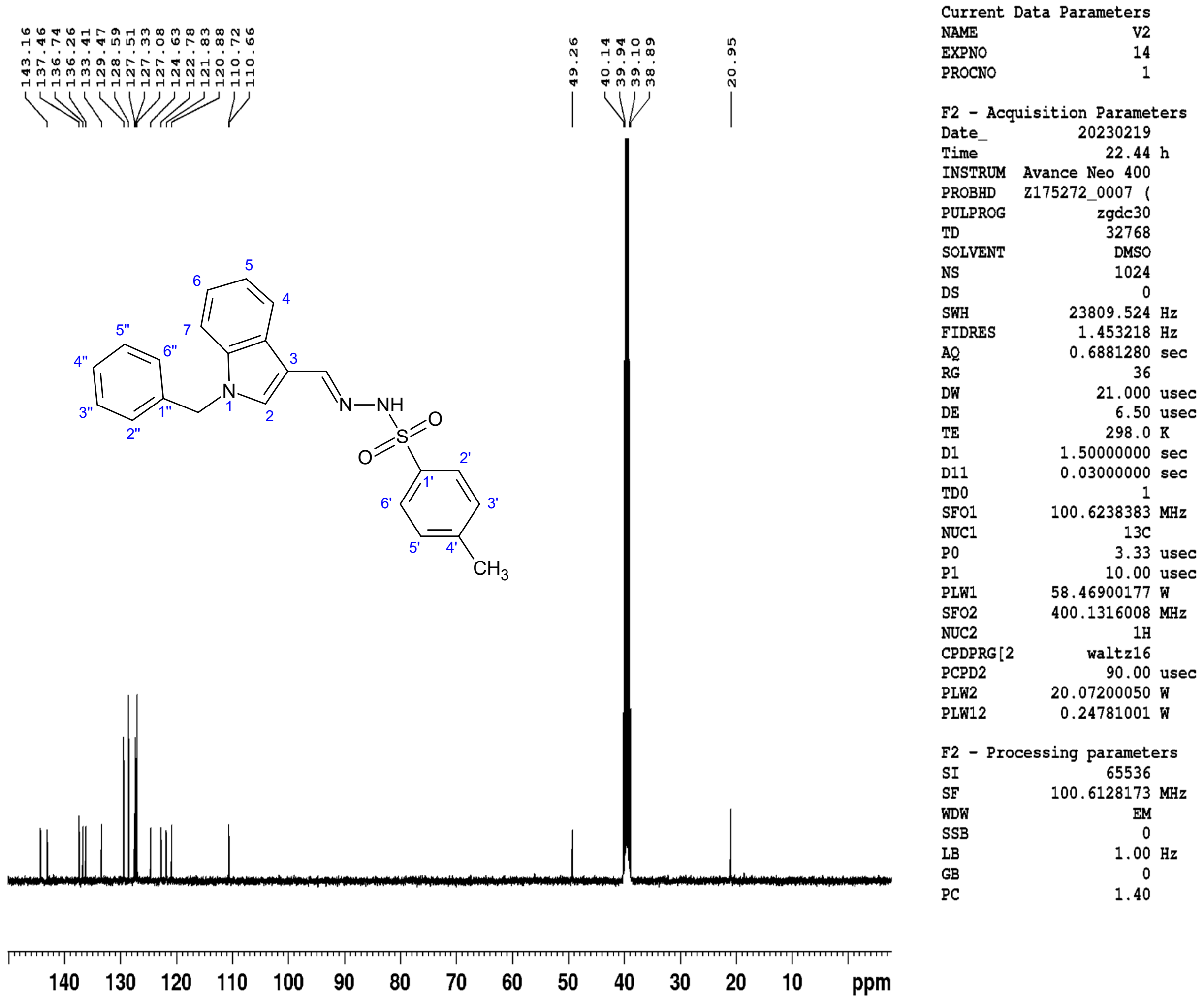

Figure S76. ^{13}C NMR spectrum of compound **5g** in DMSO-d_6

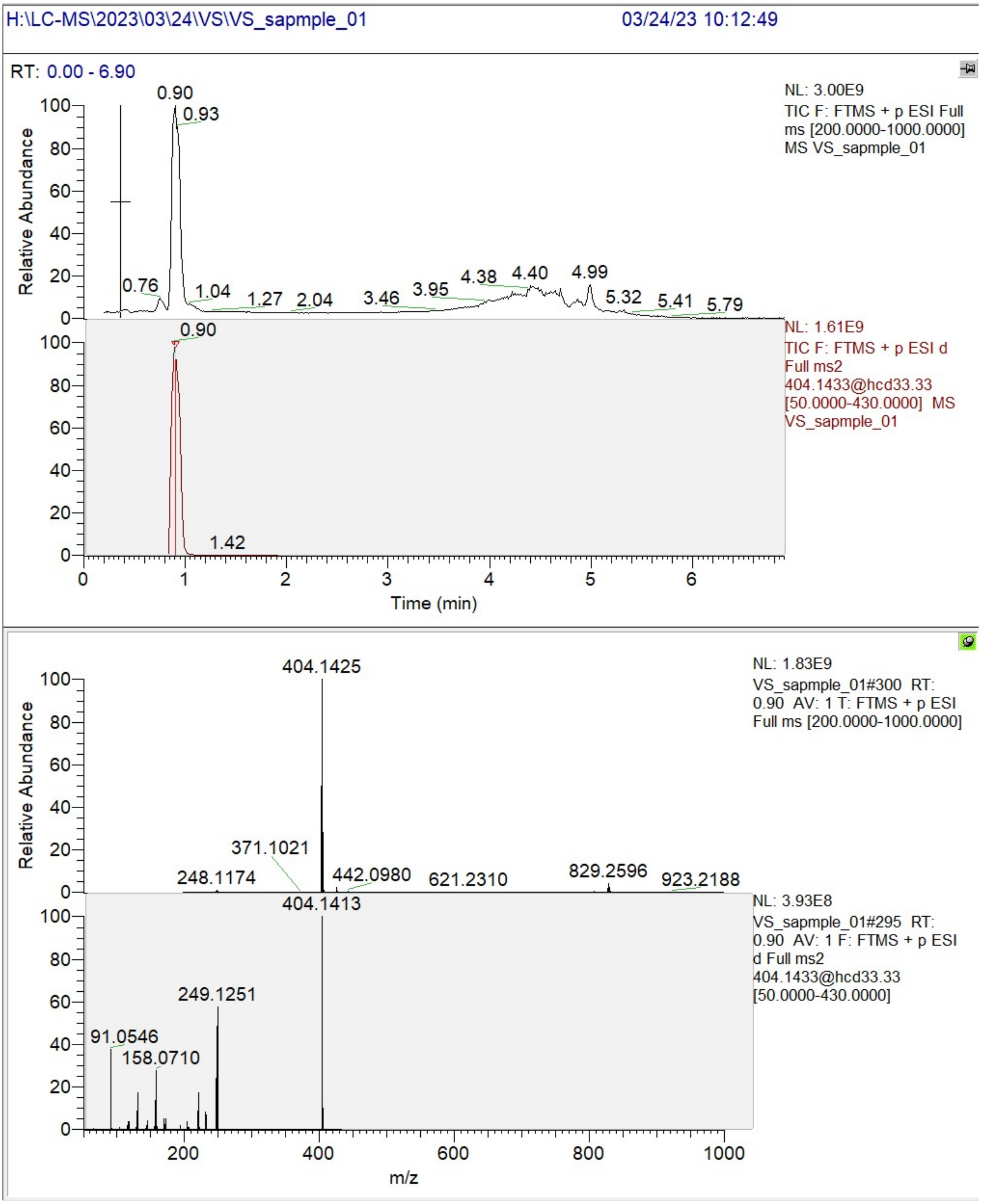

Figure S77. HRMS of compound **5g**

26. N'-[(E)-(3,4-dimethoxyphenyl)methylidene]-4-methylbenzenesulfonohydrazide, **5h** [3] ^{1}H NMR, ^{13}C NMR and HRMS spectra of compound **5h** can be found in https://doi.org/10.3390/molecules28052058

27. N’-[(E)-(3,4-dimethoxyphenyl)methylidene]benzenesulfonohydrazide, **5i** [2] ^{1}H NMR, ^{13}C NMR and HRMS spectra of compound **5i** can be found in https://doi.org/10.3390/molecules28052058

***28. N'-{(E)-[5-(benzyloxy)-1H-indol-3-yl]methylidene}-4-methylbenzenesulfonohydrazide,* 5j**

Yield: 67%; m.p. 222-223◦C.^{1}H NMR (400 MHz, DMSO-d6): δ = 2.28 (s, 3H, CH_3), 5.03 (s, 2H, CH_2), 6.88 (dd, J=2.5, 8.8 Hz, 1H, H-6), 7.30 (d, J=8.8 Hz, 1H, H-7), 7.34 (d, J=8.1 Hz, 2H, H-3’ and H-5’), 7.36 (t, J=7.2 Hz, 1H, H-4’’), 7.43 (t, J=7.4 Hz, 2H, H-3’’ and H-5’’), 7.53 (d, J=7.2 Hz, 2H, H-2’’ and H-6’’), 7.57 (d, J=2.4 Hz, 1H, H-4), 7.68 (d, J=2.8 Hz, 1H, H-2), 7.81 (d, J=8.2 Hz, 2H, H-2’ and H-6’), 8.09 (s, 1H, CH), 10.84 (s, 1H, NH), 11.40 (d, J=2.0 Hz, 1H, NH).

^{13}C NMR (100 MHz, DMSO-d6): δ = 20.90 (CH_3), 69.68 (CH_2), 104.82 (C-4), 110.81 (C-3), 112.50 (C-6), 113.02 (C-7), 124.48 (C-4a), 127.37 (C-2’ and C-6’), 127.84 (C-4’’), 127.93 (C-2’’ and C-6’’), 128.46 (H-3’’ and H-5’’), 129.41 (C-3’ and C-5’), 130.96 (C-2), 131.98 (C-7a), 136.33 (C-1’), 137.35 (C-1’’), 143.08 (C-4’), 145.68 (CH), 153.38 (C-5).HRMS (ESI) m/z: calcd: [M+H]+ 404.142723. Found: [M+H]+ 420.1373

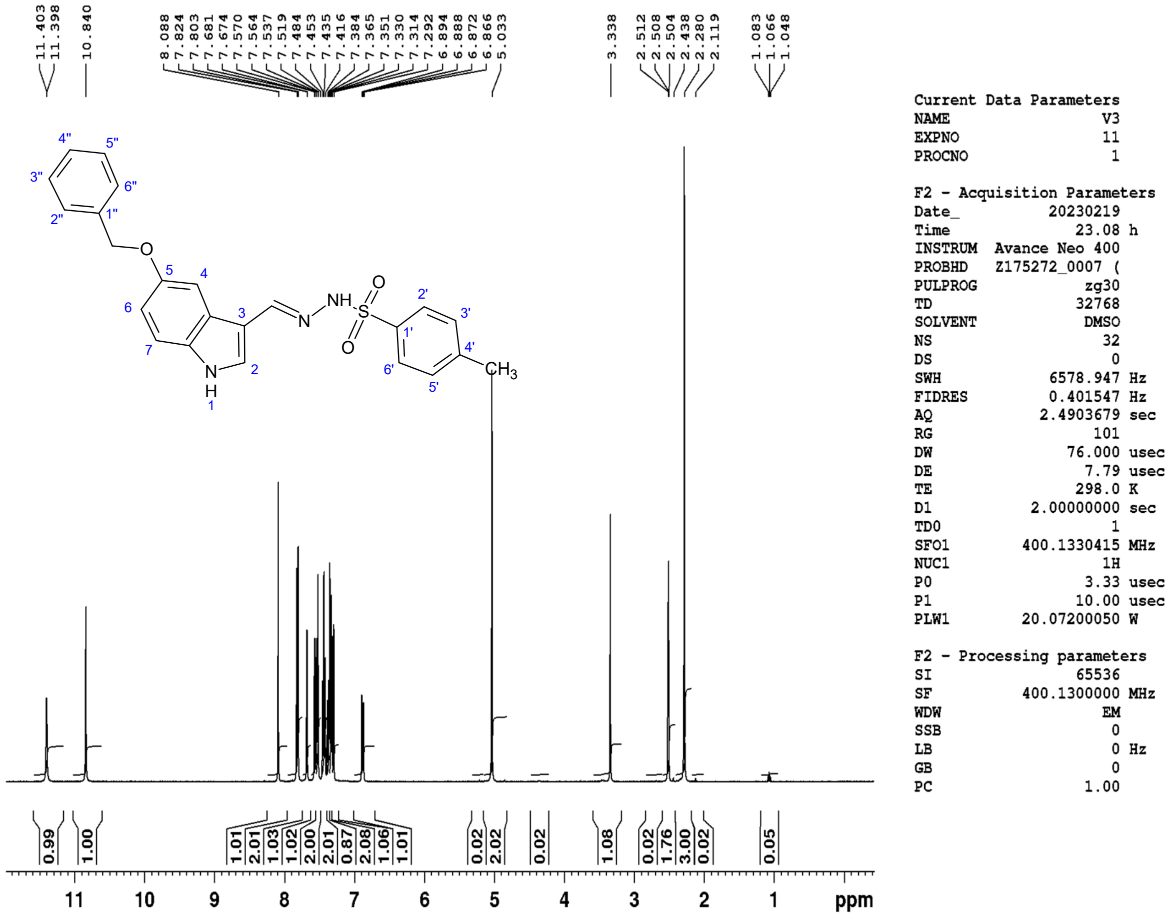

Figure S78. ^{1}H NMR spectrum of compound **5j** in DMSO-d_6

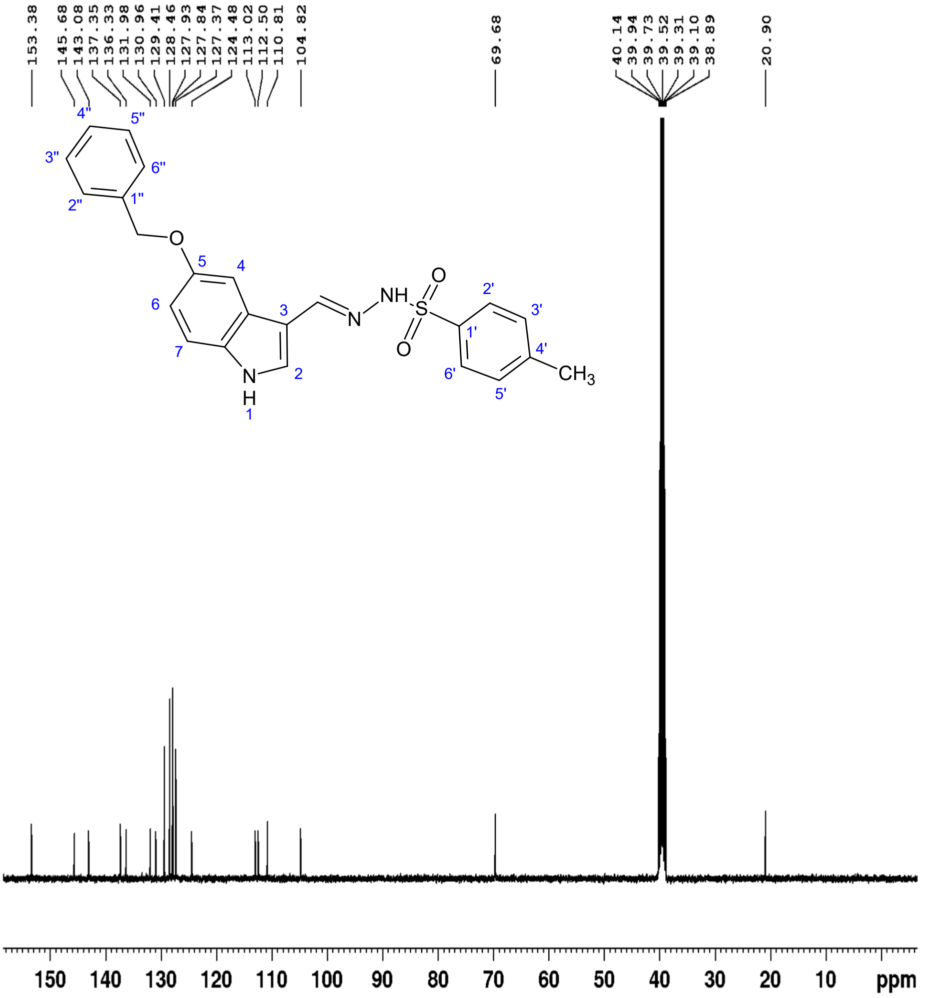

Current Data Parameters
NAME V3
EXPNO 14
PROCNO 1

F2 - Acquisition Parameters
Date_ 20230220
Time 0.04 h
INSTRUM Avance Neo 400
PROBHD Z175272_0007 (
PULPROG zgdc30
TD 32768
SOLVENT DMSO
NS 1024
DS 0
SWH 23809.524 Hz
FIDRES 1.453218 Hz
AQ 0.6881280 sec
RG 36
DW 21.000 usec
DE 6.50 usec
TE 298.0 K
D1 1.50000000 sec
D11 0.03000000 sec
TD0 1
SFO1 100.6238383 MHz
NUC1 13C
P0 3.33 usec
P1 10.00 usec
PLW1 58.46900177 W
SFO2 400.1316008 MHz
NUC2 1H
CPDPRG[2 waltz16
PCPD2 90.00 usec
PLW2 20.07200050 W
PLW12 0.24781001 W

F2 - Processing parameters
SI 65536
SF 100.6128171 MHz
WDW EM
SSB 0
LB 1.00 Hz
GB 0
PC 1.40

Figure S79. ^{13}C NMR spectrum of compound **5j** in DMSO-d_6

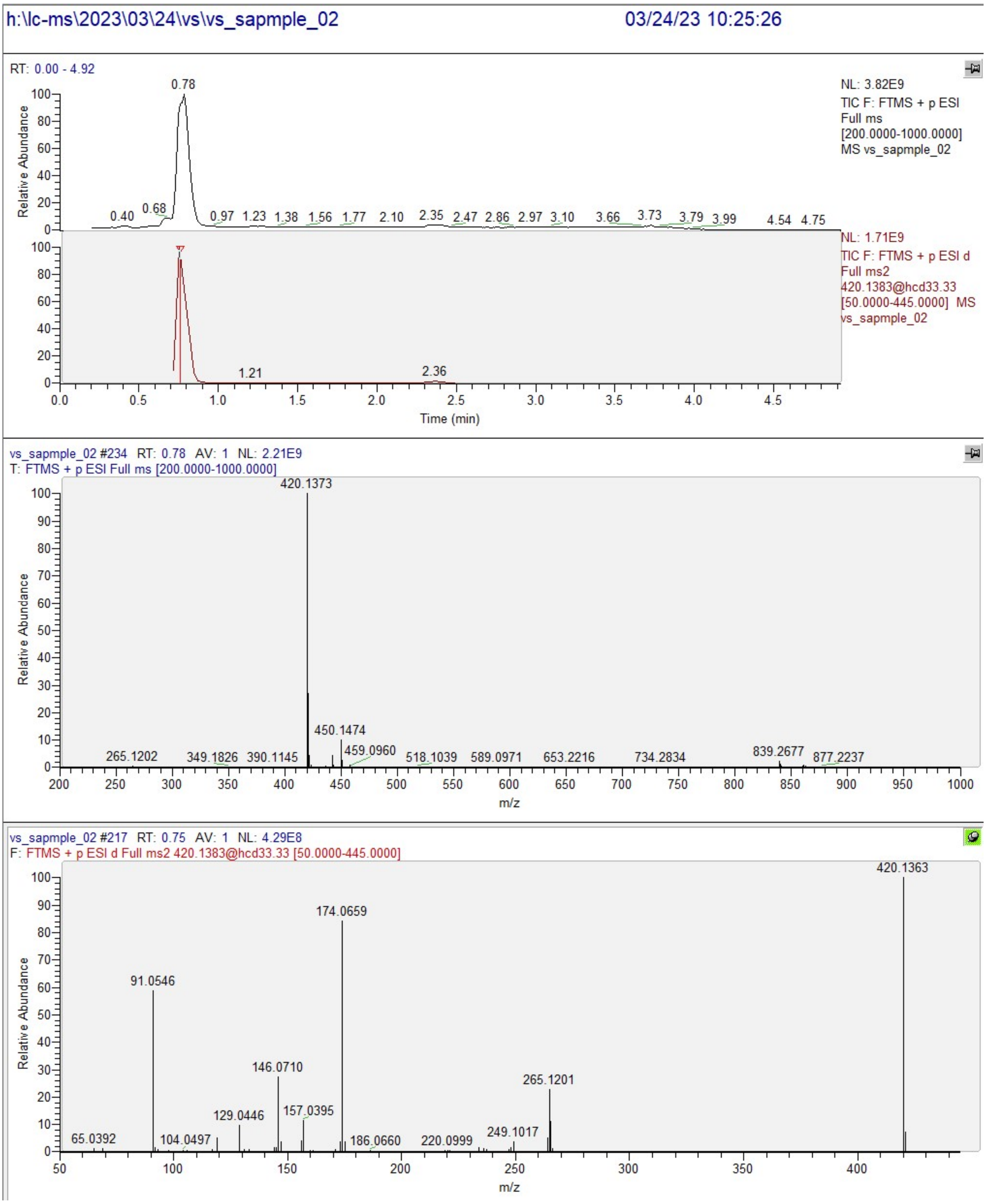

Figure S80. HRMS of compound **5j**

29. ***N'-((E)-[5-(benzyloxy)-1H-indol-3-yl]methylidenebenzenesulfonohydrazide, 5k*** [2] ^{1}H NMR, ^{13}C NMR and HRMS spectra of compound 5k can in **https://doi.org/10.3390/antibiotics11050562**

30. ***N'-[(E)-(4-chlorophenyl)methylidene]benzenesulfonohydrazide,*** **5l** [2] ^{1}H NMR, ^{13}C NMR and HRMS spectra of compound 5l can be found in **https://doi.org/10.3390/molecules28052058**

References

[1] V.R. Karabeliov, M.S. Kondeva-Burdina, N.G. Vassilev, K. Elena, V.T. Angelova, Neuroprotective evaluation of novel substituted 1, 3, 4-oxadiazole and aroylhydrazone derivatives, Bioorganic & Medicinal Chemistry Letters, 59 (2022) 128516.
[2] V.T. Angelova, T. Pencheva, N. Vassilev, E. K-Yovkova, R. Mihaylova, B. Petrov, V. Valcheva, Development of new antimycobacterial sulfonyl hydrazones and 4-methyl-1, 2, 3-thiadiazole-based hydrazone derivatives, Antibiotics, 11 (2022) 562.
[3] V.T. Angelova, T. Tatarova, R. Mihaylova, N. Vassilev, B. Petrov, Z. Zhivkova, I. Doytchinova, Novel Arylsulfonylhydrazones as Breast Anticancer Agents Discovered by Quantitative Structure-Activity Relationships, Molecules, 28 (2023) 2058.
